# A Central Somatic Transmission Mediates Proprioceptive Facilitation of Muscle Pain

**DOI:** 10.1002/advs.202514242

**Published:** 2026-04-23

**Authors:** Xiaoyu Zhang, Jiale Yang, Xi Wu, Jie Li, Fujian Lu, Yiman Li, Xingyu Du, Rong Huang, Jamila Asgar, Jing Wang, Ke Ren, Feipeng Zhu, Changhe Wang, Yehua Gan, Feng Wei, Zhuan Zhou

**Affiliations:** ^1^ Central Laboratory Peking University School and Hospital of Stomatology Institute of Molecular Medicine and Peking‐Tsinghua Center for Life Sciences State Key Laboratory of Membrane Biology and Beijing Key Laboratory of Cardiometabolic Molecular Medicine PKU‐IDG/McGovern Institute for Brain Research Peking University Beijing China; ^2^ Department of Neural and Pain Sciences School of Dentistry; Program in Neuroscience Center to Advance Chronic Pain Research University of Maryland Baltimore Maryland USA; ^3^ Peking University Hospital of Stomatology Sanya Division (Sanya Stomatology Center) Sanya China; ^4^ Department of Cardiology Zhongshan Hospital Institutes of Biomedical Sciences Fudan University Shanghai Institute of Cardiovascular Diseases Shanghai China; ^5^ Neuroscience Research Center National Key Laboratory for High Energy Pulsed Power Key Laboratory of Biomedical Information Engineering of the Ministry of Education School of Life Science and Technology Xi'an Jiaotong University Xi'an China; ^6^ Key Laboratory of Medical Electrophysiology Ministry of Education of China Collaborative Innovation Center for Prevention and Treatment of Cardiovascular Disease, and the Institute of Cardiovascular Research Southwest Medical University Luzhou China

**Keywords:** GABA, inflammation, locus coeruleus, mesencephalic trigeminal nucleus, TRPA1

## Abstract

Proprioception, often described as a sixth sense to perceive body position and muscle contraction, has been increasingly implicated in the development and maintenance of chronic pain, yet the underlying mechanisms remain unclear. Here, we identify the functional expression of TRPA1 in proprioceptive mesencephalic trigeminal nucleus (MeV) neurons in a mouse model of orofacial muscle pain. TRPA1 sensitization enhances somatic secretion from MeV neurons, thereby promoting the progression and persistence of inflammatory pain by disinhibiting descending pain control from the neighboring noradrenergic locus coeruleus (LC^NE^) neurons. Local GABAergic neurons that provide input to LC^NE^ neurons serve as a key downstream target of MeV somatic volume transmission, forming a critical relay that transduces signals from the MeV to the LC^NE^ system. Thus, these findings reveal a previously unrecognized somatic volume transmission that links proprioceptive and nociceptive circuits and provides a central mechanism for persistent muscle pain.

## Graphical abstract

Zhang et al. uncover a novel central mechanism for persistent muscle pain, in which TRPA1 sensitization in MeV proprioceptive neurons enhances somatic secretion. This, in turn, disinhibits descending pain control from neighboring noradrenergic locus coeruleus neurons via local GABAergic circuits, thereby promoting inflammatory muscle pain.

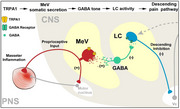

## 1 Introduction

Proprioception (the awareness of posture and movement) and nociception (the sense of harmful stimuli) are fundamental and essential components of sensation across mammalian species. Physiologically, these modalities are mediated by distinct peripheral nerve fibers and central pathways and are traditionally considered to operate independently. However, emerging evidence indicates that they can interact and influence each other. For example, disturbance of proprioception, such as impaired positional sense and muscle tension, has been reported in patients with chronic muscle pain conditions, including myofascial temporomandibular disorders (TMDs), idiopathic low back pain, and fibromyalgia [1, 2, 3], which represent some of the most common and burdensome clinical pain disorder with poorly understood mechanisms [4, 5]. In addition to reducing muscle contraction force, sustained activation of proprioception pathways can exacerbate the development of persistent muscle pain [6, 7, 8] and may even lead to proprioceptive allodynia in certain pathological states [9]. Despite these observations, the neural mechanisms underlying the multifaceted interaction between proprioception and pain and their critical roles in chronic muscle pain remain largely unknown.

Although proprioception and pain are mediated by distinct subpopulations of primary sensory neurons, their neuronal somata for body sensations are clustered in the same dorsal root ganglia (DRG). As a result, it has been difficult to separate these two sensory modalities and to clarify their mutual interaction, which remains a major unresolved issue in this field [10]. In contrast, for the orofacial sensations, the somata are anatomically segregated: primary nociceptive neurons reside in peripheral trigeminal ganglia (TG), whereas primary proprioceptive neurons are located in the mesencephalic trigeminal nucleus (MeV) of the brainstem [11]. The pseudo‐unipolar sensory neurons in the MeV receive proprioceptive input from orofacial structures, including masseter muscles and periodontal ligaments, and relay this information to the trigeminal motor nucleus to convey the feedback control of chewing rhythm and muscle force [3, 12]. Notably, increased MeV neuronal activity has been observed in pain conditions [13, 14]; however, whether, and by what mechanisms, MeV hyperactivity contributes to orofacial muscle pain remains unclear.

Our previous work has demonstrated somatic exocytosis and vesicular release from proprioceptive MeV neurons [15]. Unlike the point‐to‐point communication of synaptic transmission, somatic transmission, as a major form of volume transmission, is temporally slower but enables intercellular chemical signaling across a broader neighborhood without direct anatomical synapses [16, 17, 18, 19, 20], providing a plausible mechanism by which proprioceptive and nociceptive systems could be cross‐linked. However, the lack of tight structure‐connections makes it impossible to definitively assign ‘pre‐’ and ‘post‐’ partners for somatic transmission by current viral or /chemical‐tracing techniques. Whether somatic transmission plays a role in functional coupling between proprioceptive MeV neurons and neighboring nuclei with distinct physiological roles, therefore, remains an open question. Notably, the unique spatial proximity of the MeV and the locus coeruleus (LC) in the brainstem offers an ideal anatomical model to evaluate whether proprioceptive neurons can influence pain‐modulating neurons.

Transient receptor potential (TRP) channels in primary sensory afferents act as cellular sensors and signal integrators. Among them, TRP ankyrin 1 (TRPA1) plays a critical role in both thermo‐ and mechano‐nociception and is strongly implicated in the peripheral mechanism underlying inflammatory and neuropathic pain [21, 22, 23, 24, 25], making it an attractive therapeutic target. In contrast to other channels such as TRP vanilloid 1, P2X purinoceptor3 and acid‐sensing ion channel 3, which are predominantly expressed in nociceptive DRG and TG neurons as transducers for noxious stimulation [26], TRPA1 is also expressed in the peripheral non‐nociceptive neurons [27, 28], including the proprioceptive neurons in Drosophila [29]. Whether TRPA1 is expressed and functionally active in mammalian proprioceptive MeV neurons in physiological and pathological conditions, however, remains unknown.

Here, taking advantage of the single sensory modality and anatomical segregation of MeV neurons in the central nervous system (CNS), we investigated how proprioceptive neuronal activity in the MeV is altered by orofacial muscle inflammation and how these changes affect muscle pain. Using a combination of tracing‐coupled Ca^2+^ imaging, optogenetic stimulation, electrophysiological recordings, single‐cell PCR, in vivo genetic manipulation, and behavioral assays, we identified functional TRPA1 expression in MeV proprioceptive neurons, and further delineated TRPA1‐dependent somatic secretory mechanisms in MeV neurons that enhances the activity of nearby GABAergic neurons, thereby relaying signals from MeV neurons to LC neurons and providing a central mechanism for the persistence of orofacial muscle pain.

## 2 Results

### 2.1 The Functional Expression of TRPA1 in Proprioceptive MeV Neurons

**FIGURE 1:**
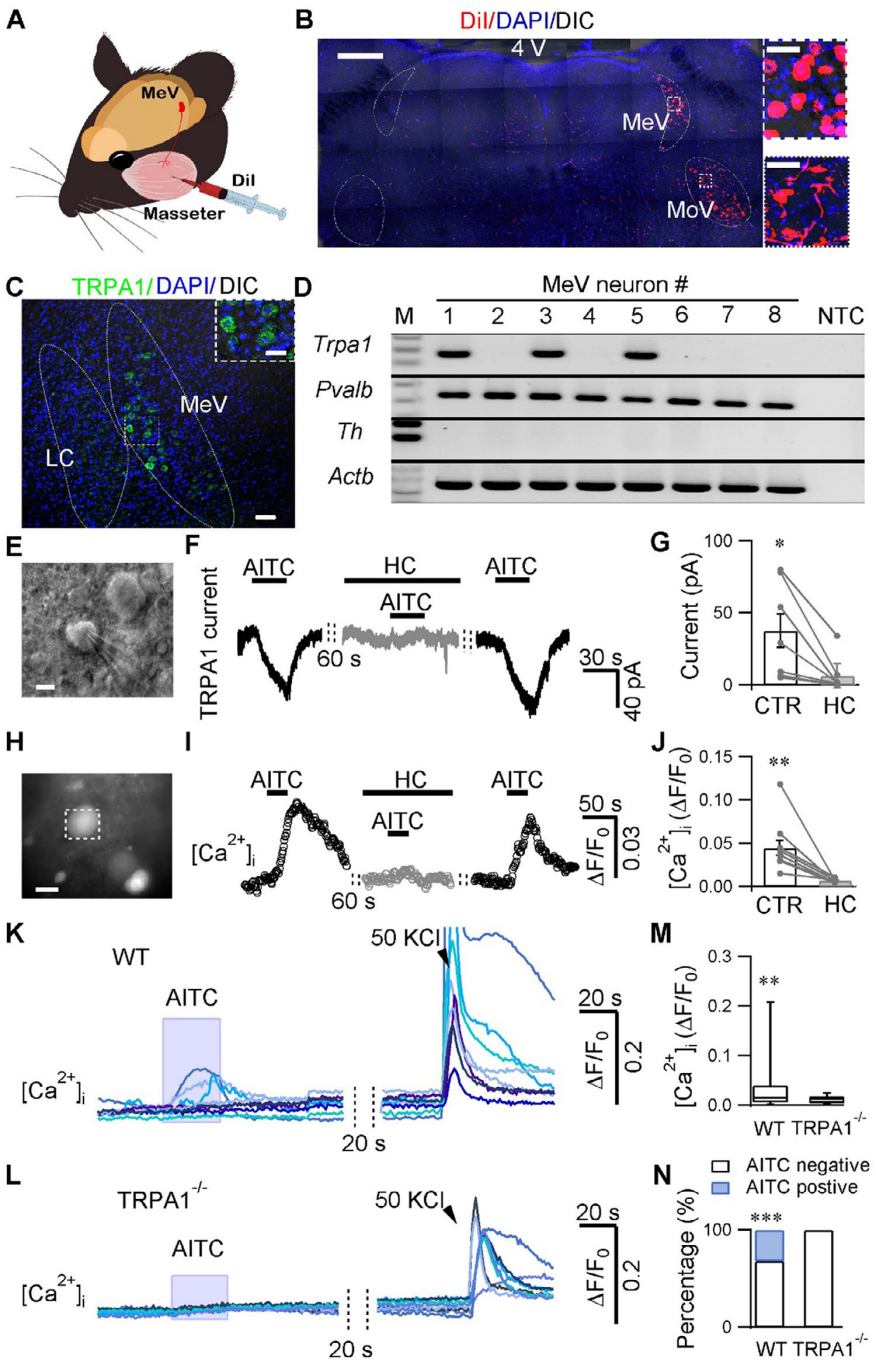
The functional expression of TRPA1 in proprioceptive MeV neurons. (A) Schematic illustration of retrograde labeling of MeV neurons. (B) Micrograph showing labeled MeV neurons after intra‐masseter DiI injection. MoV: trigeminal motor nucleus. Scale bars, 500 µm; 50 µm for enlarged inset. (C) TRPA1‐expressing neurons in a MeV section from an adult mouse. Scale bars, 100 µm; 50 µm for inset. (D) Single‐cell PCR of mRNA expression in MeV neurons; M, 100‐bp DNA marker; NTC, no template control; 1–8, numbers of MeV neurons. (E) Differential interference contrast image of patch‐clamp recording from an MeV neuron. (F, G) Representative traces and statistics of AITC (100 µm)‐induced TRPA1 currents and the block effect by HC‐030031 (HC, 50 µm) (*n* = 6 cells). (H) Fluorescence image of Fura 2‐AM‐loaded MeV neurons under 360 nm illumination. Scale bar, 20 µm. (I, J) Representative traces and statistics showing HC‐030031 (50 µm) blocked the AITC‐induced Ca^2+^ rise (*n* = 10 cells). (K, L) Representative traces showing the Ca^2+^ rise induced by AITC and 50 mm KCl in WT (*n* = 53 cells) and changes in TRPA1‐KO (TRPA1^−/−^, *n* = 29 cells) mice. (M) Pooled data for the amplitude of AITC‐induced Ca^2+^ signals in WT and TRPA1‐KO mice. (N) Percentages of AITC‐responsive MeV neurons in WT and TRPA1‐KO mice. Error bars indicate SEM. ^*^ *p* < 0.05, ^**^ *p* < 0.01, ^***^ *p* < 0.001, paired Student's *t*‐test for (G, J), unpaired Student's *t*‐test for (M), Fisher's exact test for (N).

To locate MeV neurons projecting to the masseter, we injected the retrograde tracer 1,1'‐dioctadecyl‐3,3,3',3'‐tetramethylindocarbocyanine perchlorate (DiI) into the masseter muscle of rats and mice and found that approximately 90.7% MeV neurons in rats and 88% in mice were DiI‐labeled (Figure 1A; Figure S1A), consistent with previous reports of 80%–90% [11]. DiI‐labeled neurons were found in the ipsilateral MeV along its entire rostro‐caudal extent, from the lateral margin of the periaqueductal grey at the level of the superior colliculus to the trigeminal motor nucleus in the mouse midbrain (Figure S1A). Double retrograde tracing revealed that individual MeV neurons collateralize to innervate both periodontal ligaments and masseter muscle spindles in mice, and approximately 52% of MeV neurons were labeled from periodontal ligaments (Figure S1A). Most DiI‐labeled neurons were located in the MeV region just lateral to the LC at the level of the genu of the facial nerve in rats (Figure 1B) and mice (Figure S1A). DiI also labeled ipsilateral trigeminal motor neurons and a subset of nerve fibers that may originate from MeV neurons (Figure 1B).

Based on DiI labeling of MeV neurons in brainstem sections, we next examined TRPA1 expression in the somata of proprioceptive neurons. Immunostaining showed abundant TRPA1 expression in MeV neurons but not adjacent nuclei, including the LC, in rats (Figure 1C). Consistently, TRPA1 immunoreactivity was present in MeV neurons from wild‐type (WT) but not TRPA1‐knockout (KO) mice (Figure S1B), confirming the specificity of the anti‐TRPA1 antibody [30] and TRPA1 expression in MeV neurons in mice. TRPA1 was also detected in human MeV neurons (Figure S1C), indicating the conserved TRPA1 expression in MeV neurons across species. To further validate TRPA1 expression in proprioceptive MeV neurons, we performed real‐time PCR on single DiI‐labeled cells from adult mice brainstem slices and found that all TRPA1 mRNA‐containing neurons co‐expressed parvalbumin (PV) mRNA (Figure 1D), a well‐established marker of primary proprioceptive neurons [31].

To validate functional TRPA1 expression in MeV neurons, we performed whole‐cell patch‐clamp recordings and Ca^2+^ imaging in brainstem slices from 7‐day‐old (P7) rats [30] (Figure 1E). As expected, allyl isothiocyanate (AITC, 100 µm), a selective TRPA1 agonist, elicited an inward membrane current in approximately one fourth of MeV neurons (16/61 cells, Figure 1F), suggesting the presence of functional TRPA1 channels on the plasma membrane. Since TRPA1 is a Ca^2+^‐permeable channel, we then performed Ca^2+^ imaging in rat brainstem slices and found that AITC (100 µm) induced a transient rise in intracellular Ca^2+^ ([Ca^2+^]_i_) in MeV neurons (Figure 1H,I), which was diminished in Ca^2+^‐free solution (Figure S2A,B). Similarly, the endogenous TRPA1 agonist 4‐hydroxynonenal (4‐HNE, 30 µm) or 4‐hydroxyhexenal (4‐HHE,10 µm) also induced Ca^2+^ rise in MeV neurons (Figure S2C). Together, these data indicate that TRPA1 is expressed mainly on the plasma membrane of MeV neurons.

Next, we used TRPA1 antagonists and neonatal TRPA1‐KO mice of either sex to further confirm the specificity of the AITC‐induced responses. The TRPA1‐specific antagonist HC‐030031 blocked the AITC‐induced inward current (Figure 1F,G). Consistently, both the non‐specific TRPA1 blocker ruthenium red (30 µm) (Figure S2E,F) and specific antagonist HC‐030031 (50 µm) (Figure 1I,J) abolished the AITC‐evoked Ca^2+^ signals. Moreover, AITC failed to induce a detectable [Ca^2+^]_i_ rise (0/29 cells) in MeV neurons from TRPA1‐KO mice, although these neurons responded to high K^+^ (50 mm), whereas in WT mice, AITC induced Ca^2+^ signals in ∼28% of MeV neurons (15/53 cells) (Figure 1K,L). The average amplitude of AITC‐evoked Ca^2+^ response was larger than in TRPA1‐KO neurons (Figure 1M). Fisher's exact test confirmed that the proportion of AITC‐responsive MeV neurons differed between genotypes (Figure 1N). Collectively, these findings provide strong evidence for functional TRPA1 expression in proprioceptive MeV neurons.

### 2.2 TRPA1 Upregulation and Neuronal Sensitization in the MeV After Masseter Inflammation

**FIGURE 2:**
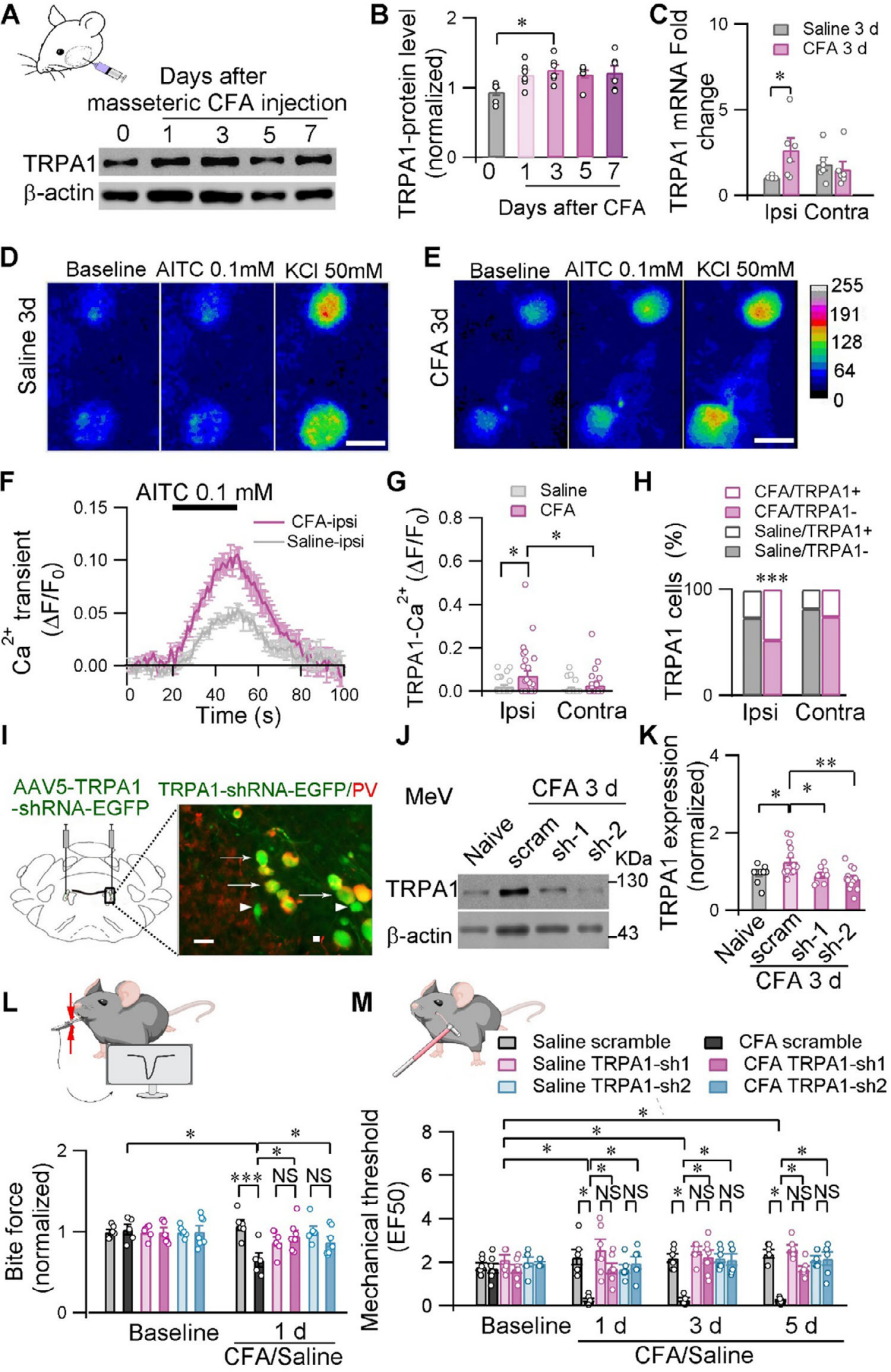
TRPA1 sensitization in MeV neurons mediates masseter inflammation‐induced mechanical hyperalgesia. (A) Upper: schematic illustration of an inflammatory model induced by CFA injection into the masseter. Lower: Western blots showing time course‐dependence of the inflammation‐induced TRPA1 expression in MeV neurons. (B) Pooled data and statistics for gels as in (A) (*n* = 6 mice/group). (C) TRPA1 mRNA level in ipsilateral (Ipsi), but not contralateral (Contra) MeV tissue 3 days after intra‐masseter injection of CFA versus saline control (*n* = 6 mice/group). (D, E) Representative Ca^2+^ imaging of ipsilateral MeV neurons in GCaMP5 mice at 3 days after CFA (D) or saline (E) injection. Scale bars, 20 µm. (F) Averaged time courses of [Ca^2+^]_i_ signals in response to 100 µm AITC (F, *n* = 5 cells) from experiments as in (D, E). (G) Statistics of peaks [Ca^2+^]_i_ induced by AITC (Ipsi, *n* = 29 cells; Contra, *n* = 24 cells) in ipsilateral sides, as in (D–F), with separate contralateral sides. (H) Percentages of AITC‐sensitive neurons in the MeV at day 3 after CFA or saline injection (*n* = 24–29 cells from 5 mice). (I) Schematic illustration of virus microinjection and labeling of MeV neurons by TRPA1‐shRNA‐EGFP (arrow and arrowhead indicated), and immunostaining of PV in infected MeV neurons (arrow indicated; Scale bars, 100 µm for left, 20 µm for right). (J, K) Representative Western blots and statistics of knockdown of TRPA1 protein by two shRNAs in the ipsilateral MeV at day 3 after CFA injection. Scrambled shRNA (scrambled) served as control (*n* = 10 mice for naïve, 15 for scrambled, 8 for sh‐1, and 12 for sh‐2). (L) Upper: schematic illustration of bite force measurement in mice. Lower: bite force changes in CFA‐inflamed (*n* = 6–7 mice/group) or saline‐control (*n* = 5–6 mice/group) mice with TRPA1‐KD (sh1 or sh2) in the MeV at day 1 after CFA‐injection. Baseline was detected at 4 weeks after virus injection. (M) upper: schematic illustration of mechanical head‐withdrawal threshold using von Frey filament in mice. Lower: mechanical hyperalgesia in inflamed and saline‐treated mice as in (L) (*n* = 5–7 mice/group). Baseline was detected at 4 weeks after virus injection. Error bars indicate SEM. ^*^ *p* < 0.05, ^***^ *p* < 0.001, NS, no significance. One‐way ANOVA for (B), two‐way ANOVA for (C, G), Fisher's exact test for (H), two‐way ANOVA for (K), three‐way ANOVA for (L, M).

TRPA1 channels play a key role in nociception in primary sensory neurons and contribute to the development of inflammatory pain [32], but whether they modulate MeV proprioceptive neuronal activity in response to muscle injury remains unclear. To address this question, we adopted a mouse model of orofacial muscle pain in which complete Freund's adjuvant (CFA) was injected into the unilateral masseter muscle (Figure 2A) [33]. Western blot analysis showed a time course‐dependent upregulation of TRPA1 protein in the ipsilateral MeV in adult male mice during the first 7 days after CFA injection, with a peak on day 3, relative to naïve control (Figure 2A,B). Consistently, real‐time PCR revealed a two‐fold increase of TRPA1 transcription in the ipsilateral but not the contralateral MeV region on day 3 after CFA injection compared with saline‐injected controls (Figure 2C). Next, we assessed MeV neuronal activity in brainstem slices from adult male GCaMP5 transgenic mice (Figure S3) [34] at day 3 after CFA (Figure 2D) or saline (Figure 2E) injection. In line with the elevated TRPA1 expression, AITC dose‐dependently induced Ca^2+^ influx in MeV slices (Figure S4), and importantly, the AITC (100 µm)‐induced Ca^2+^ signals in the ipsilateral MeV neurons from inflamed mice were much higher than from the saline group (Figure 2F,G). As a control, the 50‐mm KCl depolarization‐induced Ca^2+^ signals were comparable across groups (Figure S5). The proportion of AITC‐responsive neurons was also increased after CFA injection (Figure 2H). Together, these findings indicate the sustained regulation and sensitization of TRPA1 in MeV neurons after masseter inflammation.

### 2.3 TRPA1 knockdown in the MeV Restores Inflammation‐induced Early Bite‐force Reduction and Persistent Hyperalgesia

Masseter inflammation induces long‐lasting mechanical hypersensitivity and bite‐force reduction (myasthenia) [35, 36]. To determine whether TRPA1‐upregulation in MeV neurons contributes to these pain behaviors, we injected adeno‐associated virus serotype 5 (AAV5) carrying TRPA1‐shRNAs (EGFP) into the MeV to knock down (KD) TRPA1 expression in adult male mice (Figure 2I). Immunostaining confirmed viral infection of PV‐positive MeV neurons 4 weeks after viral injection (Figure 2I), and Western blot analysis showed efficient KD of TRPA1 by two independent shRNAs (sh‐1 and sh‐2 targeting different sequences of TRPA1) in MeV tissue from naïve mice (Figure S6A,B). In inflamed mice, both shRNAs completely abolished the CFA‐induced upregulation of TRPA1 in the MeV (Figure 2J,K).

Bite force measurement, a key indicator of the masticatory function and muscle pain, showed that the inflamed mice displayed a marked reduction in bite force on day 1 after CFA injection (Figure S7A,B), consistent with early inflammatory muscle hyperalgesia in rats [37] and mice [35]. Strikingly, TRPA1 KD with either shRNA largely reversed this inflammation‐induced reduction in bite force at day 1, whereas scramble shRNA had no effect (Figure 2L; Figure S7A), and TRPA1 KD did not alter bite force in naïve or saline‐injected adult male mice (Figure S7C). Importantly, TRPA1 KD completely prevented the mechanical hyperalgesia induced by masseter inflammation when compared with scrambled shRNA, while having no effect on the basal mechanical threshold in naïve or saline‐injected mice (Figure 2M). Moreover, all TRPA1‐KD mice showed normal motor activity in the rotarod test when compared with scrambled controls (Figure S7D), suggesting that intra‐MeV TRPA1‐KD selectively reduced inflammatory muscle pain without impairing motor coordination. Together, these results provide direct evidence that TRPA1 upregulation and sensitization in proprioceptive MeV neurons after masseter inflammation are implicated in the development of inflammatory muscle pain.

### 2.4 MeV Neuronal Exocytosis Contributes to Inflammatory Muscle Pain

**FIGURE 3:**
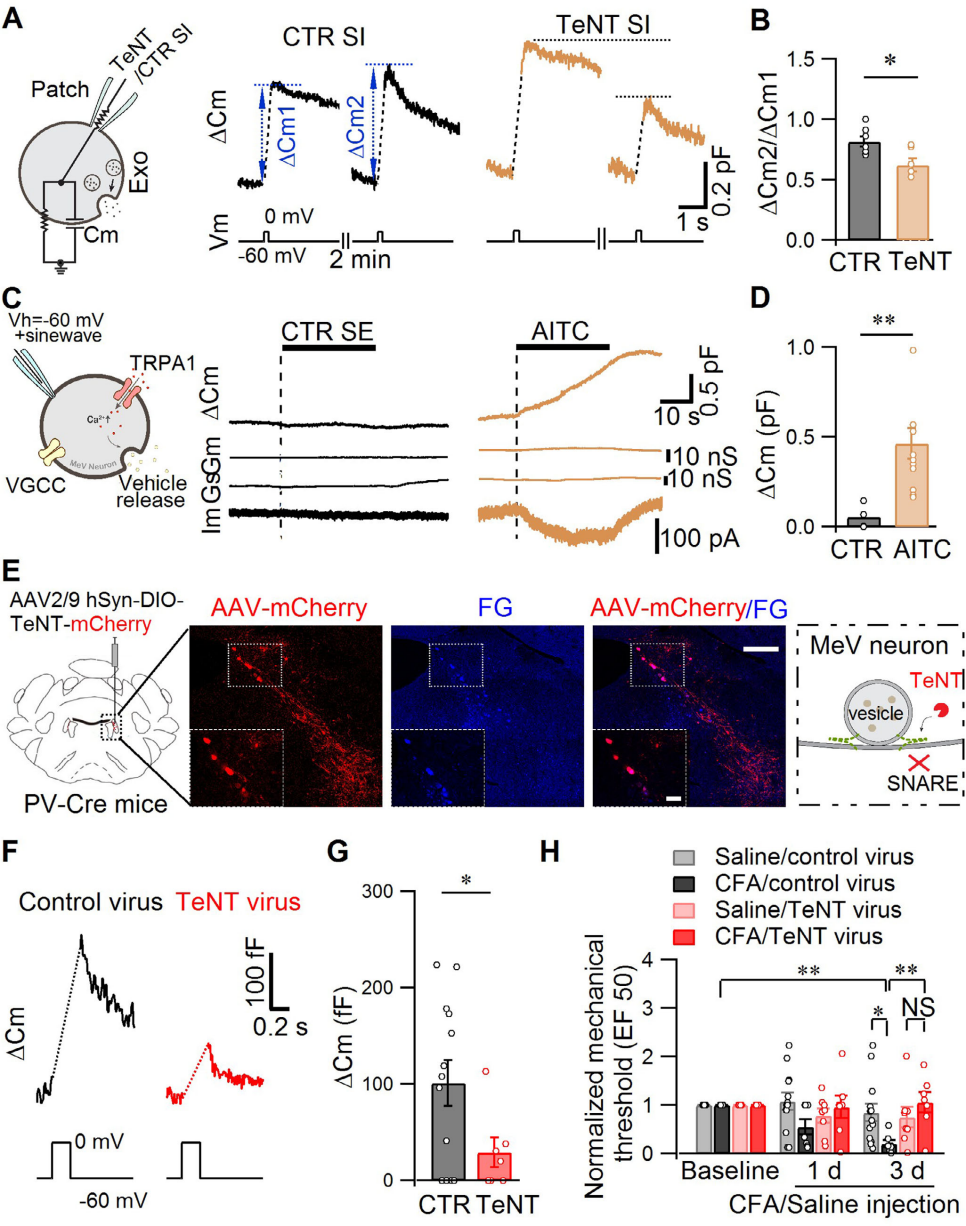
SNARE‐dependent vesicular exocytosis of MeV neurons mediates CFA‐induced mechanical hyperalgesia. (A, B) Left: schematic illustration of cellular capacitance measurement (Cm) with the patch‐clamp technique to examine vesicular exocytosis (Exo). Right: representative *C*_m_ traces (exocytosis), and statistics of C_m_ jumps after whole‐cell dialysis of TeNT (2 µm, 5 min) and control SI (CTR SI) in MeV somata (*n* = 7 cells for CTR and 5 for TeNT). (C) Left: cartoon showing *C*_m_ recording of MeV neurons in response to TRPA1 activation in brain slices. Right: representative exocytosis (*C*_m_) and membrane current (*I*_m_) traces showing that application of 100 µm AITC (right), but not the normal SE (left), triggered a *C*_m_ increase along with a TRPA1 current, with membrane conductance (*G*_m_) and series conductance (*G*_s_) served as controls for recording. (D) Statistics of *C*_m_ jumps in (C) (*n* = 4 for SE and 11 for AITC). (E): Left: infection of MeV neurons by Cre‐dependent AAV2/9 virus expressing tetanus toxin (TeNT)/mCherry, and its retrograde labeling by Fluoro‐Gold (FG) injected from the masseter in brain slices in PV‐Cre mice. Scale bar: 200 µm, Scale bar for inset: 50 µm. Right: schematic illustration of the effect of TeNT on the SNARE complex during vesicular release. (F, G) Representative traces (F) and statistics (G) showing depolarization‐induced cellular capacitance signals in MeV neurons after Cre‐dependent TeNT virus (DIO‐TeNT‐mCherry) and control virus (DIO‐mCherry) in PV‐Cre mice. *n* = 13 cells for control and *n* = 7 cells for TeNT virus. (H) Cre‐dependent TeNT virus attenuated CFA‐induced mechanical hyperalgesia in PV‐Cre mice. *n* = 7–13 mice/group. Baseline of mechanical threshold via von Frey test was examined at 4 weeks after TeNT or control virus injection in one side of the MeV nucleus in PV‐Cre male mice. Error bars indicate SEM. ^*^ *p* < 0.05, ^**^ *p* < 0.01, ^***^ *p* < 0.001, NS, no significance. Unpaired Student's *t*‐test for (B, D, and G), and three‐way ANOVA for (H).

To investigate how TRPA1 sensitization and exocytosis contribute to neuronal signaling transduction, we next examined the cellular mechanisms underlying MeV somatic exocytosis. Ca^2+^‐dependent somatic exocytosis was assessed with patch clamp membrane capacitance (*C*_m_) recordings in neonatal rat brain slices as previously reported [15, 18, 19, 38]. We first investigated the vesicular release properties of MeV neurons by using whole‐cell dialysis of tetanus toxin (TeNT, which cleaves vesicle‐associated membrane protein 2, VAMP2) into patched cells [18, 39] (Figure 3A). After treatment with 2 µm TeNT for 5 min, depolarization‐induced *C*_m_ jump was reduced by ∼24%, whereas no significant change was observed under control conditions (Figure 3A,B), suggesting the soluble N‐ethylmaleimide‐sensitive factor attachment protein receptor (SNARE)‐dependent vesicular secretion from MeV neurons. Consistently, dynasore, a potent and specific blocker of dynamin, markedly slowed the rate of *C*_m_ decay after exocytosis (Figure S8A,B), indicating dynamin‐dependent endocytosis following exocytosis and further supporting the vesicular exocytosis and endocytosis of the somata of MeV neurons. In addition, depolarization‐induced somatic exocytosis was also detected in MeV neurons from brain slices of adult male mice (Figure S9A).

Given that TRPA1 is a non‐selective cation channel that is highly permeable to Ca^2+^ (fractional Ca^2+^ current of ∼17% [40], about 2.5 fold that of NMDA receptors) [41], we next tested whether TRPA1‐mediated Ca^2+^ rise can trigger somatic secretion in MeV neurons using *C*_m_ recordings [30]. Extracellular application of AITC (100 µm, 30 s) induced a gradual exocytotic *C*_m_ increase with normal SE (aCSF) solution serving as a control (Figure 3C,D). Consistent with the Ca^2+^‐dependence of vesicular exocytosis, this *C*_m_ increase was completely blocked in Ca^2+^‐free solution in adult male mice (Figure S9B,C). We then performed *C_m_* recording in adult TRPA1 knockout mice of either sex and found that AITC failed to evoke both TRPA1 currents and Cm increase (Figure S10), indicating that the AITC‐induced Cm increase is specifically mediated by TRPA1 channels. These results suggest that TRPA1 activation‐induced somatic secretion from the MeV neurons can enable communication with neighboring neurons and modulate their activity.

To study whether vesicular secretion from MeV neurons contributes to masseter muscle pain, we stereotaxically injected a Cre‐dependent AAV2/9 expressing TeNT‐A2A‐mCherry into the MeV nucleus of adult male PV‐Cre mice to specifically block the SNARE‐dependent vesicular exocytosis in proprioceptive MeV neurons. Immunostaining showed co‐localization of mCherry with the retrograde tracer Fluoro‐Gold (FG) in the MeV 7–10 days post‐masseteric FG injection (4%, 20 µL, 4 weeks post‐virus injection, Figure 3E), confirming restricted TeNT expression in proprioceptive MeV neurons. *C*_m_ recording from mCherry‐positive MeV neurons showed that the depolarization‐induced exocytosis (*C*_m_ jump) was reduced by ∼70% in TeNT virus‐infected neurons compared with control virus (Figure 3F,G). Importantly, mechanical hyperalgesia was significantly attenuated by TeNT virus at day 3 after CFA injection into the masseter (Figure 3H), demonstrating a key role of MeV neuronal exocytosis in the development of orofacial muscle pain.

### 2.5 TRPA1 Activation in MeV Contributes to Inflammatory Pain

To determine whether TRPA1 activation in the MeV is sufficient to induce inflammatory pain, we microinjected TRPA1 agonist AITC into the MeV nucleus via an implanted cannula and measured mechanical nociceptive threshold in masseteric region in naïve adult male mice, a AITC administration robustly induced mechanical hyperalgesia in the masseter region (Figure S11), supporting a direct pro‐nociceptive role of TRPA signaling in this circuit. To further investigate the SNARE dependence of TRPA1‐mediated mechanical hyperalgesia, mice received intra‐MeV microinjections of either Cre‐dependent AAV‐TeNT‐mCherry or control AAV‐mCherry (Figure S12A) before induction of inflammation. Subsequent microinjection of AITC into the MeV failed to trigger inflammatory hyperalgesia in the TeNT‐treated group (Figure S12B,C), in contrast to the robust facilitatory effect observed in naïve mice (Figure S11). These findings suggest that the pro‐nociceptive effect of TRPA1 activation in MeV neurons requires SNARE‐dependent vesicular release. Notably, AITC injection produced only a marginal effect in the control virus group under inflammatory conditions (Figure S12C). This lack of further facilitation is likely due to a floor effect, whereby the markedly reduced mechanical threshold during inflammation leaves little dynamic range for additional TRPA1‐mediated sensitization, or alternatively, to saturation of TRPA1 function under inflammatory conditions.

### 2.6 MeV‐LC Cross‐Talk Underlies Orofacial Inflammatory Pain

**FIGURE 4:**
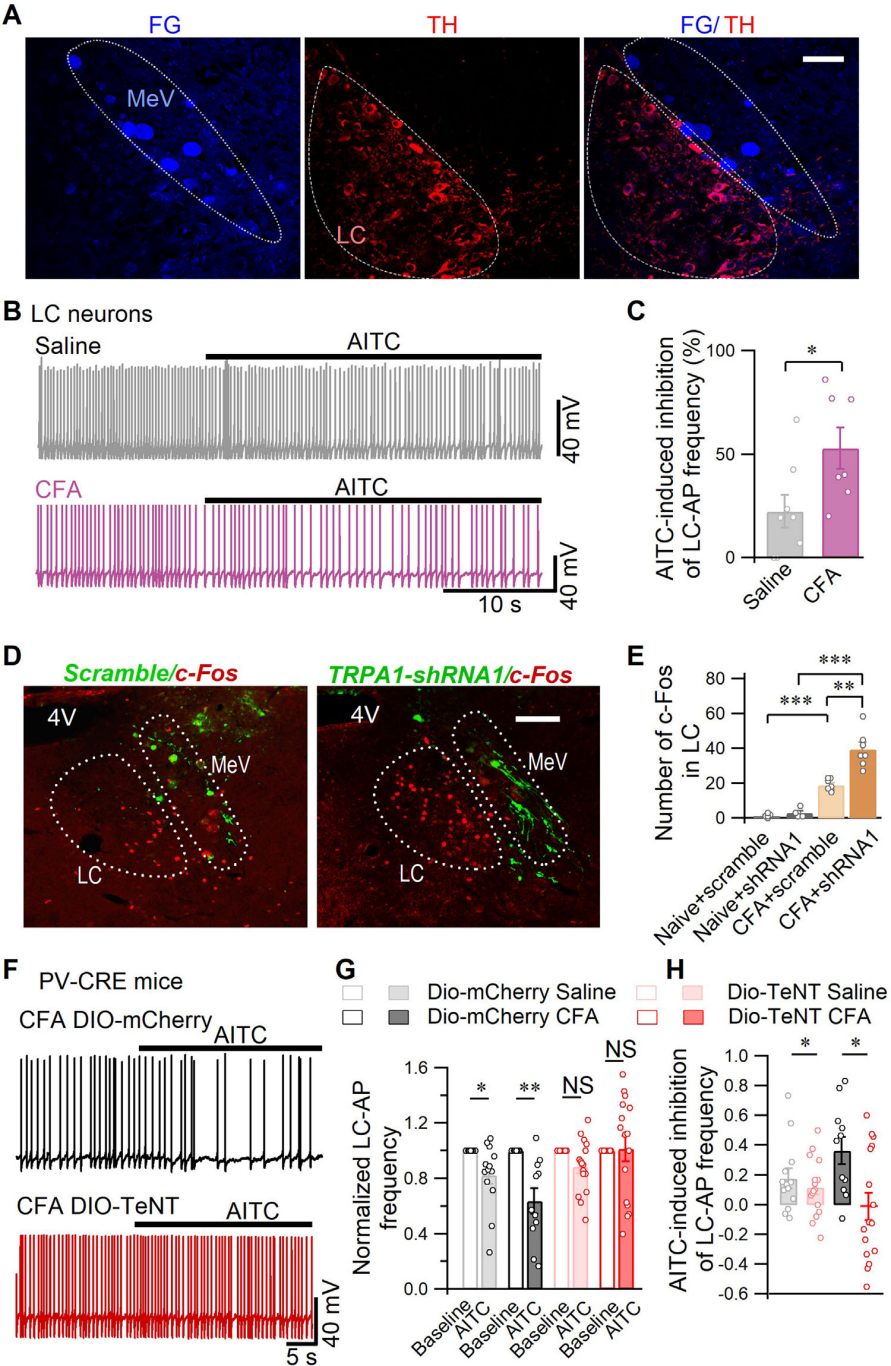
TRPA1 activation of MeV neurons inhibits LC neuronal activity. (A) Immunofluorescence showing the neighboring location of Fluoro‐Gold (FG) labeling MeV neurons (retrograde labeling from masseteric injection) and TH‐positive LC neurons in brain slices. Scale bar, 50 µm. (B) Representative traces showing the inhibitory effect of AITC (100 µm) on the firing frequency of LC neurons in an inflamed and a saline control mouse. (C) Statistics of action potential as in (B) (*n* = 7–8 cells from 5 mice/group). (D) Number of c‐Fos‐positive neurons in the ipsilateral LC in inflamed mice with MeV AAV5‐TRPA1‐shRNA1‐EGFP and AAV5‐scrambled shRNA‐EGFP (scramble) injection in MeV nucleus at day 3 after intra‐masseter CFA injection. (E) Quantification of c‐Fos‐positive neurons in the ipsilateral LC after TRPA1‐shRNA and scrambled shRNA injection as in (D) in naïve and inflamed mice (*n* = 5–7 mice/group). (F–H) Representative traces and statistics showing LC neuronal activity at 4 weeks after TeNT or control virus infection of MeV neurons in PV‐Cre mice at day 3–5 after CFA or saline intra‐masseter injection (*n* = 11–16 neurons from 3–4 mice/group). Error bars indicate SEM. ^*^ *p* < 0.05, ^**^ *p* < 0.01, ^***^ *p* < 0.001, NS, no significance. Unpaired Student's *t*‐test for (C), paired Student's *t*‐test for (G), two‐way ANOVA for (E and H).

To investigate how TRPA1 sensitization and exocytosis in MeV neurons are involved in the central mechanisms of inflammatory masseter muscle pain, we considered the pseudo‐unipolar property of proprioceptive MeV neurons and hypothesized that the somatic secretion and resulting volume transmission to neighboring cells play a critical role. MeV neurons were retrogradely labeled with FG (4%, 20 µL) at 7–10 days after masseteric injection in adult male mice. Tyrosine hydroxylase (TH) immunostaining showed that FG‐labeled MeV neurons are located in close proximity to LC noradrenergic (LC^NE^) neurons (Figure 4A), which are key mediators of descending pain inhibition [20, 42, 43]. This observation led us to hypothesize that MeV‐TRPA1 signaling may influence masseter muscle pain by modulating LC neuronal activity. Because both immunostaining and single‐cell PCR confirmed the absence of TRPA1 expression in LC^NE^ neurons (Figure 1C; Figure S13), we applied AITC (100 µm) locally to the MeV area in brainstem slices from adult mice 3 days after CFA or saline injection to test whether TRPA1‐activation in MeV neurons alters LC neuronal functions. AITC application substantially reduced the spontaneous firing of LC^NE^ neurons, and this inhibitory effect was further enhanced in inflamed mice (Figure 4B,C), suggesting that TRPA1‐dependent somatic secretion from proprioceptive MeV neurons increases inhibitory drive onto neighboring LC^NE^ neurons.

Because intra‐MeV TRPA1‐shRNAs attenuated the mechanical hyperalgesia, we next tested whether neuronal activity of LC^NE^ neurons was affected correspondingly in adult male mice. The effects of intra‐MeV TRPA1‐shRNAs on LC neuronal activity were validated by using c‐Fos immunostaining. As expected, we observed the increased number of c‐Fos‐expressing neurons in the ipsilateral LC 3 days after CFA injection (Figure S14). Strikingly, TRPA1‐KD in the MeV further enhanced c‐Fos expression in the LC compared with scrambled shRNA controls in inflamed animals, whereas TRPA1‐KD did not affect the basal c‐Fos expression in naïve mice (Figure 4D,E), supporting the view that LC neurons act downstream in the MeV‐TRPA1 signaling in pain modulation.

To directly test whether TRPA1‐mediated somatic secretion from MeV neurons modulates LC activity, we injected a Cre‐dependent TeNT/mCherry AAV into the MeV of adult male PV‐Cre mice at 4 weeks before CFA or saline injection, and performed electrophysiological recordings in brain slices at day 3 after masseteric injection (Figure 4F). LC^NE^ neurons were identified by their characteristic morphology and regular firing pattern as previously described [20, 44]. Strikingly, AITC‐induced reduction of spontaneous firing in LC neurons was completely abolished in mice expressing TeNT in MeV neurons in both CFA and saline groups at day 3–5 after injection (Figure 4G), and the AITC‐mediated inhibitory effect was significantly abolished by TeNT virus compared with control virus under both conditions (Figure 4H). These findings demonstrate a critical role for TRPA1‐dependent MeV neuronal secretion in the inhibition of LC^NE^ neurons.

**FIGURE 5:**
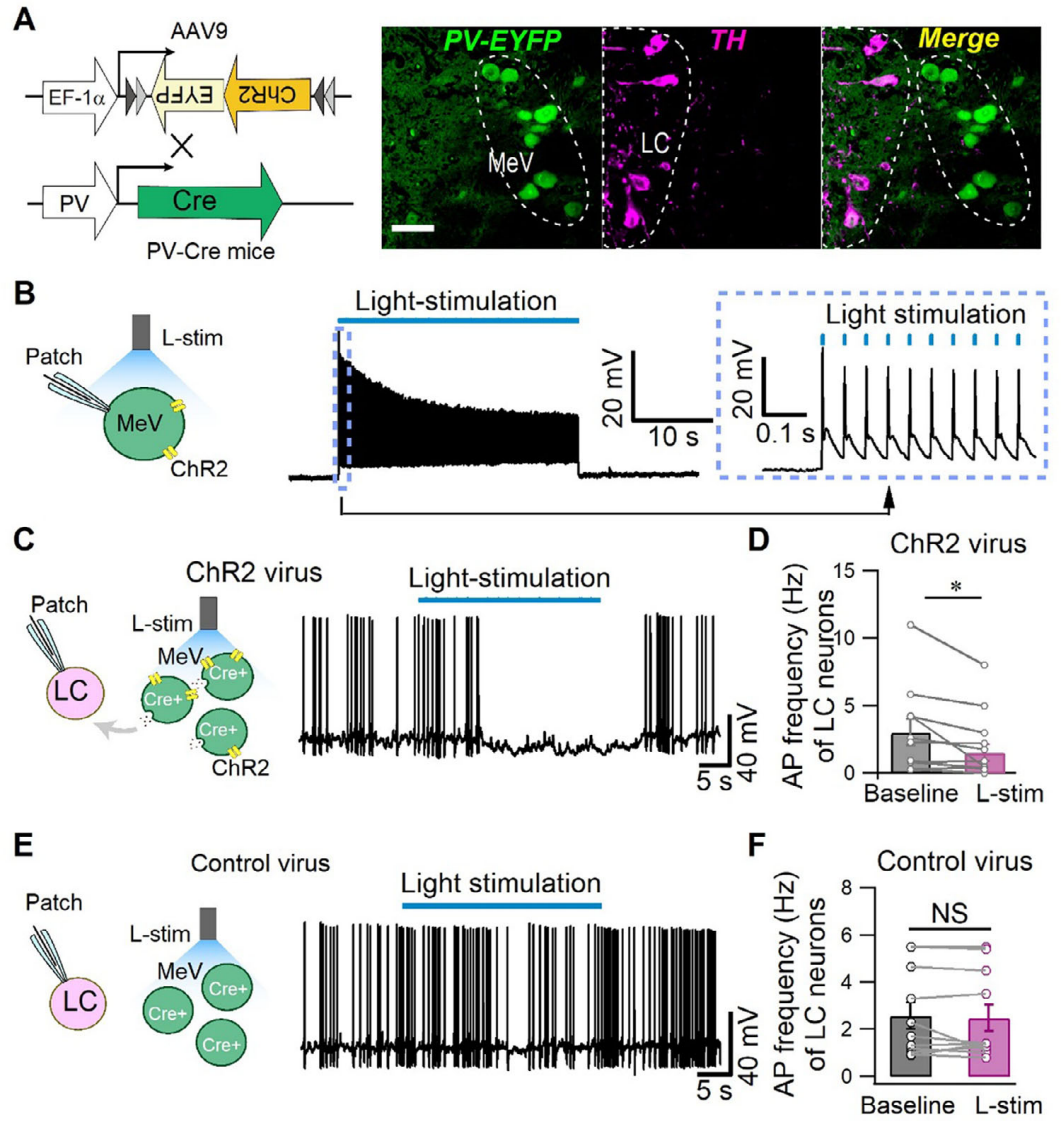
Optogenetic activation of MeV neurons inhibits LC neuronal activity (A) Left: schematic showing microinjection of Cre‐dependent AAV9‐EYFP‐ChR2 in PV‐Cre mice (left panel). Right: representative micrograph showing MeV neurons were infected by Cre‐dependent AAV9 and expressed EYFP, and TH‐positive LC neurons were located next to the MeV nucleus in a brain stem section of a PV‐Cre mouse. Scale bar, 50 µm. (B) Left: schematic showing patch clamp recording of ChR2‐expressing MeV neurons in response to light stimulation. Middle: representative traces showing action potentials in ChR2‐expressing MeV neurons (middle) in response to blue light pulse stimulation (20 Hz, 600 pulses, 30 s, 2 ms per pulse). Right: enlargement of the action potential in the green box area of the middle panel. (C) Left: schematic illustration of LC neuronal recording in response to optogenetic stimulation. ChR2 was expressed in MeV neurons in PV‐Cre mice in brain slices. Right: representative traces showing LC neuron activity in response to blue light pulse stimulation (20 Hz, 600 pulses, 30 s, 5 ms per pulse). (D) statistics of (C) (*n* = 11 neurons). (E) Left: schematic illustration of LC neuronal recording in response to optogenetic stimulation in brain slices. Control virus (DIO‐mCherry) was expressed in MeV neurons in PV‐Cre mice. Right: representative traces showing LC neuronal activity in response to blue light pulses (20 Hz, 600 pulses, 30 s, 5 ms per pulse). (F) Statistics of (E) (*n* = 11 neurons). Error bars indicate SEM. NS, no significance. ^*^ *p* < 0.05, paired Student's *t*‐test.

To further substantiate the inhibitory influence of MeV neuronal secretion on LC neurons, we used an optogenetic approach to selectively activate MeV neurons. An AAV9 expressing Cre‐dependent channelrhodopsin‐2 (ChR2)‐EYFP was microinjected into the MeV of PV‐Cre adult mice of either sex [45]. EYFP‐positive neurons were confined to the MeV and were not detected in the adjacent LC^NE^ neurons (Figure 5A). Combining patch‐clamp recordings with optogenetic stimulation, we found that blue light (470 nm) stimulation (L‐stim) reliably drove firing in ChR2‐expressing MeV neurons in brainstem slices (Figure 5B). Notably, L‐stim also caused a gradual decrease in spontaneous firing of neighboring LC^NE^ neurons (Figure 5C,D), an effect absent in control virus‐infected mice (Figure 5E), further confirming that selective excitation of MeV neurons leads to the progressive inhibition of adjacent LC^NE^ neurons.

### 2.7 LC^NE^ Neuronal Activity Contributes to Orofacial Inflammatory Pain

**FIGURE 6:**
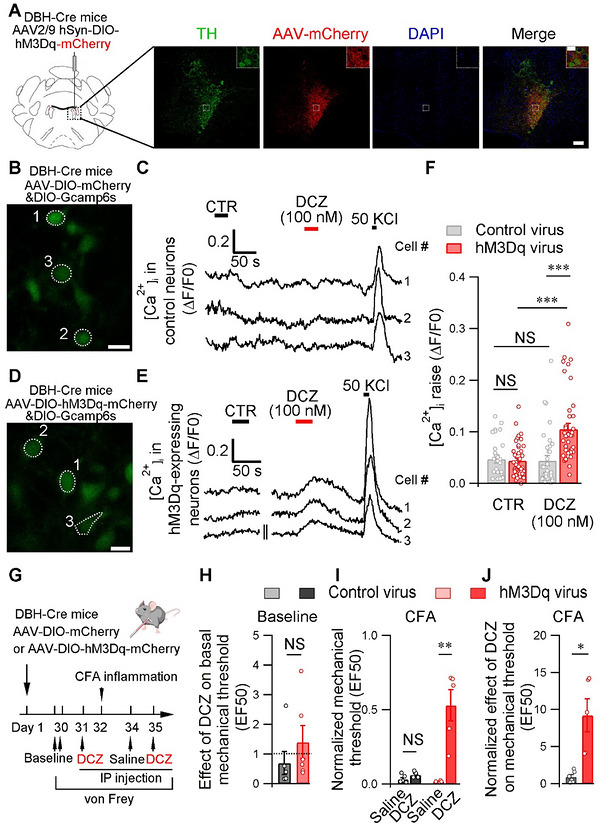
Effect of chemogenetic activation of LC neurons on mechanical threshold. (A) Schematic illustration of virus microinjection and labeling of LC neurons with DIO‐hM3Dq‐mCherry in DBH‐Cre mice, and immunostaining of TH in infected LC neurons (arrows indicated; scale bars, 100 µm; 20 µm for enlarged inset). (B–E) Fluorescence images and representative [Ca^2+^]_i_ signals induced by the DREADD agonist DCZ (100 nm) in Cre‐dependent AAV2/9‐GCaMP6s‐labeled LC neurons under 488 nm illumination in DBH‐Cre mice co‐injected with either Cre‐dependent AAV2/9‐hM3Dq‐mCherry (B, C) or control AAV2/9‐mCherry (D, E). Normal extracellular solution (control, CTR) and high K^+^ (50 mm) were used as negative and positive controls, respectively; scale bar, 20 µm. (F) Statistics of amplitude of [Ca^2+^]_i_ signals as shown in (C, E). *n* = 35–42 neurons per group. (G) Schematic showing mechanical head‐withdrawal threshold measurement using von Frey filaments after i.p. injection of DCZ (0.1 mg/kg) or saline in DBH‐Cre mice, at 4 weeks after intra‐LC injection of Cre‐dependent AAV2/9‐hM3Dq‐mCherry or control AAV2/9‐mCherry in the LC nucleus with or without CFA‐induced inflammation. (H–J) Effect of chemogenetic activation on mechanical threshold in naïve (H) and CFA‐inflamed DBH‐Cre mice (I, J) (*n* = 5–6 mice per group). Error bars indicate SEM. ^*^ *p* < 0.05, ^**^ *p* < 0.01, ^***^ *p* < 0.001, NS, no significance. Two‐way ANOVA for (F), paired Student's *t*‐test for (I), unpaired Student's *t*‐test for (H, J).

To determine how the LC^NE^ neuronal activity affects orofacial inflammatory pain, we injected a Cre‐dependent AAV2/9‐hM3Dq‐mCherry into the LC of adult male dopamine beta‐hydroxylase (DBH)‐Cre mice, and immunostaining confirmed selective expression in LC^NE^ neurons (Figure 6). We then validated the chemogenetic activation in slices by performing Ca^2+^ imaging in LC^NE^ neurons after viral injection of a mixture of Cre‐dependent AAV2/9‐GCaMP6s and AAV2/9‐hM3Dq‐mCherry (or control virus AAV2/9‐mCherry), and found that DREADD agonist deschloroclozapine (DCZ, 0.1 µm) induced a [Ca^2+^]_i_ rise in hM3Dq‐expressing LC^NE^ neurons but not in control neurons (Figure 6B–F). We next performed von Frey testing in DBH‐Cre mice at 4 weeks after viral injection of AAV2/9‐hM3Dq‐mCherry (or control virus AAV2/9‐mCherry). Chemogenetic activation of LC neurons (DCZ, 0.1 mg/kg, i.p.) exhibited a marginal effect on mechanical sensitivity in naïve mice, but markedly attenuated mechanical hyperalgesia on day 3 after masseteric CFA injection. In contrast, DCZ had no effect on mechanical hyperalgesia in control mice expressing AAV2/9‐mCherry (Figure 6G–J). These results indicate that selective activation of LC^NE^ neurons alleviates inflammation‐induced mechanical hyperalgesia.

### 2.8 MeV‐LC^NE^ Transmission via Local GABAergic Neurons

**FIGURE 7:**
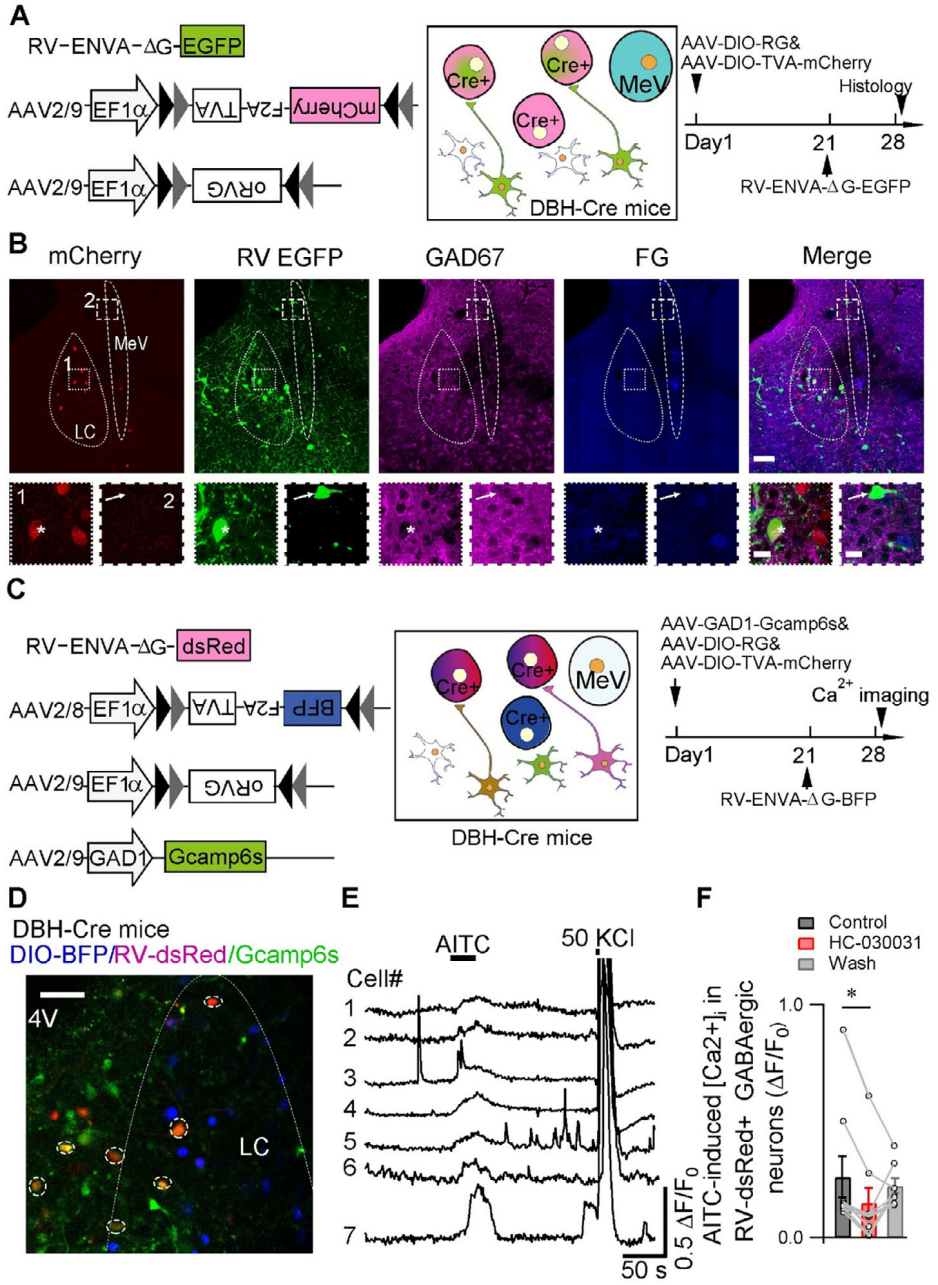
TRPA1 activation in MeV neurons inhibits LC^NE^ neurons via local GABA tone. (A) Schematic illustration of retrograde labeling of GABAergic neurons projecting to TH‐positive LC neurons in DBH‐Cre mice. Cre‐dependent AAV helper virus expressing TVA‐mCherry and G protein was injected into the LC nucleus in DBH‐Cre mice, and 3 weeks later, RV‐ΔG‐EGFP virus was injected to infect noradrenergic LC neurons and label their upstream LC‐projecting neurons. Immunofluorescence was performed at 1 week after RV‐ΔG‐EGFP virus injection. MeV was retrogradely labeled by FG after injection into the masseter. (B) Immunofluorescence of GABAergic input neurons of LC neurons. LC neurons were expressing mCherry driven by Cre‐dependent helper virus (DIO‐TVA‐mCherry). RV‐ΔG‐EGFP labeled the starter cells of LC neurons (asterisk indicated) and their input neurons. FG was injected into the masseter to retrogradely label MeV neurons. GAD67 antibody was used to label GABAergic neurons (arrow indicated). Scale bars, 100 µm, 20 µm for enlarged inset. (C) Schematic illustration of Ca^2+^ imaging in GABAergic input neurons to LC neurons in DBH‐Cre mice. Cre‐dependent AAV helper virus, including TVA‐BFP and G protein, together with AAV2/9‐GAD1‐GCaMP6s for Ca^2+^ imaging in GABAergic interneurons, was injected into the LC nucleus in DBH‐Cre mice. Three weeks later, the RV‐ΔG‐dsRed virus was injected to infect noradrenergic LC neurons and label their upstream LC‐projecting neurons. Ca^2+^ imaging was performed at 1 week after RV‐ΔG‐dsRed virus injection. (D) Retrograde labeling of GABAergic input neurons of LC neurons. LC neurons expressed BFP driven by Cre‐dependent helper virus (DIO‐TVA‐BFP). RV‐ΔG‐dsRed labeled the starter cells of LC neurons and their input neurons. GAD1‐GCaMP6s was used to label GABAergic neurons for Ca^2+^ imaging, and those GABAergic input neurons were co‐expressing GCaMP6s and dsRed (circle indicated). Scale bars, 50 µm. (E) Ca^2+^ imaging of GABAergic input neurons of LC neurons. LC neurons were expressed BFP driven by Cre‐dependent helper virus (DIO‐TVA‐BFP), and RV‐ΔG‐dsRed labeled the starter cells of LC neurons and their input neurons. GAD1‐GCaMP6s labeled GABAergic interneurons and those projecting to LC neurons were co‐expressed with dsRed (circle indicated). Scale bar, 50 µm. (F) Statistics of TRPA1 dependence of AITC‐induced Ca^2+^ rise in RV‐dsRed‐positive GABAergic input neurons as in (E) (*n* = 8 neurons). Error bars indicate SEM. ^*^ *p* < 0.05, one‐way ANOVA for (F).

Given that the proprioceptor marker PV has been reported to be expressed in inhibitory neurons in the CNS, we first determined whether any MeV neurons themselves are GABAergic and might directly mediate MeV effects on LC functions. Our single‐cell PCR results demonstrated that DiI‐labeled proprioceptive neurons did not express GAD67 (Figure S15), a marker for GABAergic neurons, suggesting that PV‐expressing proprioceptive neurons in the MeV are not GABAergic. Therefore, we next asked whether local GABA tone was involved. To test this possibility, we injected a mixture of Cre‐dependent helper virus, including AAV2/9‐DIO‐TVA‐mCherry and AAV2/9‐DIO‐RG, into the LC in adult male DBH‐Cre transgenic mice, followed by a second injection of pseudorabies virus RV‐ENVA‐ΔG‐EGFP at the same site of the LC 3 weeks later to retrogradely label the LC^NE^‐projecting neurons (Figure 7A). The proprioceptive MeV neurons were retrogradely labeled by masseter FG injection 7 days prior to dissection (Figure 7B). We found that LC^NE^ neurons were positively labeled by helper virus expressing mCherry, and RV‐EGFP labeled both the LC starter neurons and their upstream projecting neurons, which were distinct from the FG‐labeled proprioceptive MeV neurons but positive for GAD67 immunostaining (Figure 7B). In contrast, RV‐EGFP did not label any presynaptic neurons in control wild‐type (with no Cre expression in LC^NE^ neurons) mice (Figure S16). These findings exclude the direct synaptic connection in MeV‐LC^NE^ transmission and define the monosynaptic transmission from local GABAergic neurons to LC^NE^ neurons.

To determine whether the local GABAergic tone was functionally involved in the MeV‐LC^NE^ transmission, we performed Ca^2+^ imaging in the local GABAergic neurons after the RV‐ENVA‐ΔG retrograde‐labeling. A mixture of Cre‐dependent helper virus, including DIO‐TVA‐BFP and DIO‐RG, together with GAD1‐driven GCaMP6s‐expressing AAV2/9 virus [44], was injected into the LC nucleus in adult DBH‐Cre mice of either sex. Three weeks later, RV‐ENVA‐ΔG‐DsRed was injected in the same site to retrogradely label LC^NE^‐projecting neurons (Figure 7C). Local GABAergic input interneurons were labeled by both DsRed and GCaMP6s (Figure 7D). Interestingly, AITC application (100 µm) induced a gradual increase of intracellular Ca^2+^ signals in DsRed‐positive GABAergic neurons (*n* = 14 neurons from 4 mice), which was attenuated by TRPA1‐antagonist HC‐030031 (50 µm, *n* = 8 neurons, Figure 7E,F). AITC also induced Ca^2+^ rise in DsRed‐negative GABAergic neurons (*n* = 64 neurons in 5 mice).

In parallel, we further verified the firing properties of GABAergic neurons projecting to LC^NE^ neurons. Adult male DBH‐Cre mice received injection of a mixture of Cre‐dependent helper virus including DIO‐TVA‐BFP and DIO‐RG in adult male DBH‐Cre mice, followed three weeks later by the injection of RV‐ENVA‐ΔG‐tdTomato in the same site, while GABAergic neurons were labeled via GAD1‐EGFP AAV. Proprioceptive MeV neurons were retrogradely labeled by masseter 1,1'‐dioctadecyl‐3,3,3',3'‐tetramethylindodicarbocyanine, 4‐chlorobenzenesulfonate salt (DiD) injection 7 days prior to dissection. Immunofluorescence confirmed the presence of GABAergic neurons situated between MeV and the LC nucleus (Figures S17 and S18D). Consistent with the Ca^2+^ imaging data, AITC increased the firing frequency of these GABAergic neurons (Figure S18B,C).

To determine the transmitter released from MeV neurons to engage GABAergic neurons, we micro‐injected a Cre‐dependent EYFP AAV into MeV of adult male PV‐Cre mice to label proprioceptive neurons. Immunostaining revealed that most EYFP‐positive MeV neurons are vesicular glutamate transporter 1 (VGLUT1)‐positive (Figure S19), consistent with previous reports that MeV proprioceptive neurons are glutamatergic [11, 46]. This supports glutamate as a principal excitatory transmitter released from MeV somata. Moreover, pharmacological blockade of AMPA and NMDA receptors significantly attenuated AITC‐induced inhibition in LC neuronal AP frequency (Figure S20). In contrast, application of AITC failed to increase the amplitude or frequency of spontaneous excitatory post‐synaptic currents (EPSCs) of GABAergic input neurons (Figure S21), suggesting a key role for non‐synaptic but somatic secretion of glutamate transmission in MeV‐GABA‐LC signaling.

Because proprioceptive MeV neurons are pseudounipolar neurons that show no local synaptic projection, we further verified whether there is no direct synaptic connection from MeV neurons to nearby GABAergic neurons. We retrogradely labeled the input GABAergic neurons around the LC nucleus by injecting a mixture of Cre‐dependent helper virus, including DIO‐TVA‐BFP and DIO‐RG, in the LC nucleus in adult male GAD‐Cre mice [44], which was followed by the injection of RV‐ENVA‐ΔG‐DsRed in the same site three weeks later (Figure S22A). As expected, local GABAergic starter interneurons were labeled by both BFP and DsRed, and their input neurons were labeled with DsRed, and showed no overlap with MeV neurons (Figure S22B). These findings suggest that there is no direct projection from MeV proprioceptive neurons to local GABAergic neurons. Thus, TRPA1‐mediated somatic secretion of MeV neurons will be essential for the activation of local GABAergic interneurons.

**FIGURE 8:**
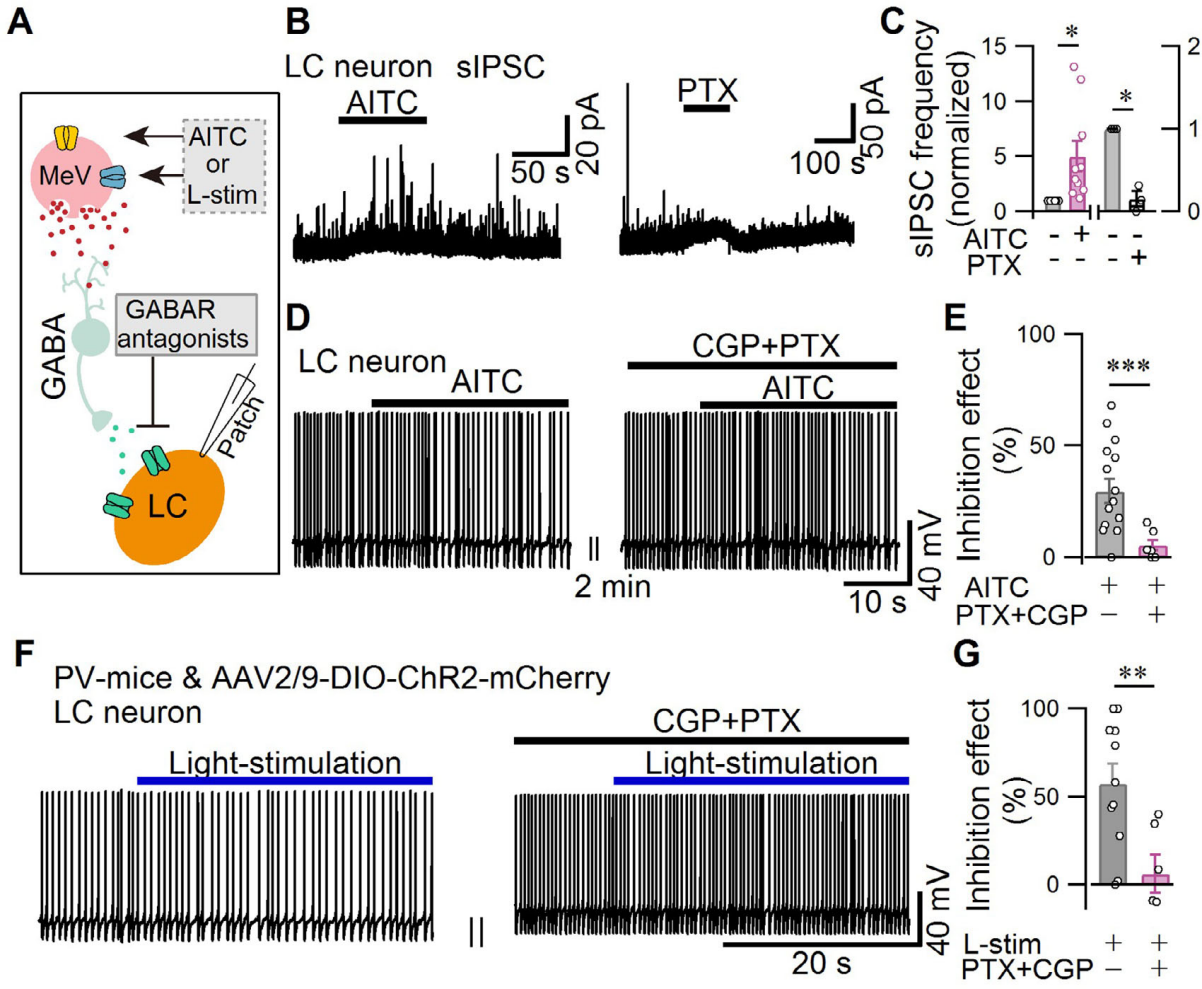
Local GABA neurons mediate the cross‐talk between proprioceptive MeV and LC^NE^ neurons. (A) Schematic illustration of GABAR dependence of inhibition of LC neuronal activity upon pharmacological or light stimulation (L‐stim) of MeV neurons. (B) Representative traces showing the effect of AITC (100 µm) on the frequency of IPSCs in LC neurons, and GABA receptor dependence of IPSCs with GABA_A_ antagonist picrotoxin (PTX, 100 µm). (C) Statistics of (B) (*n* = 10 neurons/group for AITC experiment, and *n* = 3 neurons/group for PTX experiment). (D–G) Representative traces and statistics showing GABA receptor dependence of inhibition of LC firing in brain slices upon AITC activation in wild‐type mice (D, E) or light stimulation with AAV2/9‐DIO‐ChR2‐mCherry injection in the MeV nucleus in PV‐Cre mice (F, G). GABA_A_ receptor antagonist PTX (100 µm) and the GABA_B_ antagonist CGP55845 (CGP, 10 µm) were co‐applied to block GABA transmission (*n* = 6–14 neurons/group for AITC stimulation and *n* = 5–11 neurons/group for light stimulation). Blue light pulses of 20 Hz (5 ms duration for each pulse) were applied for 30 s to stimulate ChR2‐expressing MeV neurons in PV‐Cre mice. Error bars indicate SEM. ^*^ *p* < 0.05, ^**^ *p* < 0.01, ^***^ *p* < 0.001, paired Student's *t*‐test for (C), unpaired Student's *t*‐test for (E, G).

To confirm the critical roles of local GABA neurons in MeV‐LC^NE^ transmission, we recorded the spontaneous inhibitory post‐synaptic currents (IPSCs) in LC^NE^ neurons in response to the excitation of MeV neurons in adult male mice (Figure 8A). We found that local application of AITC (100 µm) increased the frequency of sIPSC in LC^NE^ neurons, which was blocked by GABA_A_ receptor antagonist picrotoxin (PTX, 100 µm) (Figure 8B,C). Similarly, AITC‐mediated reduction in the firing rate of LC^NE^ neurons was also mostly abolished by the combined application of PTX and the GABA_B_ receptor antagonist CGP55845 (10 µm) (Figure 8D,E), suggesting the involvement of local GABA tone in MeV‐LC^NE^ transmission. Consistently, local application of GABA (100 µm) indeed blocked the firing of LC^NE^ neurons, which was attenuated by PTX and CGP (Figure S23). Moreover, optogenetic activation of MeV neurons decreased the activity of LC^NE^ neurons, which was also significantly blocked by the cocktail of GABA receptor antagonists (Figure 8F,G; Figure S24). These data further confirm the involvement of local GABA transmission in the crosstalk between the proprioceptive MeV and the pain‐modulating LC^NE^ neurons. Finally, local GABA tone‐dependent reduction of descending pain inhibition from the LC after inflammation facilitates the development of muscle pain.

Collectively, these findings identified TRPA1‐mediated proprioceptive neuronal secretion in the MeV and delineate a central mechanism underlying the development of inflammatory masseter muscle pain through somatic transmission from the proprioceptive MeV neurons to the descending pain‐modulatory noradrenergic system via local GABAergic signaling. This work establishes an elegant model in which inflammation‐induced sensitization of proprioceptive neurons weakens LC‐mediated endogenous pain inhibition and thereby facilitates persistent muscle pain.

## 3 Discussion

We have identified a novel form of neural communication via somatic but not synaptic transmission from the proprioceptive MeV neurons to local GABA neurons, which then modulates LC^NE^ neuronal activity via synaptic GABAergic transmission. This local dual‐mode MeV‐GABA‐LC^NE^ transmission conveys the proprioceptive information to the endogenous noradrenergic descending pain pathway and promotes inflammatory muscle pain (Figure S25). Specifically, we demonstrated that (1) TRPA1 is functionally expressed in proprioceptive MeV neurons and upregulated upon masseter inflammation; (2) TRPA1 KD or TeNT‐mediated clearance of SNARE‐dependent somatic exocytosis of MeV neurons attenuates orofacial pain responses; (3) TRPA1 activation induces mechanical hyperalgesia which is SNARE‐dependent; (4) In the absence of direct synaptic connections, MeV neuronal excitation reduces noradrenergic neuronal activity in the LC through local GABA tone. Together, these findings support a model in which chemical cross‐talk between proprioceptive MeV neurons and LC^NE^ neurons provides a new central mechanism for proprioceptive modulation of masseter muscle pain.

TRPA1 serves as an irritant sensor in primary sensory DRG/TG neurons and contributes to injury‐induced hyperalgesia via nociceptive transduction [22, 32, 33]. Although evidence shows that neuronal TRPA1 is expressed in some brain regions, including the somatosensory cortex and cerebellum [47, 48], its role in the central nervous system remains largely unknown. Here, we validated the functional expression of TRPA1 in the proprioceptive MeV neurons located in the brain, in both neonatal rats, neonatal mice, and adult mice. This expression showed a consistent expression pattern across species and ages (Figures 1 and 3; Figures S13 and S15). TRPA1 was elevated following masseter inflammation and peaked at day 3 after inflammation in expression of its mRNA and protein as well as functional changes (Figure 2). This is similar to the time‐course of TRPA1 expression in TG neurons in response to inflammatory stimulation [33, 49], suggesting that MeV and TG neurons are responsive to the irritant stimulation from the masseter muscle simultaneously.

Our previous work identified Ca^2^^+^‐dependent somatic exocytosis in MeV neurons [50] and TRPA1‐Ca^2^^+^‐mediated exocytosis in DRG neurons [30]. In the present study, we extend these findings by confirming TRPA1 expression in MeV neurons and demonstrating that TRPA1‐driven Ca^2^^+^ elevation triggers somatic exocytosis in these cells (Figures 1 and 3; Figure S9). We also demonstrated that MeV somatic secretion is SNARE‐dependent, as shown by cleavage of VAMP2 (Figure 3). TRPA1‐mediated Ca^2^^+^ influx likely plays a key role in exocytosis by regulating both vesicle priming and fusion processes. Following their formation in the trans‐Golgi network, secretory vesicles are translocated to the cytosol and docked at the plasma membrane, where they join the releasable pool. Vesicle “priming” then occurs, during which SNARE complexes—comprising v‐SNARE (VAMP proteins on vesicles) and t‐SNARE (syntaxin and SNAP25 on the plasma membrane)—assemble to render vesicles release‐ready [51, 52]. Moderate elevations in intracellular Ca^2^^+^ are crucial for this priming step, sensed by proteins such as mammalian uncoordinated‐13 (Munc13) and calcium‐dependent activator protein for secretion (CAPS) [52, 53]. Subsequent substantial Ca^2^^+^ rises trigger conformational changes in SNARE complexes and lead to vesicle fusion. This fusion step is likely mediated by synaptotagmin proteins—including synaptotagmin‐1, ‐7, and ‐11—which bind Ca^2^^+^ via their C2 domains and facilitate SNARE‐mediated membrane fusion [53, 54, 55, 56].

Masseteric inflammation upregulates TRPA1 expression in MeV neurons (Figure 2). Given that Ca^2^^+^ sensors such as Munc13, CAPS, and synaptotagmins facilitate Ca^2^^+^‐dependent vesicle priming and fusion [51, 52, 53, 54, 55, 56]. Our results suggest that TRPA1‐mediated exocytosis in MeV neurons may be potentiated during the development of orofacial muscle pain. Consistent with this idea, genetic blockade of the Ca^2+^ rise and SNARE‐dependent vesicular exocytosis of MeV neurons by TRPA1‐KD or TeNT‐overexpression significantly attenuated the inflammatory mechanical hyperalgesia (Figures 2M and 3), while pharmacological activation of TRPA1 in MeV induced mechanical hyperalgesia in naïve mice (Figure S11), but failed to do so in TeNT‐treated inflamed mice (Figure S12). Together, these findings support a pivotal role for the TRPA1‐mediated elevation of MeV neuronal secretion during the development of inflammatory muscle pain.

We also found a transient reduction in the bite force after masseter inflammation (Figure 2L), which is consistent with previous reports in rodents [35, 37, 57] and patients with orofacial pain [58]. Proprioceptive MeV neurons innervate the periodontal ligaments and muscle spindles to modulate bite force and jaw stretch reflex, respectively [3, 59]. In the present work, we found that TRPA1‐KD in MeV neurons significantly restored the bite‐force reduction during masseter inflammation (Figure 2L). Proprioceptive behaviors in the body are usually examined using indices such as balance, body posture, limb position, and body movement with the assistance of the movement extent discrimination apparatus [60, 61, 62]. Bite force measurement includes the proprioceptive component and can be used to discriminate the proprioceptive changes in the orofacial region. Thus, the restored bite force in TRPA1‐KD mice suggests a critical role of MeV TRPA1 in the inflammation‐induced proprioceptive changes and provides a novel scenario that TRPA1‐dependent proprioceptive secretion underlies the development of muscle pain.

The LC nucleus, which lies in close proximity to the MeV nucleus [63, 64], is the principal source of norepinephrine in the brain and a well‐established hub for descending pain inhibition and anti‐hyperalgesia [20, 42, 43, 65]. Strikingly, our findings identify a unique communication between proprioceptive MeV neurons and nociceptive LC^NE^ neurons despite no direct synaptic connections between them, which serves as a central mechanism underlying the endogenous facilitation of inflammatory pain. This is strongly supported by the following evidence: (1) TRPA1 activation evokes SNARE‐dependent somatic exocytosis of MeV neurons (Figure 3C,D); (2) local application of TRPA1 agonist reduces the spontaneous firing of nearby LC^NE^ neurons in brainstem slices in situ, which was further enhanced in animals with masseter inflammation (Figure 4B,C); (3) VGLUT‐1 immunostaining in proprioceptive MeV neurons identifies glutamate as an excitatory transmitter (Figure S19), in line with previous studies [11, 46]; accordingly, a cocktail of NMDA and AMPA receptor antagonists attenuates the TRPA1‐mediated inhibition of LC^NE^ neuronal activity (Figure S20); (4) TRPA1‐KD in the MeV enhances inflammation‐induced c‐Fos expression in the LC in CFA‐inflamed mice (Figure 4D,E); (5) genetic ablation of MeV exocytosis prevents TRPA1 activation‐induced inhibitory effect on LC^NE^ neuronal firing (Figure 4F,G); (6) optogenetic activation of proprioceptive MeV neurons inhibits LC^NE^ neuronal firing in situ (Figure 5); and (7) Chemogenetic activation of LC^NE^ neurons attenuates inflammatory mechanical hyperalgesia (Figure 6). These findings together strongly suggest a TRPA1‐dependent inhibitory effect from the MeV to LC^NE^ neurons.

LC^NE^ neurons are a well‐established central hub for inhibiting inflammatory hyperalgesia both in body and orofacial areas through the descending pathway [66, 67, 68, 69]. Although multiple reports demonstrate that activation of LC^NE^ neurons inhibits the CFA‐induced persistent hyperalgesia in both hindpaw [66, 70, 71] and orofacial area [72, 73, 74], whether and how proprioceptive inputs are functionally involved in descending pain modulation remains virtually unknown. Our findings suggest that the MeV‐GABA‐LC^NE^ dual‐mode communication (somatic‐synaptic transmission) leads to the disinhibition of the descending pain and thus contributes to proprioceptive facilitation of the development of orofacial muscle pain. Both immunostaining and single‐cell RT‐PCR excluded the possible secretion of GABA from proprioceptive MeV neurons (Figure 7B; Figure S15). Importantly, RV‐EGFP retrograde labeling showed that MeV neurons do not directly project to LC^NE^ neurons; instead, there is a direct projection from the neighboring local GABAergic neurons to LC^NE^ neurons (Figure 7B: Figures S17 and S18). Interestingly, TRPA1 activation of MeV neurons elicited the intracellular Ca^2+^ rise and firing frequency of GABAergic input neurons (Figure 7; Figure S18), and increased the GABAergic IPSCs in LC^NE^ neurons in brainstem slices (Figure 8B,C). Consistently, GABA receptor antagonists fully blocked the inhibitory effect on LC^NE^ neurons mediated by either TRPA1 activation or optogenetic excitation of MeV neurons (Figure 8). These data suggest that local GABA tone functionally mediates proprioceptive involvement in endogenous descending pain modulation through MeV‐LC communication.

Importantly, we found that MeV neurons modulate local GABAergic neurons via somatic volume transmission but not the classic synaptic transmission. Although extrasynaptic or volume transmission is proposed to be an alternative form of neural communication, somatic transmission has long been ignored due to the lack of functional evidence and physiological relevance. Instead, the MeV resides in the brain. It is an evolutionarily conserved primary sensory neuron with classic pseudounipolar morphology, which mainly innervates the trigeminal motor, the main sensory nucleus, the brainstem reticular formation, and the cerebellum [11], but not the neighboring area around the LC and MeV where GABA neurons localized (Figure S22). Furthermore, TRPA1 activation of MeV neurons failed to increase the spontaneous IPSCs in GABAergic neurons (Figure S21), suggesting that traditional synaptic transmission is unlikely to participate in the cross‐talk between MeV and local GABAergic neurons. Our previous findings have shown the existence of somatic exocytosis [15], which is able to affect adjacent cells in the CNS and the peripheral nervous system [20, 75, 76]. TRPA1 activation induces the somatic exocytosis of MeV neurons (Figure 3A–D), and genetic ablation of this exocytosis with TeNT blocked the inhibitory effect of TRPA1 activation on LC^NE^ neuronal excitability in brainstem slices in situ (Figure 4F,G), suggesting that MeV somatic exocytosis is essential for the signaling transduction from the proprioceptive MeV neurons to the nociceptive LC^NE^ neurons through the local GABA tone (Figures 3, 4, 5, 6). Collectively, these findings for the first time defined a new functional communication involving non‐synaptic transmission from proprioceptive MeV neurons to the pain‐related LC^NE^ neurons in the CNS.

Given the close anatomical apposition between MeV and LC nuclei and the possibility of broad somatic transmission [20, 75, 76], a potential direct modulatory effect of MeV neurons on LC neurons warrants attention. Prior reports have shown that glutamate application increases LC neuronal firing and somatic norepinephrine release [77, 78, 79]. In our experiments, following GABAR blockade to eliminate GABAergic contributions, activation of MeV neurons did not significantly alter LC^NE^ firing frequency (Figure 8D–G). These results suggest that any direct effect of MeV neuron input onto LC neurons is likely minimal. The differential effect of MeV somatic secretion on LC^NE^ and GABAergic neurons might be attributed to distinct glutamate receptor expression patterns or heterogeneous neuronal input integration strategies [44, 80].

Altogether, this work delineates a new mode of neural communication between the proprioceptive MeV neurons and the pain‐modulating LC^NE^ neurons via combined somatic volume transmission with classical synaptic signaling. This circuit reveals a crucial role for TRPA1‐mediated sensitization of proprioceptive MeV neurons in weakening descending noradrenergic pain inhibition and thereby driving the development of orofacial muscle pain. It also provides a compelling example of central somatic secretion contributing to brain functions and potentially to CNS disorders. The principle of multisensory neuronal cross‐talk described here may extend to functional pain syndromes, such as idiopathic low back pain and fibromyalgia. These findings further suggest new avenues for targeting proprioceptive rehabilitation in the treatment of chronic muscle pain and related pain syndromes. In addition, given the unique chemical crosstalk between the MeV and the LC, and the well‐established roles of LC noradrenergic signaling in attention, emotion, arousal, and vigilance, this study also sheds new light on the functional link between proprioception and the related neurological disorders (e.g., anxiety, depression, sleep disorders, and attention deficits).

## 4 Materials and Methods

### 4.1 Animals

The protocols were approved by the Animal Care and Use Committees of Peking University and the University of Maryland, Baltimore, as well as by the Association for Assessment and Accreditation of Laboratory Animal Care (IACUC# IMM‐Zhouz‐11). Experiments were performed on brainstem slices from Sprague‐Dawley rats and mice (C57BL/6) of either sex unless otherwise specified. Rats and TRPA1‐KO mice (C57BL/6, a gift from Dr. David Julius of the University of California, San Francisco) at P7‐8 were decapitated, and their brain were sliced for patch‐clamp recording and Ca^2+^ imaging as described previously [15, 20]. Adult C57BL/6 mice and the transgenic mouse lines (DBH‐Cre mice, GCAMP 5 transgenic mice from Dr. Heping Cheng in Peking University, and PV‐Cre mice from Dr. Zhili Huang and Dr. Miao He in Fudan University) [45, 81] were anesthetized with isoflurane as described [49] for ipsilateral intra‐masseter muscle injection of CFA (0.5 mg/mL, 20 µL, Sigma).

For upstream labeling of neurons projecting to LC^NE^ neurons, adult male DBH‐Cre mice or control wild‐type C57 mice were anesthetized with 1.25% avertin (250 mg/kg, i.p.), and received a unilateral injection of a mixture of AAV2/9‐EF1α‐DIO‐TVA‐mCherry and AAV2/9‐EF1α‐DIO‐oRG (1:1, 500 nL) (BrainVTA company, Wuhan, China) into the left local LC nucleus: coordinates 5.5 mm caudal to bregma, −0.95 mm lateral, and 3.75 mm deep, and a second injection of 500 nL RV‐ENVA‐ΔG‐EGFP (BrainVTA company, Wuhan, China) was injected in the same site 3 weeks later. Immunostaining was done at day 7–10 after RV virus injection.

For upstream labeling of GABAergic projecting neurons of LC^NE^ neurons, adult male DBH‐Cre mice or control wild‐type C57 mice were anesthetized with 1.25% avertin (250 mg/kg, i.p.), and were injected with a mixture of AAV2/9‐EF1α‐DIO‐TVA‐BFP, AAV2/9‐EF1α‐DIO‐oRG and AAV2/9‐GAD1‐EGFP (1:1, 500 nL) (BrainVTA company, Wuhan, China) in left local LC nucleus: coordinates 5.5 mm caudal to bregma, −0.95 mm lateral, and 3.75 mm deep. Two to three weeks later, a second injection of 500 nL RV‐ENVA‐ΔG‐tdTomato (BrainVTA company, Wuhan, China) was made into the same coordinates. Patch clamp and immunostaining were done at day 7–10 after RV virus injection

For upstream labeling of GABAergic neurons projecting to LC^NE^ neurons, adult male GAD‐Cre mice (a generous gift from Dr. Bo Zhang of Peking University Shenzhen Graduate School) were anesthetized with 1.25% avertin (250 mg/kg, i.p.), and received an injection of a mixture of AAV2/8‐EF1α‐DIO‐TVA‐BFP, AAV2/9‐EF1α‐DIO‐oRG (1:1, 500 nL) (BrainVTA company, Wuhan, China) into the left local LC nucleus: coordinates 5.5 mm caudal to bregma, −0.95 mm lateral, and 3.75 mm deep. Three weeks later, a second injection of 500 nL RV‐ENVA‐ΔG‐DsRed (BrainVTA company, Wuhan, China) was made into the same coordinates. Immunostaining was done at day 7–10 after RV virus injection.

For Ca^2+^ imaging of GABAergic neurons projecting to LC^NE^ neurons, adult male DBH‐Cre mice were anesthetized with 1.25% avertin (250 mg/kg, i.p.), and received an injection of a mixture of AAV2/8‐EF1α‐DIO‐TVA‐BFP, AAV2/9‐EF1α‐DIO‐oRG and AAV2/9‐GAD1(m)‐GCaMP6s (1:1:1, 500 nL, BrainVTA company, Wuhan, China) into the left local LC nucleus: coordinates 5.5 mm caudal to bregma, −0.95 mm lateral, and 3.75 mm deep. Three weeks later, a second injection of 500 nL RV‐ENVA‐ΔG‐DsRed (BrainVTA company, Wuhan, China) was made into the same coordinates. Ca^2+^ imaging was performed 7–10 days after RV virus injection.

For genetic knockdown of SNARE protein, adult male PV‐Cre mice were anesthetized with 1.25% avertin (250 mg/kg, i.p.), and received an injection of 300 nL control AAV2/9‐hysn‐DIO‐mCherry or AAV2/9‐hysn‐DIO‐TeNT‐mCherry (OBiO Technology company, Shanghai, China) into the left MeV nucleus: coordinates 5.4 mm caudal to bregma, −1.0 mm lateral, and 3.9 mm deep. The virus was injected using a micropipette into the target site at a rate of approximately 1.6 nL/s. Mice were allowed to recover for about 4 weeks before subsequent experiments.

For optogenetic stimulation, 500 nL Cre‐dependent AAV2/9‐EF1α‐ChR2 (AV‐9‐20298P, Penn Vector Core, Philadelphia, PA) was injected into the MeV nucleus in PV‐Cre mice after anesthetization with 1.25% avertin (250 mg/kg, i.p.) [45]: bilateral at the coordinates 5.4 mm caudal to bregma, ± 1.1 mm lateral, and 3.9 mm deep. After recovery for ∼1 month, optogenetic stimulation was performed in ChR2‐expressing brain slices as previously described [81] with minor modifications. Briefly, 470‐nm light from a light‐emitting diode (Yibo Instruments, Wuhan, China) for optogenetic stimulation was focused onto the tissue (illuminating a field of approximately 440 µm in diameter) through a microscope under a 60× water‐immersion lens. The 510‐nm light from a monochromator (T.I.L.L. Photonics, Martinsried, Germany) was used to visualize EYFP expression.

### 4.2 For Cannula Implantation

Cannula‐guided microinjection of pharmacological agents was combined with von Frey testing in awake mice, both with and without prior virus injection. A single guide cannula (O.D. 0.41 mm) was implanted into the left MeV nucleus at the following coordinates: 5.4 mm caudal to bregma, 1.0 mm lateral, and 3.5 mm deep. The guide cannula was secured using light‐cured gingival barrier resin, and a dummy cannula was inserted to prevent clogging. After a minimum recovery period of 7 days, an injector cannula (O.D. 0.41 mm) was inserted into the guide cannula for microinjection at a rate of 0.15 µL/min for 1 min, controlled by a microinfusion pump (KD Scientific, Holliston, USA) connected to a 10 µL Hamilton syringe. AITC (0.1 mg/mL in saline) or saline alone was infused 5 min prior to the von Frey test. Microinjection sites were verified by injecting Chicago Sky Blue and performing histological sectioning after behavioral testing; mice with incorrect microinjection placements were excluded from analysis.

### 4.3 Human Tissue

The use of paraffin‐embedded sections of human tissue was approved by the Neuroscience Center, Chinese Academy of Medical Sciences, and the Chinese Human Brain Banking Consortium (Project Number: 009–2014).

### 4.4 Slice Preparation

Brain slices from neonatal rats/mice containing the MeV and LC were prepared as described previously [15, 20]. In brief, horizontal MeV and LC slices were cut at 300 µm on a vibratome (Leica VT 1000s, IL, USA) in ice‐cold cutting solution (in mm): 252 sucrose, 5 KCl, 2 CaCl_2_, 2 MgCl_2_, 26 NaHCO_3_, 2 HEPES, 10 D‐glucose (saturated with 95% O_2_/5% CO_2_), and then were incubated in recording artificial cerebrospinal fluid (“neonatal aCSF”) (in mm): 126 NaCl, 5 KCl, 2 CaCl_2_, 2 MgCl_2_, 26 NaHCO_3_, 2 HEPES, 10 D‐glucose at 37°C for 30 min (saturated with 95% O_2_/5% CO_2_), after which the slices were moved to aCSF at room temperature. The 50 mm KCl solution contained (in mm): 81 NaCl, 50 KCl, 2 CaCl_2_, 2 MgCl_2_, 26 NaHCO_3_, 2 HEPES, 10 D‐glucose. Slices from 25‐g mice were prepared as described [81] with minor modifications. Briefly, mice were anesthetized, and the brain was removed after transcardial perfusion with ice‐cold cutting solution (in mm): 110 C_5_H_14_NClO, 2.5 KCl, 7 MgCl_2_, 0.5 CaCl_2_, 25 NaHCO_3_, 1.3 NaH_2_PO_4_, 25 glucose, 5 L‐ascorbic acid, 3 Na‐pyruvate (saturated with 95% O_2_/5% CO_2_) and cut at 250 µm in aCSF on the vibratome. Then slices containing the MeV and LC were incubated in recording “adult aCSF” containing (in mm): 125 NaCl, 2.5 KCl, 1.3 MgCl_2_, 2 CaCl_2_, 25 NaHCO_3_, 10 glucose (saturated with 95% O_2_/5% CO_2_) at 37°C for 30 min before being moved to room temperature.

### 4.5 Generation of Cyto‐GCaMP5A Transgenic Mice

To measure the cytosolic Ca^2+^ dynamics in acute MeV slices from adult mice, we used a cyto‐GCaMP5A transgenic mouse line, a generous gift provided and created by Dr. Heping Cheng's lab using a protocol described previously [82]. GCaMP5A DNA was driven by the chicken β‐actin promoter and integrated into the pUC‐CAGGS expression vector downstream of the promoter at the XhoI site with an In‐Fusion cloning kit (Clontech, CA, USA). The targeting vector with the promoter and β‐globin polyadenylation signal was linearized with SpeI and HindIII, and then transfected into mouse embryonic stem cells to generate transgenic founders. Primers for genotyping were synthesized as follows: 5'‐ATGGGGATGGTCAGGTAAACTA‐3' / 5'‐GAATAAGGAATGGACAGCAGGG‐3'. PCR was conducted with 2× EasyTaq PCR SuperMix (AS111‐11, Transgen, Beijing, China) in 20 µL under a PCR program of 95°C for 1 min, followed by 34 cycles of 95°C for 30 s, 62°C for 30 s, and 72°C for 30 s, and ended with 72°C for 2 min.

### 4.6 Retrograde Labeling

Rats of either sex at P7 were used for the retrograde labeling of MeV neurons. Briefly, 30 µL of DiI (50 mg/mL, Invitrogen, CA, USA) was injected into the masseter muscle on one side in neonatal rats. After 7–10 days for recovery, the animals were sacrificed by decapitation, and the brains were cut at 100 µm on a vibratome (Leica VT 1000s, IL, USA). Slices containing the MeV were immediately fixed in 4% paraformaldehyde in phosphate‐balanced saline (PBS) for 1 h. After three washes in PBS, the sections were incubated with 1 µg/mL DAPI in PBS for 5 min and then washed with PBS for 10 min. Slices were examined under a Zeiss 710 inverted confocal microscope. Images were processed with ImageJ (National Institutes of Health, MD, USA) and Adobe Photoshop CS5 (Adobe Systems Inc., CA, USA). For retrograde tracer tracing in the adult male mice, animals were deeply anesthetized by intraperitoneal injection of ketamine‐xylazine (100 and 10 mg/kg, respectively) or 1.25% avertin (250 mg/kg, i.p.), then 20 µL DiI (50 mg/mL, Invitrogen, CA, USA), 10 µL FG (4%, Fluorochrome, CO, USA) or 20 µL DiD (4%, Invitrogen, CA, USA) was injected into the masseter muscle or gingival tissue as indicated. After 6–7 days of recovery, the mice were sacrificed and perfused for fixation. Brain sections containing the MeV injection site were cut at 40 µm using a Leica cryostat, and positive signals were subsequently detected using the method described above.

### 4.7 Immunohistochemistry

Immunohistochemistry was performed as described previously [81, 83] with slight modifications. Briefly, mice were anesthetized and perfused with saline and then 4% paraformaldehyde in PBS. The brain was then fixed in 4% paraformaldehyde in PBS for 24 h and dehydrated in 30% sucrose. Coronal sections of the brainstem containing the MeV were cut at 40 µm on a Leica cryostat. The sections were washed three times with PBS for 5 min each. Then, they were permeabilized with 0.3% Triton X‐100 in PBS containing 2% bovine serum albumin (BSA) at room temperature for 15 min. Paraffin‐embedded sections from human samples containing the MeV were deparaffinized in xylene, rehydrated, and then heated in antigen‐retrieval solution (in mm): 10 Tris, 1 EDTA, pH 9.0 for epitope retrieval. After blocking both the human and rodent sections with 2% BSA in PBS for 1 h, they were incubated with primary antibodies for 1 h. Antibodies against TRPA1 (Santa Cruz, SC‐32355, 1:500), tyrosine hydroxylase (Millipore, AB152, 1:1000), glutamate decarboxylase (GAD67; Millipore, MAB5406, 1:500), parvalbumin (SWANT, 235, 1:3000), dopamine‐beta‐hydroxylase (Immunostar, 22806, 1:2000), VGLUT1 (Sigma，MAB5502, 1:500) and c‐Fos (Santa Cruz, SC‐52, 1:10 000) were used as primary antibodies. After washing three times with 2% BSA in PBS, sections were incubated with Alexa Fluor 488, 594, or 633‐labeled secondary antibodies (Invitrogen, A11055, A21206, A21203, A21071, 1:5000) for 1 h at room temperature. After three washes with 2% BSA in PBS, sections were incubated in 1 µg/mL DAPI in PBS for 5 min and then washed with PBS for 10 min. The specificity of the above antibodies was verified by antibody absorption tests or in a KO mouse line. Samples were mounted and imaged on a Zeiss LSM 700 microscope (Carl Zeiss, Oberkochen, Germany) or an EVOS FL Auto microscope (Thermo Fisher Scientific, OH, USA). Images were analyzed with ImageJ (NIH, MD, USA) and Adobe Photoshop CS5 (Adobe Systems, CA, USA).

### 4.8 Electrophysiology

We used an EPC/10 amplifier (HEKA Electronik, Lambrecht/Pfalz, Germany) and Pulse software (HEKA Electronik) to record somatic patch‐clamp signals (whole‐cell membrane currents, potentials, and capacitance) as described previously [15, 20]. The pipette resistance was 3–4 MΩ, and the average access series resistance of the neurons was <10 MΩ. Off‐line analysis was performed using Igor software (Wavemetrics, OR, USA). For TRPA1 current recording and Cm recordings in neonatal rats, the standard intracellular solution was (in mm): 153 CsCl, 1 MgCl_2_, 10 HEPES, 4 Mg‐ATP (pH 7.2–7.4). Allyl isothiocyanate (AITC; 100 µm) was included in the extracellular solution to activate TRPA1 channels. For action potential recordings in adult mice, the standard intracellular solution was (in mm) 130 K‐gluconate, 10 KCl, 2 MgCl_2_, 10 HEPES, 2.5 Mg‐ATP, 0.25 Na‐GTP (pH 7.2‐7.4). For Cm recording, EPSCs and IPSCs recording in adult mice, the standard intracellular solution was (in mm): 135 CsMeSO_3_, 1 EGTA, 10 HEPES, 8 Na_2_‐phosphocreatine, 4 Mg‐ATP, 3.3 QX‐314, 0.3 Na‐GTP (pH 7.2–7.4). Minianalysis (Synaptosoft, NJ, USA) was used to analyze IPSCs. All drugs were from Sigma (MO, USA) unless otherwise indicated. Data were digitized at 10 kHz and filtered at 2.9 kHz.

### 4.9 [Ca^2+^]_i_ Imaging

Ca^2+^ images of MeV neurons in P7‐8 rats/mice were collected with TILLvision software (TILL Photonics, Planegg, Germany) as described previously [15]. MeV slices were loaded with 15 µm Fura‐2 AM (Invitrogen, CA, USA) at 37°C for 30 min and washed twice with aCSF. Then the slices were imaged on an Olympus BX51 WI microscope (Olympus Optical, Tokyo, Japan) equipped with a 60× water‐immersion lens. Ca^2+^ signals were calculated from the ratio of the fluorescent signals excited at 340 and 380 nm. Ca^2+^ signals from the GCaMP‐expressing mice were recorded on a Zeiss 700 upright confocal microscope when excited at 488 nm and calculated with the equation ΔF/F_0_ (ΔF = F−F_0_)_._

### 4.10 Western Blot

As described previously [83], samples from CFA‐treated and control mice were homogenized in RIPA lysis buffer with a protease inhibitor cocktail (Millipore, 539134), then centrifuged at 13 000 rpm at 4°C for 15 min. Then, 20 µL boiled samples with loading buffer (40 mm Tris‐HCl, pH 8.0, 200 mm DTT, 4% SDS, 40% glycerol, 0.032% bromophenol blue) were loaded onto a 10% Tris‐HCl SDS‐PAGE gel. Then the proteins were transferred to a nitrocellulose blotting membrane (Life Science, T605311). The membrane was blocked with 5% non‐fat milk in PBST (PBS with 0.5% Tween 20) for 1 h at room temperature, and then incubated with primary antibodies in PBST with 2% BSA overnight at 4°C. The primary antibodies were TRPA1 (Millipore, ABN1009, 1:1000) and β‐actin (Sigma, A5316, 1:5000). After that, the membranes were washed three times with PBST for 10 min each, and incubated with the secondary antibodies goat anti‐rabbit IgG (H+L) (Li‐Cor, 926–32211; 1:5000) and donkey anti‐goat IgG (H+L) (Li‐Cor, 926–68074; 1:5000) for 1 h at room temperature. Then the membranes were washed three times with PBST. The proteins were visualized on an Odyssey dual color infrared fluorescence imaging system, and the images were analyzed and calibrated with ImageJ.

### 4.11 Quantitative Real‐Time PCR

Samples were ground under liquid nitrogen, and their RNA was extracted with RNAiso Plus (Takara, D9108A) following the manual; quality was evaluated by electrophoresis on 2% agarose gel. The mRNA concentration was quantified using a spectrophotometer (NanoDrop, ND‐2000). First‐strand cDNA was synthesized with TransScript‐Uni One‐Step gDNA Remover and cDNA Synthesis Supermix (Transgene, AT311‐02) using anchored oligo (dT)_20_ primer and 5 µg total RNA as template.

Real‐time PCR was conducted with PowerUp SYBR Green Master Mix (Applied Biosystems, A25741) using a CFX96 touch real‐time PCR detection system (Bio‐Rad). Reactions were run in triplicate in 10 µL under a program of 50°C for 2 min to activate uracil‐DNA glycosylase and 95°C for 2 min to activate polymerase, followed by 40 cycles of 95°C for 15 s and 64°C for 30 s, then 72°C for 45 s. The relative TRPA1 expression was calculated using the 2^−ΔΔCt^ method. The primer sequence for Trpa1 was 5'‐CAATGCTGACATCCTCCTGAAC‐3' / 5'‐GGACATCGATTGCTTGGA‐GAATTA‐3'; and for mouse Actb, 5'‐GTCCCTCACCCTCCCAAAAG‐3' / 5'‐GCTGCCTCAACACCTCAACCC‐3'

### 4.12 Single‐Cell Amplification and RT‐PCR

Single MeV or LC neurons in slices from 20 to 25 g male mice were picked out and deposited in lysis buffer with a glass micropipette, then PolyA(+) RNA was transcribed and cDNA generated as previously described. The cDNA was amplified in a 20 µL PCR reaction containing 2 µL cDNA, 10 µL 2×Taq PCR starMix with loading dye (GenStar, Shenzhen, China), and 250 nm primer. PCR assays were started with an initial denaturation at 95°C for 2 min followed by 35 cycles of 95°C for 30 s (denaturation), 62°C for 30 s (annealing), and 72°C for 30 s (primer extension). Final extension was carried out at 72°C for 5 min. The primer sequences for Trpa1 and Actb were used as above in quantitative real‐time PCR; the primer sequence for Pvalb was 5'‐TTGCTCTGCCCGCTCAAAC‐3'/5'‐TCAGAATGGACCCCAGCTCATC‐3'; for mouse Gad1, 5'‐TCCAGTGCTCTGCCATTCTG ‐3' /5'‐CATAGGAGACGTCATACTGCTTGTC‐3'; for mouse Gad2, 5'‐ATGGGTGTCCCCTTGCAGT‐3' /5'‐ACCCAGTAGTCCCCTTTGCTC‐3'; and for mouse Th, 5'‐CCAATACAAGCAGGGTGAGCC‐3' /5'‐AGCATAGAGGCCCTTCAGCG ‐3'.

### 4.13 TRPA1 Knockdown

Recombinant AAV (serotype 5) carrying a short hairpin RNA (shRNA) targeting TRPA1 for TRPA1‐KD or a scrambled hairpin in the MeV nucleus were produced by SignaGen (SignaGen Laboratories, Gaithersburg, MD). We designed two independent shRNAs for TRPA1 (TRPA1‐shRNA1: ACACGTGGACATCAAAGCCGTGTTC, and TRPA1‐shRNA2: GCCTTCAATTCTACTGGAA), as well as one scrambled shRNA (GGCGCGTATAGTCGCGCGTATGTC), which did not target any sequence, as described previously [83]. In brief, the expression of shRNA was driven under a U6 promoter with co‐expression of EGFP under a CMV promoter. AAV was produced with three plasmids: AAV cis, AAV trans, and adenovirus helper plasmids. Real‐time PCR (Applied Biosystems, 7900 HT) was used to determine the AAV particles titer, and the Limulus amebocyte lysate gel‐clot method was used for endotoxin assays. The final concentration of virus in PBS was ∼10^13^ viral particles/mL. TRPA1 or scrambled shRNA‐EGFP AAV5 vectors (0.2 µL) were stereotaxically injected into mice anesthetized with isoflurane. The injection was bilateral at the coordinates 5.4 mm caudal to bregma, ± 1.1 mm lateral, and 3.9 mm deep. Mice were recovered for 4 weeks before CFA injection and behavioral studies.

### 4.14 Behavioral Tests

Behavioral tests for mechanical hyperalgesia and allodynia were conducted in a double‐blind fashion as previously described [49]. Briefly, mice were habituated to the room for 30 min every day for 3 days before tests. When a mouse was motionless, a series of von Frey filaments from 0.008 to 4 g were applied to the orofacial skin on and near the masseter muscle, where the hair had been removed to avoid tactile stimulation. Each filament was applied 5 times at ∼5 s intervals. The response frequencies were determined as (number of responses/number of stimuli) × 100% and plotted against filament force as a stimulus‐response curve. The tests were started with the filament with the lowest force and ended with the filament with 100% response.

The bite force of mice was measured as previously described [84]. In short, mice were trained to bite a force transducer. Signals were delivered from the transducer to a CED 1401 and were acquired with a Thresh Pulse script, which detected the threshold for a bite force signal. The bite force data were analyzed with the Bite Analysis script for Spike 2.

To investigate the potential effects of RNAi on motor coordination, the mice performed the rotarod test on a constant‐speed device (Ugo Basile, Varese, Italy). Mice were tested on the rod for 5–10 min for two days of training. The time the mice spent on the rod without looping and falling was recorded for 6 min at 10, 18, and 30 r.p.m. 1 day before and 28 days after intra‐MeV gene transfer. A cut‐off time of 180 s was chosen.

### 4.15 Statistical Analysis

Each group of experiments was replicated at least three times. Data are presented as mean ± SEM. No statistical methods were used to pre‐determine sample sizes, but our sample sizes are consistent with those reported in similar studies. No samples or animals that provided successful measurements were excluded from analysis. Statistical comparisons were performed with the two‐tailed unpaired/paired Student's *t*‐test, Fisher's exact test, one‐way, two‐way, or three‐way ANOVA, followed by Fisher's LSD post hoc test as indicated. All data were tested for normality, and the non‐parametric Mann‐Whitney test was used for data with nonequivalent variances. Data are presented as box plots, in which the box and whisker plots show medians (central line in the box), ranges between the 25th and 75th percentiles (box), and minimum–maximum ranges (whiskers). All tests were conducted using GraphPad Prism 5.0 (GraphPad Software, CA, USA), and significant differences were accepted when *p* < 0.05. The number of cells and mice analyzed is indicated in the figure legends.

## Author Contributions

X.Z., J.Y., and X.W. performed the experiments, with the help of J.L., Y.L., X.D., R.H., J.A., J.W., and F.Z.; F.L. generated the GCaMP mice; X.Z., C.W., J.Y., X.W., F.Z., Y.G., F.W., and Z.Z. designed and analyzed all experiments. Z.Z. and F.W. supervised the project and wrote the manuscript with input from all authors.

## Funding

This work was supported by grants from the the National Natural Science Foundation of China (31330024, 32525031, 31761133016, 21790390, 31327901, 32171233, 31521062, 31670843, 31600950, and 21790394); the National Basic Research Program of China (2012CB518006); Clinical Medicine Plus X‐Young Scholars Project, Peking University, the Fundamental Research Funds for the Central Universities (PKU2022LCXQ024); the Natural Science Foundation of Shaanxi Province of China (2023‐ZDLSF‐23, 2021TD‐37, and 2019JC‐07); the Sanya Science and Technology Special Fund (2024KJFX084); the Sanqin Talent Special Support Program (SYLTD‐S02); the NIH/NINDS (NS 091296 and DE024220); the Neuroscience Center, the Chinese Academy of Medical Sciences, and the Chinese Human Brain Banking Consortium.

## Conflicts of Interest

The authors declare no conflicts of interest.

## Supporting information




**Supporting file**: advs75113‐sup‐0001‐SuppMat.docx

## Data Availability

Data available on request from the authors.

## References

[1] H. Flor , M. M. Schugens , and N. Birbaumer , “Discrimination of Muscle Tension in Chronic Pain Patients and Healthy Controls,” Biofeedback and Self‐Regulation 17 (1992): 165–177, 10.1007/BF01000401. 1387553

[2] M. Hollins , A. Sigurdsson , L. Fillingim , and A. K. Goble , “Vibrotactile Threshold Is Elevated in Temporomandibular Disorders,” Pain 67 (1996): 89–96, 10.1016/0304-3959(96)03083-7. 8895235

[3] N. E. Lazarov , “Neurobiology of Orofacial Proprioception,” Brain Research Reviews 56 (2007): 362–383, 10.1016/j.brainresrev.2007.08.009. 17915334

[4] S. Mense , “Muscle Pain: Mechanisms and Clinical Significance,” Deutsches Ärzteblatt International 105 (2008): 214–219. 19629211 10.3238/artzebl.2008.0214 PMC2696782

[5] B. Gerdle , B. Ghafouri , M. Ernberg , and B. Larsson , “Chronic Musculoskeletal Pain: Review of Mechanisms and Biochemical Biomarkers as Assessed by the Microdialysis Technique,” Journal of Pain Research 7 (2014): 313–326, 10.2147/JPR.S59144. 24966693 PMC4062547

[6] A. Biasiotta , A. Peddireddy , K. Wang , et al., “Effect of Pinching‐evoked Pain on Jaw‐stretch Reflexes and Exteroceptive Suppression Periods in Healthy Subjects,” Clinical Neurophysiology 118 (2007): 2180–2188, 10.1016/j.clinph.2007.07.002. 17714986

[7] M. K. A. van Selms , K. Wang , F. Lobbezoo , P. Svensson , L. Arendt‐Nielsen , and M. Naeije , “Effects of Masticatory Muscle Fatigue Without and With Experimental Pain on Jaw‐stretch Reflexes in Healthy Men and Women,” Clinical Neurophysiology 116 (2005): 1415–1423, 10.1016/j.clinph.2005.02.017. 15978504

[8] J. Lin , M. Halaki , P. Rajan , and A. Leaver , “Relationship Between Proprioception and Pain and Disability in People With Non‐Specific Low Back Pain: A Systematic Review With Meta‐Analysis,” Spine 44 (2019): E606–E617. 30726200 10.1097/BRS.0000000000002917

[9] P. Svensson , K. Wang , L. Arendt‐Nielsen , and B. E. Cairns , “Effects of NGF‐induced Muscle Sensitization on Proprioception and Nociception,” Experimental Brain Research 189 (2008): 1–10, 10.1007/s00221-008-1399-4. 18478213

[10] A. M. Fuller , A. Luiz , N. Tian , et al., “Gate Control of Sensory Neurotransmission in Peripheral Ganglia by Proprioceptive Sensory Neurons,” Brain 146 (2023): 4033–4039, 10.1093/brain/awad182. 37249190 PMC10549771

[11] N. E. Lazarov , “Comparative Analysis of the Chemical Neuroanatomy of the Mammalian Trigeminal Ganglion and Mesencephalic Trigeminal Nucleus,” Progress in Neurobiology 66 (2002): 19–59, 10.1016/S0301-0082(01)00021-1. 11897404

[12] K. D. Veerapaneni , N. Kapoor , P. Veerapaneni , F. Lui, and K. Nalleballe , “Trigeminal Neuropathy,” StatPearls (StatPearls Publishing, 2024). 32310586

[13] J. P. Lund , S. Sadeghi , T. Athanassiadis , et al., “Assessment of the Potential Role of Muscle Spindle Mechanoreceptor Afferents in Chronic Muscle Pain in the Rat Masseter Muscle,” PLoS ONE 5 (2010): 11131, 10.1371/journal.pone.0011131. PMC2886111 20559566

[14] R. Masri , J. Y. Ro , and N. Capra , “The Effect of Experimental Muscle Pain on the Amplitude and Velocity Sensitivity of Jaw Closing Muscle Spindle Afferents,” Brain Research 1050 (2005): 138–147, 10.1016/j.brainres.2005.05.039. 15982645

[15] B. Zhang , X. Y. Zhang , P. F. Luo , et al., “Action potential‐triggered somatic exocytosis in mesencephalic trigeminal nucleus neurons in rat brain slices,” The Journal of physiology 590 (2012): 753–762, 10.1113/jphysiol.2011.221051. 22124145 PMC3381308

[16] L. Vargova and E. Sykova , “Astrocytes and Extracellular Matrix in Extrasynaptic Volume Transmission,” Philosophical Transactions of the Royal Society B: Biological Sciences 369 (2014): 20130608, 10.1098/rstb.2013.0608. PMC4173293 25225101

[17] K. H. Taber and R. A. Hurley , “Volume Transmission in the Brain: Beyond the Synapse,” The Journal of Neuropsychiatry and Clinical Neurosciences 26 (2014): 4, 10.1176/appi.neuropsych.13110351. 24515717

[18] R. Huang , Y. Wang , J. Li , et al., “Ca^2+^‐Independent But Voltage‐Dependent Quantal Catecholamine Secretion (CiVDS) in the Mammalian Sympathetic Nervous System,” Proceedings of the National Academy of Sciences USA 116 (2019): 20201–20209, 10.1073/pnas.1902444116. PMC6778234 31530723

[19] C. Zhang and Z. Zhou , “Ca^2+^‐Independent But Voltage‐Dependent Secretion In Mammalian Dorsal Root Ganglion Neurons,” Nature Neuroscience 5 (2002) 425–430, 10.1038/nn845. 11953753

[20] H. P. Huang , S. R. Wang , W. Yao , et al., “Long Latency of Evoked Quantal Transmitter Release From Somata of Locus Coeruleus Neurons in Rat Pontine Slices,” Proceedings of the National Academy of Sciences 104 (2007): 1401–1406, 10.1073/pnas.0608897104. PMC1783087 17227848

[21] G. M. Story , A. M. Peier , A. J. Reeve , et al., “ANKTM1, a TRP‐Like Channel Expressed in Nociceptive Neurons, Is Activated by Cold Temperatures,” Cell 112 (2003): 819–829, 10.1016/S0092-8674(03)00158-2. 12654248

[22] S. E. Jordt , D. M. Bautista , H. H. Chuang , et al., “Mustard Oils and Cannabinoids Excite Sensory Nerve Fibres Through the TRP Channel ANKTM1,” Nature 427 (2004): 260–265, 10.1038/nature02282. 14712238

[23] D. M. Bautista , S. E. Jordt , T. Nikai , et al., “TRPA1 Mediates the Inflammatory Actions of Environmental Irritants and Proalgesic Agents,” Cell 124 (2006): 1269–1282, 10.1016/j.cell.2006.02.023. 16564016

[24] B. Nilius , G. Owsianik , T. Voets , and J. A. Peters , “Transient Receptor Potential Cation Channels in Disease,” Physiological Reviews 87 (2007): 165–217, 10.1152/physrev.00021.2006. 17237345

[25] D. M. Bautista , M. Pellegrino , and M. Tsunozaki , “TRPA1: A Gatekeeper for Inflammation,” Annual Review of Physiology 75 (2013): 181–200, 10.1146/annurev-physiol-030212-183811. PMC4041114 23020579

[26] M. Connor , L. A. Naves , and E. W. McCleskey , “Contrasting Phenotypes of Putative Proprioceptive and Nociceptive Trigeminal Neurons Innervating Jaw Muscle in Rat,” Molecular Pain 1 (2005): 31, 10.1186/1744-8069-1-31. 16242047 PMC1283980

[27] S. M. Brierley , J. Castro , A. M. Harrington , et al., “TRPA1 contributes to Specific Mechanically Activated Currents and Sensory Neuron Mechanical Hypersensitivity,” The Journal of Physiology 589 (2011): 3575–3593, 10.1113/jphysiol.2011.206789. 21558163 PMC3167119

[28] K. Y. Kwan , J. M. Glazer , D. P. Corey , F. L. Rice , and C. L. Stucky , “TRPA1 Modulates Mechanotransduction in Cutaneous Sensory Neurons,” The Journal of Neuroscience 29 (2009): 4808–4819, 10.1523/JNEUROSCI.5380-08.2009. 19369549 PMC2744291

[29] W. L. Shen , Y. Kwon , A. A. Adegbola , J. Luo , A. Chess , and C. Montell , “Function of Rhodopsin in Temperature Discrimination in Drosophila,” Science 331 (2011): 1333–1336, 10.1126/science.1198904. 21393546

[30] S. Shang , F. Zhu , B. Liu , et al., “Intracellular TRPA1 Mediates Ca^2+^ Release From Lysosomes in Dorsal Root Ganglion Neurons,” Journal of Cell Biology 215 (2016): 369–381, 10.1083/jcb.201603081. 27799370 PMC5100290

[31] D. Florez‐Paz , K. K. Bali , R. Kuner , and A. Gomis , “A Critical Role for Piezo2 Channels in the Mechanotransduction of Mouse Proprioceptive Neurons,” Scientific Reports 6 (2016): 25923, 10.1038/srep25923. 27184818 PMC4869095

[32] S. R. Garrison and C. L. Stucky , “Contribution of Transient Receptor Potential Ankyrin 1 to Chronic Pain in Aged Mice With Complete Freund's Adjuvant–Induced Arthritis,” Arthritis & rheumatology 66 (2014): 2380–2390, 10.1002/art.38724. 24891324 PMC4149259

[33] J. Asgar , Y. Zhang , J. L. Saloman , S. Wang , M. K. Chung , and J. Y. Ro , “The Role of TRPA1 in Muscle Pain and Mechanical Hypersensitivity Under Inflammatory Conditions in Rats,” Neuroscience 310 (2015): 206–215, 10.1016/j.neuroscience.2015.09.042. 26393428 PMC4633371

[34] J. Akerboom , T. W. Chen , T. J. Wardill , et al., “Optimization of a GCaMP Calcium Indicator for Neural Activity Imaging,” The Journal of Neuroscience 32 (2012): 13819–13840, 10.1523/JNEUROSCI.2601-12.2012. 23035093 PMC3482105

[35] S. Wang , J. Lim , J. Joseph , et al., “Spontaneous and Bite‐Evoked Muscle Pain Are Mediated by a Common Nociceptive Pathway with Differential Contribution by TRPV1,” The Journal of Pain 18 (2017): 1333–1345, 10.1016/j.jpain.2017.06.005. 28669862 PMC5660653

[36] C. P. Fernandes , P. O. Glantz , S. A. Svensson , and A. Bergmark , “A Novel Sensor for Bite Force Determinations,” Dental Materials 19 (2003): 118–126, 10.1016/S0109-5641(02)00020-9. 12543117

[37] J. Y. Ro , “Bite Force Measurement in Awake Rats: A Behavioral Model for Persistent Orofacial Muscle Pain and Hyperalgesia,” Journal of Orofacial Pain 19 (2005): 159–167. 15895839

[38] C. Zhang , W. Xiong , H. Zheng , L. Wang , B. Lu , and Z. Zhou , “Calcium‐ and Dynamin‐independent Endocytosis in Dorsal Root Ganglion Neurons,” Neuron 42 (2004): 225–236, 10.1016/S0896-6273(04)00189-8. 15091339

[39] Z. Chai , C. Wang , R. Huang , et al., “CaV2.2 Gates Calcium‐Independent but Voltage‐Dependent Secretion in Mammalian Sensory Neurons,” Neuron 96 (2017) 1317–1326, 10.1016/j.neuron.2017.10.028. 29198756

[40] Y. Karashima , J. Prenen , K. Talavera , A. Janssens , T. Voets , and B. Nilius , “Agonist‐Induced Changes in Ca^2+^ Permeation Through the Nociceptor Cation Channel TRPA1,” Biophysical Journal 98 (2010): 773–783, 10.1016/j.bpj.2009.11.007. 20197030 PMC2830466

[41] R. Schneggenburger , Z. Zhou , A. Konnerth , and E. Neher , “Fractional Contribution of Calcium to the Cation Current Through Glutamate Receptor Channels,” Neuron 11 (1993): 133–143, 10.1016/0896-6273(93)90277-X. 7687849

[42] A. Pertovaara , “Noradrenergic Pain Modulation,” Progress in Neurobiology 80 (2006): 53–83, 10.1016/j.pneurobio.2006.08.001. 17030082

[43] S. Hirschberg , Y. Li , A. Randall , E. J. Kremer , and A. E. Pickering , “Functional Dichotomy in Spinal‐ vs Prefrontal‐projecting Locus Coeruleus Modules Splits Descending Noradrenergic Analgesia From Ascending Aversion and Anxiety in Rats,” eLife 6 (2017): 29808. 10.7554/eLife.29808 PMC5653237 29027903

[44] V. Breton‐Provencher and M. Sur , “Active Control of Arousal by a Locus Coeruleus GABAergic Circuit,” Nature Neuroscience 22 (2019): 218–228, 10.1038/s41593-018-0305-z. 30643295 PMC6385895

[45] H. Taniguchi , M. He , P. Wu , et al., “A Resource of Cre Driver Lines for Genetic Targeting of GABAergic Neurons in Cerebral Cortex,” Neuron 71 (2011): 995–1013, 10.1016/j.neuron.2011.07.026. 21943598 PMC3779648

[46] J. E. Turman Jr. and S. H. Chandler , “Immunohistochemical Localization of Glutamate and Glutaminase in guinea Pig Trigeminal Premotoneurons,” Brain Research 634 (1994): 49–61, 10.1016/0006-8993(94)90257-7. 7512428

[47] E. Kheradpezhouh , J. M. C. Choy , V. R. Daria , and E. Arabzadeh , “TRPA1 expression and Its Functional Activation in Rodent Cortex,” Open Biology 7 (2017): 160314, 10.1098/rsob.160314. 28424320 PMC5413904

[48] H. Doihara , K. Nozawa , E. Kawabata‐Shoda , R. Kojima , T. Yokoyama , and H. Ito , “Molecular Cloning and Characterization of Dog TRPA1 and AITC Stimulate the Gastrointestinal Motility Through TRPA1 in Conscious Dogs,” European Journal of Pharmacology 617 (2009): 124–129, 10.1016/j.ejphar.2009.06.038. 19576208

[49] Y. S. Kim , Y. Chu , L. Han , et al., “Central Terminal Sensitization of TRPV1 by Descending Serotonergic Facilitation Modulates Chronic Pain,” Neuron 81 (2014): 873–887, 10.1016/j.neuron.2013.12.011. 24462040 PMC3943838

[50] B. Zhang , X. Y. Zhang , P. F. Luo , et al., “Action potential–triggered somatic exocytosis mesencephalic trigeminal nucleus neurons in rat brain slices,” The Journal of Physiology 590 (2012): 753–762, 10.1113/jphysiol.2011.221051. 22124145 PMC3381308

[51] Z. P. Pang and T. C. Sudhof , “Cell Biology of Ca2+‐triggered Exocytosis,” Current Opinion in Cell Biology 22 (2010): 496–505, 10.1016/j.ceb.2010.05.001. 20561775 PMC2963628

[52] S. S. Stojilkovic , “Ca^2+^‐Regulated Exocytosis and SNARE Function,” Trends in Endocrinology & Metabolism 16 (2005): 81–83, 10.1016/j.tem.2005.02.002. 15808803

[53] A. T. Brunger , U. B. Choi , Y. Lai , J. Leitz , and Q. Zhou , “Molecular Mechanisms of Fast Neurotransmitter Release,” Annual Review of Biophysics 47 (2018): 469–497, 10.1146/annurev-biophys-070816-034117. PMC6378885 29792815

[54] C. Wang , X. Kang , L. Zhou , et al., “Synaptotagmin‐11 Is a Critical Mediator of Parkin‐linked Neurotoxicity and Parkinson's Disease‐Like Pathology,” Nature Communications 9 (2018): 81, 10.1038/s41467-017-02593-y. PMC5758517 29311685

[55] X. Wu , J. Yao , J. Huo , et al., “Calcium‐Sensitive Synaptotagmin 11‐Lipid Interaction Modulates Exo‐Endocytosis,” Nature Communications 17 (2025): 685, 10.1038/s41467-025-67320-4. PMC12819558 41402317

[56] Q. Zhang , B. Liu , Q. Wu , et al., “Differential Co‐release of Two Neurotransmitters from a Vesicle Fusion Pore in Mammalian Adrenal Chromaffin Cells,” Neuron 102 (2019): 173–183. 30773347 10.1016/j.neuron.2019.01.031

[57] W. Guo , S. Zou , Z. Mohammad , et al., “Voluntary Biting Behavior as a Functional Measure of Orofacial Pain in Mice,” Physiology & Behavior 204 (2019): 129–139, 10.1016/j.physbeh.2019.02.024. 30797813 PMC6475465

[58] E. M. Kogawa , P. S. Calderon , J. R. Lauris , C. R. Araujo , and P. C. Conti , “Evaluation of Maximal Bite Force in Temporomandibular Disorders Patients,” Journal of Oral Rehabilitation 33 (2006): 559–565, 10.1111/j.1365-2842.2006.01619.x. 16856953

[59] J. W. Kleinfelder and K. Ludwigt , “Maximal Bite Force in Patients With Reduced Periodontal Tissue Support With and Without Splinting,” Journal of Periodontology 73 (2002): 1184–1187, 10.1902/jop.2002.73.10.1184. 12416777

[60] S. H. Woo , V. Lukacs , J. C. de Nooij , et al., “Piezo2 is the Principal Mechanotransduction Channel for Proprioception,” Nature Neuroscience 18 (2015): 1756–1762, 10.1038/nn.4162. 26551544 PMC4661126

[61] M. A. Efstathiou , C. D. Giannaki , Z. Roupa , S. Hadjisavvas , and M. Stefanakis , “Evidence of Distorted Proprioception and Postural Control in Studies of Experimentally Induced Pain: A Critical Review of the Literature,” Scandinavian Journal of Pain 22 (2022): 445–456, 10.1515/sjpain-2021-0205. 35470647

[62] E. Frayne , S. Coulson , R. Adams , G. Croxson , and G. Waddington , “Laterality of Proprioception in the Orofacial Muscles and Temporomandibular Joint,” Neuroscience Letters 635 (2016): 111–116, 10.1016/j.neulet.2016.10.030. 27771297

[63] T. Takahashi , M. Shirasu , M. Shirasu , et al., “The Locus Coeruleus Projects To The Mesencephalic Trigeminal Nucleus In Rats,” Neuroscience Research 68 (2010): 103–106. 20599446 10.1016/j.neures.2010.06.012

[64] D. Verdier , J. P. Lund , and A. Kolta , “Synaptic Inputs to Trigeminal Primary Afferent Neurons Cause Firing and Modulate Intrinsic Oscillatory Activity,” Journal of Neurophysiology 92 (2004): 2444–2455, 10.1152/jn.00279.2004. 15381749

[65] M. J. Millan , “Descending Control of Pain,” Progress in Neurobiology 66 (2002): 355–474, 10.1016/S0301-0082(02)00009-6. 12034378

[66] M. Tsuruoka , K. Matsutani , and T. Inoue , “Coeruleospinal Inhibition of Nociceptive Processing in the Dorsal Horn During Unilateral Hindpaw Inflammation in the Rat,” Pain 104 (2003): 353–361, 10.1016/S0304-3959(03)00042-3. 12855345

[67] I. Suarez‐Pereira , M. Llorca‐Torralba , L. Bravo , C. Camarena‐Delgado , C. Soriano‐Mas , and E. Berrocoso , “The Role of the Locus Coeruleus in Pain and Associated Stress‐Related Disorders,” Biological Psychiatry 91 (2022): 786–797, 10.1016/j.biopsych.2021.11.023. 35164940

[68] M. Llorca‐Torralba , C. Camarena‐Delgado , I. Suarez‐Pereira , et al., “Pain and Depression Comorbidity Causes Asymmetric Plasticity in the Locus Coeruleus Neurons,” Brain 145 (2022): 154–167, 10.1093/brain/awab239. 34373893 PMC8967092

[69] B. Donertas‐Ayaz and R. M. Caudle , “Locus Coeruleus‐noradrenergic Modulation of Trigeminal Pain: Implications for Trigeminal Neuralgia and Psychiatric Comorbidities,” Neurobiology of Pain 13 (2023): 100124, 10.1016/j.ynpai.2023.100124. 36974102 PMC10038791

[70] P. W. Howorth , S. R. Thornton , V. O'Brien , et al., “Retrograde Viral Vector‐mediated Inhibition of Pontospinal Noradrenergic Neurons Causes Hyperalgesia in Rats,” The Journal of Neuroscience 29 (2009): 12855–12864, 10.1523/JNEUROSCI.1699-09.2009. 19828800 PMC2777275

[71] F. Wei , R. Dubner , and K. Ren , “Nucleus Reticularis Gigantocellularis and Nucleus Raphe Magnus in the Brain Stem Exert Opposite Effects on Behavioral Hyperalgesia and Spinal Fos Protein Expression After Peripheral Inflammation,” Pain 80 (1999): 127–141, 10.1016/S0304-3959(98)00212-7. 10204725

[72] S. Nag and S. S. Mokha , “Activation of the Trigeminal α 2 ‐Adrenoceptor Produces Sex‐Specific, Estrogen Dependent Thermal Antinociception And Antihyperalgesia Using An Operant Pain Assay In The Rat,” Behavioural Brain Research 314 (2016): 152–158, 10.1016/j.bbr.2016.08.012. 27506651 PMC4996728

[73] M. Tsuruoka , K. Matsutani , M. Maeda , and T. Inoue , “Coeruleotrigeminal Inhibition of Nociceptive Processing in the Rat Trigeminal Subnucleus Caudalis,” Brain Research 993 (2003): 146–153, 10.1016/j.brainres.2003.09.023. 14642840

[74] S. Y. Yoon , S. Y. Kang , H. W. Kim , H. C. Kim , and D. H. Roh , “Clonidine Reduces Nociceptive Responses in Mouse Orofacial Formalin Model: Potentiation by Sigma‐1 Receptor Antagonist BD1047 without Impaired Motor Coordination,” Biological and Pharmaceutical Bulletin 38 (2015): 1320–1327. 26328487 10.1248/bpb.b15-00183

[75] X. K. Chen , L. C. Wang , Y. Zhou , et al., “Activation of GPCRs Modulates Quantal Size In Chromaffin Cells Through Gβγ and PKC,” Nature Neuroscience 8 (2005): 1160–1168, 10.1038/nn1529. 16116443

[76] E. Del‐Bel and F. F. De‐Miguel , “Extrasynaptic Neurotransmission Mediated by Exocytosis and Diffusive Release of Transmitter Substances,” Frontiers in Synaptic Neuroscience 10 (2018): 13. 29937726 10.3389/fnsyn.2018.00013 PMC6003215

[77] X. W. Chen , Y. Mu , H. P. Huang , et al., “Hypocretin‐1 Potentiates NMDA Receptor‐mediated Somatodendritic Secretion From Locus Ceruleus Neurons,” The Journal of Neuroscience 28 (2008): 3202–3208, 10.1523/JNEUROSCI.4426-07.2008. 18354023 PMC6670716

[78] B. Rawal , V. Rancic , and K. Ballanyi , “NMDA Enhances and Glutamate Attenuates Synchrony of Spontaneous Phase‐Locked Locus Coeruleus Network Rhythm in Newborn Rat Brain Slices,” Brain Sciences 12 (2022): 651, 10.3390/brainsci12050651. 35625039 PMC9140167

[79] T. Zamalloa , C. P. Bailey , and J. Pineda , “Glutamate‐Induced Post‐Activation Inhibition Of Locus Coeruleus Neurons Is Mediated by AMPA/Kainate Receptors And Sodium‐Dependent Potassium Currents,” British Journal of Pharmacology 156 (2009): 649–661, 10.1111/j.1476-5381.2008.00004.x. 19226256 PMC2697705

[80] I. Hong , J. Kim , T. Hainmueller , et al., “Calcium‐Permeable AMPA Receptors Govern PV Neuron Feature Selectivity,” Nature 635 (2024): 398–405, 10.1038/s41586-024-08027-2. 39358515 PMC11560848

[81] L. Wang , S. Shang , X. Kang , et al., “Modulation of Dopamine Release in the Striatum by Physiologically Relevant Levels of Nicotine,” Nature Communications 5 (2014): 3925, 10.1038/ncomms4925. 24968237

[82] W. Wang , H. Fang , L. Groom , et al., “Superoxide Flashes In Single Mitochondria,” Cell 134 (2008): 279–290. 18662543 10.1016/j.cell.2008.06.017 PMC2547996

[83] Y. Xiong , S. Teng , L. Zheng , et al., “Stretch‐Induced Ca(2+) Independent ATP Release in Hippocampal Astrocytes,” The Journal of Physiology 596 (2018): 1931–1947. 29488635 10.1113/JP275805 PMC5978314

[84] M. Nies and J. Y. Ro , “Bite Force Measurement in Awake Rats,” Brain Research Protocols 12 (2004): 180–185, 10.1016/j.brainresprot.2003.11.003. 15013469

