# Supplementary material for: A Central Somatic Transmission Mediates Proprioceptive Facilitation of Muscle Pain
Source: Adv Sci (Weinh). 2026 Apr 23;13(34):e14242. doi: 10.1002/advs.202514242 (PMC13285158; doi:10.1002/advs.202514242)
Supplement: Supplementary file 1 — Supporting file: advs75113‐sup‐0001‐SuppMat.docx [file ADVS-13-e14242-s001.docx]

A central somatic transmission mediates proprioceptive facilitation of muscle pain

Xiaoyu Zhang^1,2,3,†^, Jiale Yang^2,†^, Xi Wu^1,†^, Jie Li^1^, Fujian Lu^1,4^, Yiman Li^1^, Xingyu Du^1^, Rong Huang^1^, Jamila Asgar^2^, Jing Wang^2^, Ke Ren^2^, Feipeng Zhu^1^, Changhe Wang^1,5,6,^*, Yehua Gan^1,^*, Feng Wei^2,^*, Zhuan Zhou^1,^*

^1^Central Laboratory, Peking University School and Hospital of Stomatology, Institute of Molecular Medicine and Peking-Tsinghua Center for Life Sciences, State Key Laboratory of Membrane Biology and Beijing Key Laboratory of Cardiometabolic Molecular Medicine, PKU-IDG/McGovern Institute for Brain Research, Peking University, Beijing 100871, China

^2^Department of Neural and Pain Sciences, School of Dentistry; Program in Neuroscience, Center to Advance Chronic Pain Research, University of Maryland, Baltimore, MD 21201, USA

^3^Peking University School of Stomatology Sanya Division (Sanya Stomatology Center), Sanya 572022, China

^4^Department of Cardiology, Zhongshan Hospital, Institutes of Biomedical Sciences, Fudan University, Shanghai Institute of Cardiovascular Diseases, Shanghai 200032, China

^5^Department of Neurology, the Second Affiliated Hospital, Neuroscience Research Center, Key Laboratory of Biomedical Information Engineering of the Ministry of Education, School of Life Science and Technology, Xi’an Jiaotong University, Xi’an 710049, China

^6^Key Laboratory of Medical Electrophysiology, Ministry of Education of China, Collaborative Innovation Center for Prevention and Treatment of Cardiovascular Disease, and the Institute of Cardiovascular Research, Southwest Medical University, Luzhou 646000, China

^†^These authors contributed equally to this work.

*Corresponding authors. Email: [zzhou@pku.edu.cn](mailto:zzhou@pku.edu.cn) (Z.Z.); [fwei@umaryland.edu](mailto:fwei@umaryland.edu) (F.W.); [kqyehuagan@bjmu.edu.cn](mailto:kqyehuagan@bjmu.edu.cn) (Y.G.); [changhewang@xjtu.edu.cn](mailto:changhewang@xjtu.edu.cn) (C.W.)

**Address for editorial correspondence and proof:**

Dr. Zhuan Zhou

Institute of Molecular Medicine,

Peking University,

Beijing 100871, China

Tel & Fax: ++86-10-6275-3212

Email: [zzhou@pku.edu.cn](mailto:zzhou@pku.edu.cn)

**Supplementary Figures**


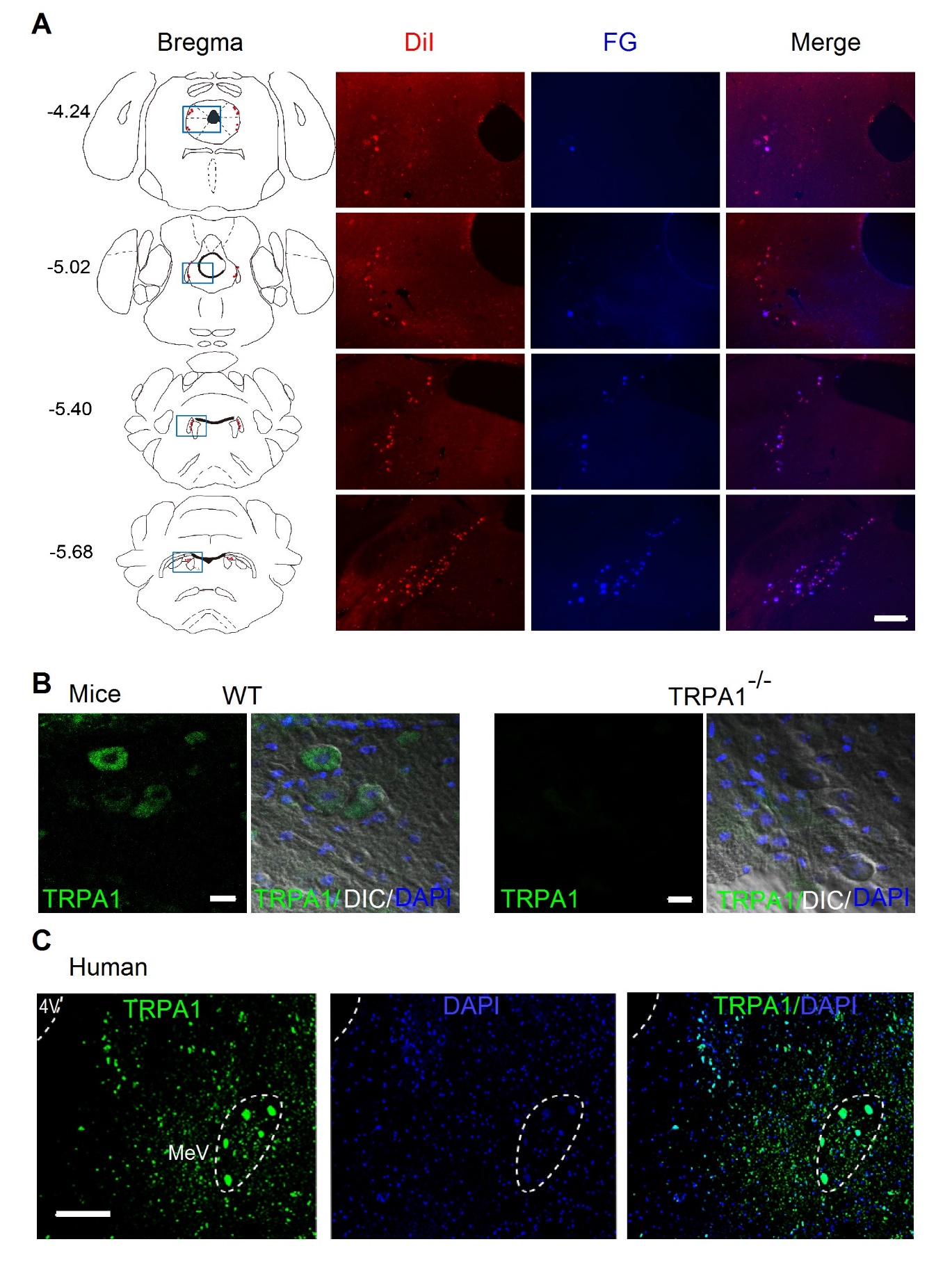


**Supplemental Figure 1. Double peripheral projections and TRPA1 expression of MeV neurons in mice.** (**A**) Representative micrographs showing retrograde labeling of the same MeV neurons with separate and same side injections of DiI into the masseter and FG into the gingiva in mice, Scale bars, 100 µm. (**B**) Immunostaining of TRPA1 in MeV neurons of WT but not in TRPA1-KO mice. DIC, differential interference contrast. Scale bars, 20 µm. (**C**) Distribution of TRPA1-immunoreactive neurons in the MeV of human brainstem section. Scale bars, 100 µm.


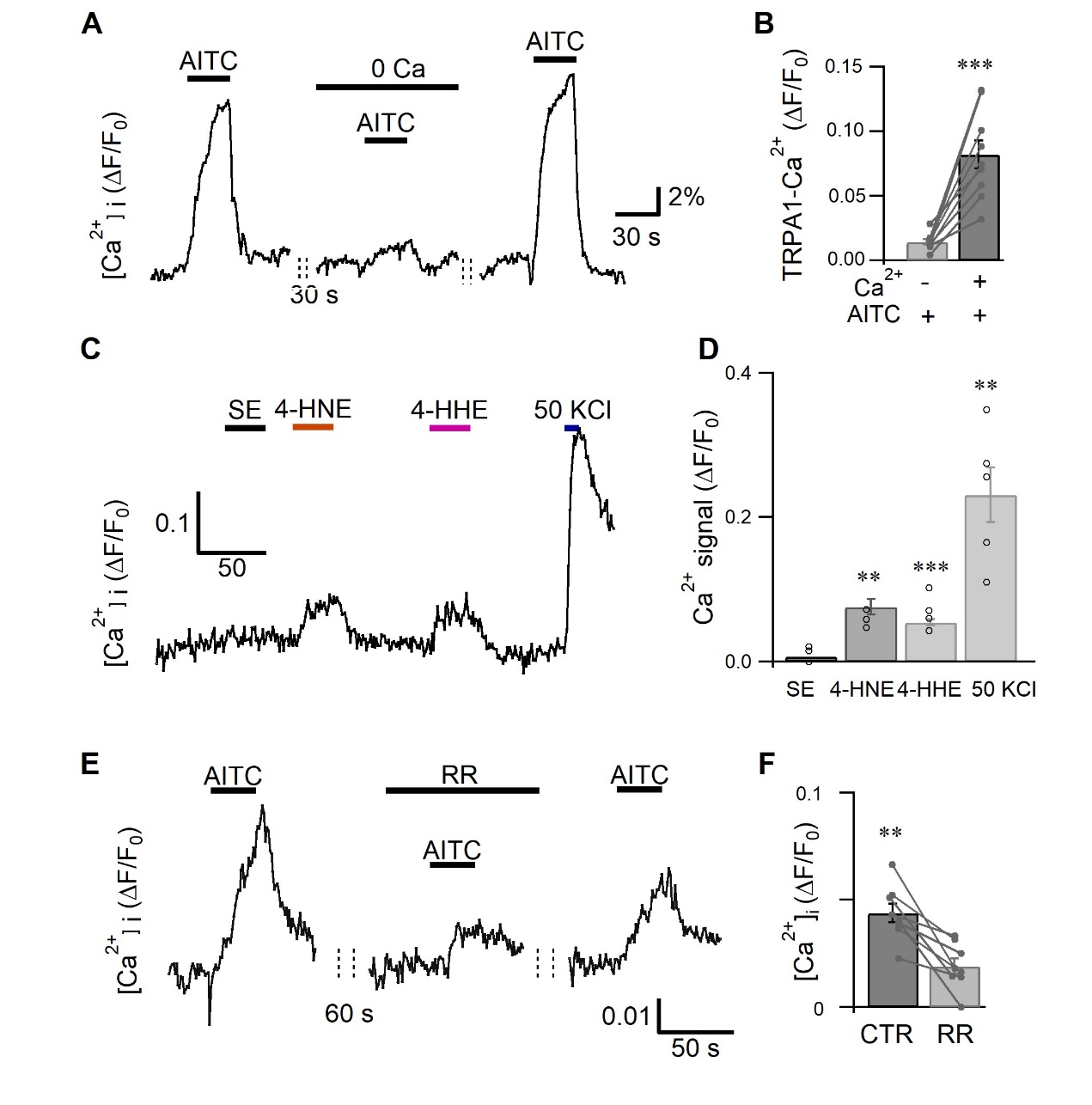


**Supplemental Figure 2. Functional TRPA1 expression in rat MeV neurons. (A**, **B**) Representative fluorometric traces (**A**) and statistics (**B**) showing typical cytosolic Ca^2+^ transients in normal external solution but not in Ca^2+^-free solution after puff-applied AITC (100 µM) in MeV slice (n = 9 cells). (**C**, **D**) Representative Ca^2+^ transients and statistics in the MeV neurons produced by the endogenous TRPA1 agonists 4-hydroxynonenal (4-HNE, 30 µM) or 4-hydroxy-2-hexenal (4-HHE, 10 µM). KCl solution (50 mM) served as a positive control, and artiﬁcial cerebrospinal ﬂuid (SE) served as a negative control (n = 5). (**E**, **F**) Representative traces and statistics showing blockade of Ca^2+^ influx induced by 100 µM AITC (control, CTR) with the nonselective blocker ruthenium red (RR, 30 µM) (n = 8). Error bars indicate SEM. ***P* < 0.01, ****P* < 0.001. Paired Student's *t*-test for (**B**, **F**), one-way ANOVA for (**D**).


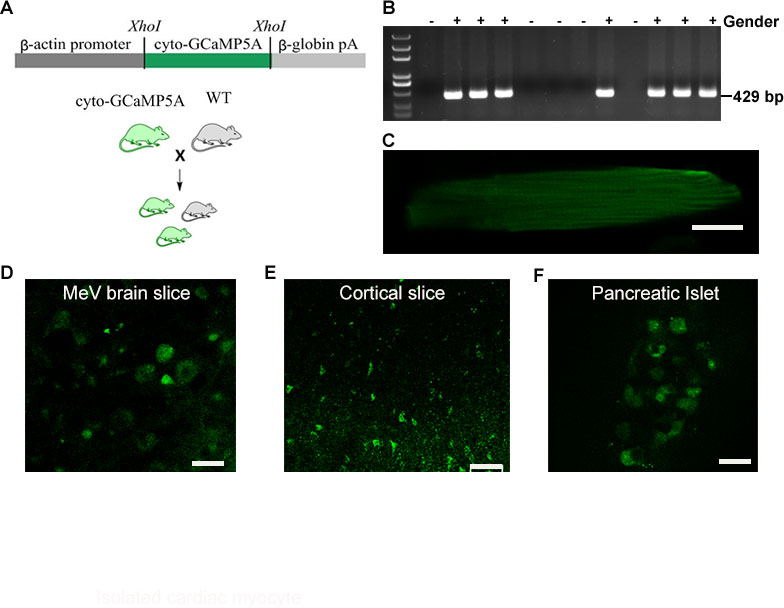


**Supplemental Figure 3. Expression of cyto-GCaMP5 in transgenic mice.** (**A**) Plasmid construct for generating the transgenic mouse. Cyto-GCaMP5A DNA was subcloned into the pUC-CAGGS expression vector downstream of the chicken β-actin promoter. Lower panel, schematic of the generation of cyto-GCaMP5 transgenic mice. (**B**) Genotyping of newborn mice was determined by PCR amplification and the PCR product of homozygous/heterozygous GCaMP5A transgenic mice was at 429 bp, with no positive band from that of wild-type mice. (**C**-**F**) Expression of GCaMP5A in cardiac myocytes (**C**), MeV brain slice (**D**), cortical brain slice (**E**), and islet cells (**F**). Scale bars, 20 µm for (**C**), 30 µm for (**E**, **F**), 50 µm for (**D**).


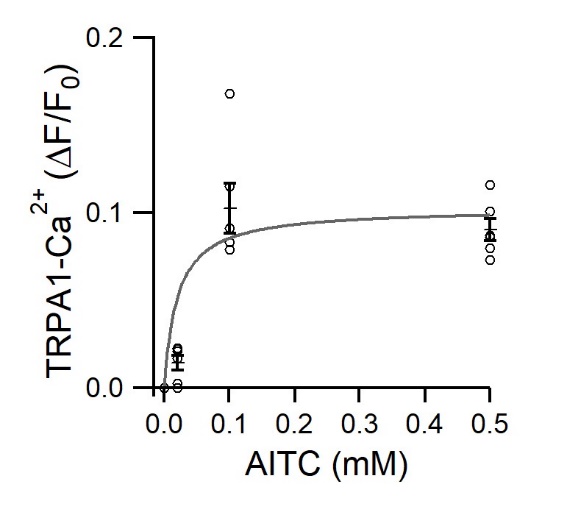


**Supplemental Figure 4.** **Dose-dependence of AITC-induced [Ca^2+^]_i_ in MeV neurons from GCaMP5 transgenic mice**. (n = 6 cells).


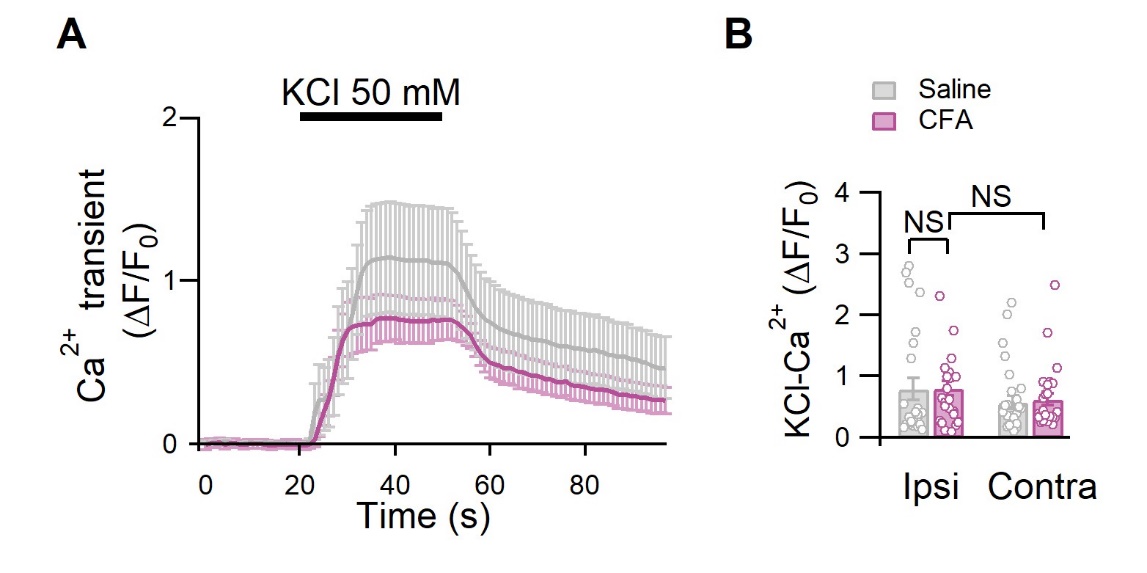


**Supplemental Figure 5.** **High-K^+^-induced Ca^2+^ raise in MeV neurons after masseter inflammation.** (**A**) Averaged time courses of [Ca^2+^]_i_ signals in response to 50 mM KCl (n = 5 cells) in ipsilateral MeV neurons at day 3 after masseteric CFA injection. (**B**) Statistics of peaks [Ca^2+^]_i_ induced by 50 mM KCl (Ipsi, n = 28 cells; Contra, n = 24 cells) in ipsilateral and contralateral MeV neurons after CFA or saline injection. Error bars indicate SEM. NS, no significance. Two-way ANOVA for (**B**).


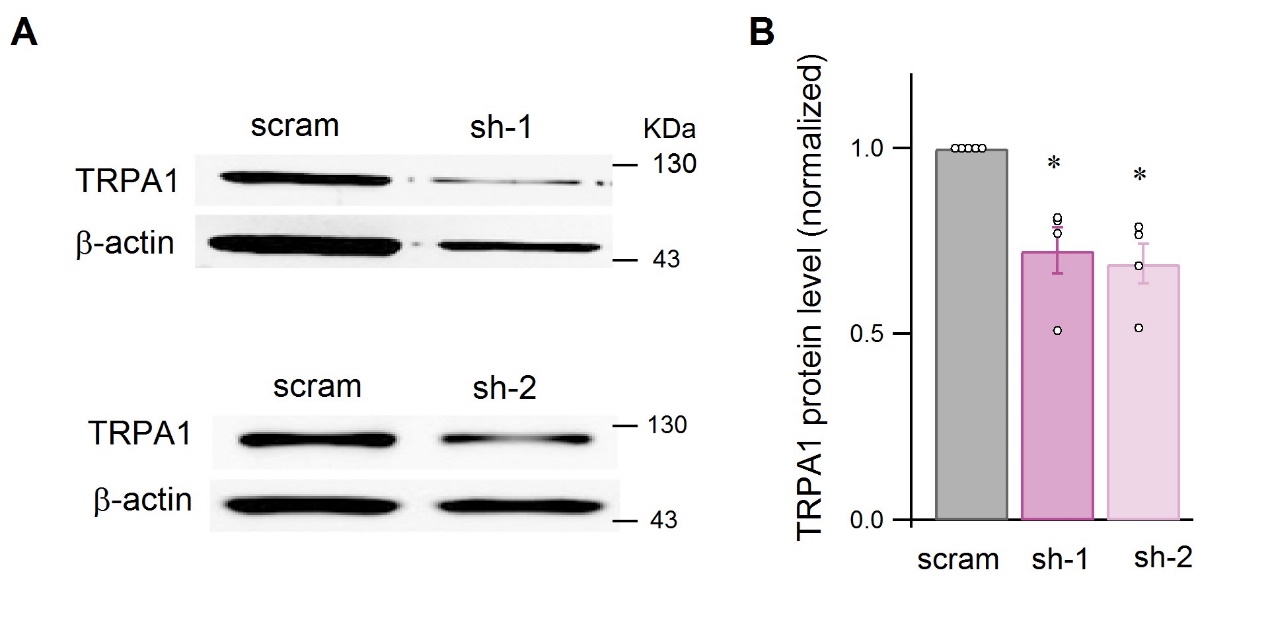


**Supplemental Figure 6.** **TRPA1 knockdown by intra-MeV shRNAs.** (**A**, **B**) Representative western blots and statistics showing TRPA1 protein levels in MeV nucleus after injection of TRPA1 shRNAs 1 (sh-1) and 2 (sh-2) *vs* scrambled shRNA (scram) (n = 4 mice/group). Error bars indicate SEM. **P* < 0.05, one-way ANOVA for (**B**).


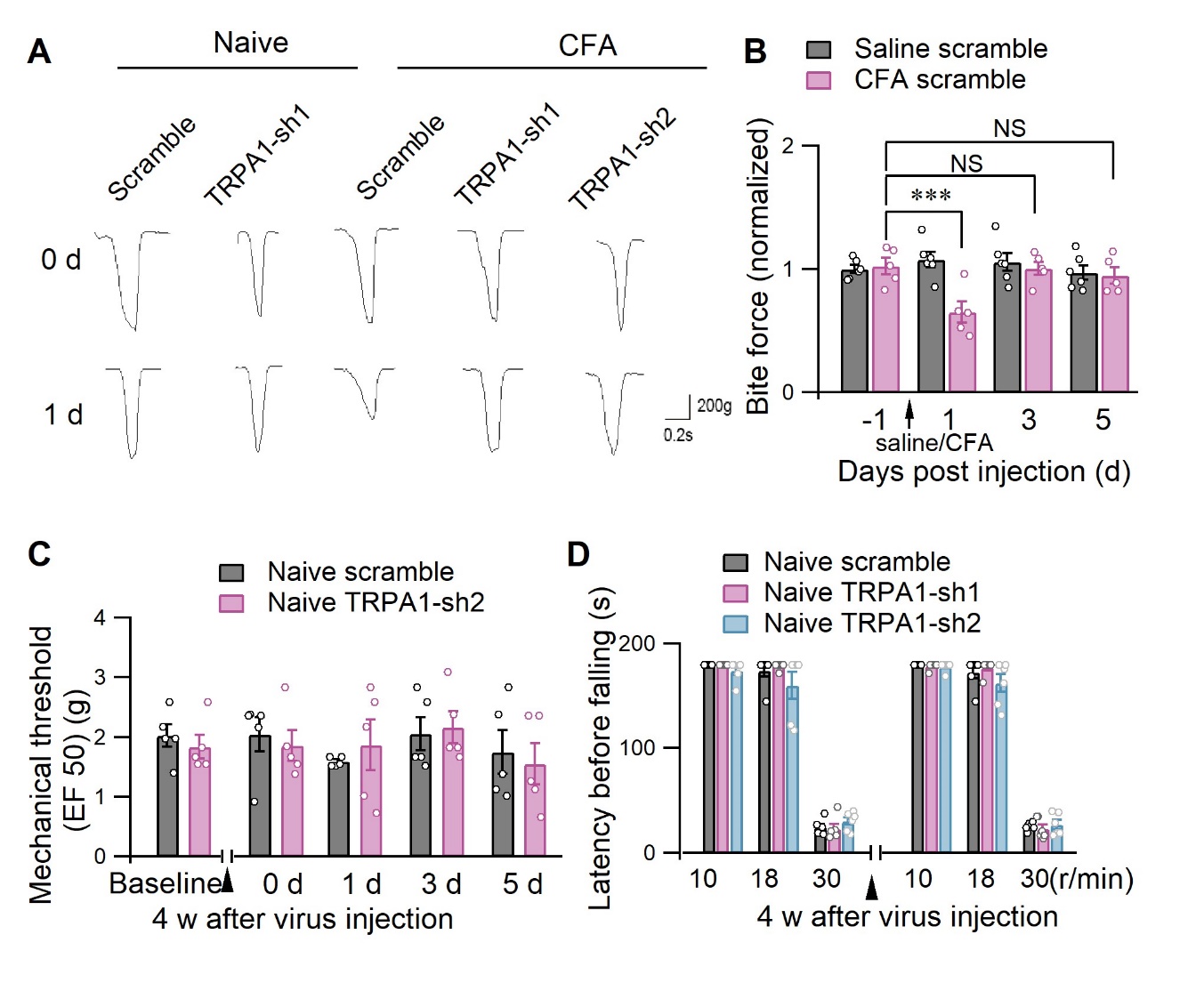


**Supplemental Figure 7. Behavioral tests of CFA-inflamed mice with TRPA1 KD in the MeV.** (**A**) Traces and statistics of bite force showing that intra-MeV treatment of TRPA1-shRNAs but not scramble shRNA prevented the bite-force reduction at 1 d after intra-masseter CFA injection. (**B**) Bite force change after scramble shRNA injection in control (saline) or CFA-treated mice (n = 5-6 mice/group). Baseline was detected 4 weeks after virus injection. (**C**) Normal mechanical threshold after scramble (scram) or TRPA1-shRNA2 injection into MeV nucleus in naïve mice (n = 5 mice/group). (**D**) Rotarod test data showing normal locomotion after scramble or TRPA1-shRNA injection (n = 6 mice/group). NS, no significance. Error bars indicate SEM. ****P* < 0.001. Two-way ANOVA for (**B**-**D**).


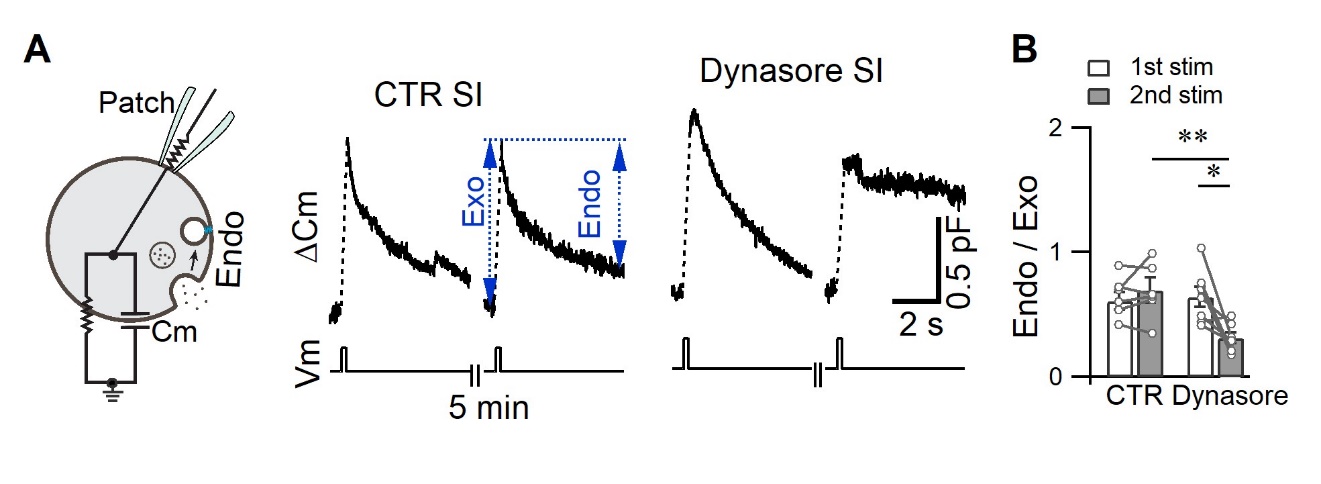


**Supplemental Figure 8.** **Dynamin-dependent endocytosis of MeV neurons**. (**A**) Left: schematic illustration of cellular capacitance measurement with patch-clamp technique to examine vesicular exocytosis (Exo) and endocytosis (Endo). Right: representative traces showing endocytosis following the depolarization-induced *C_m_* jump in response to whole-cell dialysis of dynasore (inhibitor of dynamin, 100 μM, 5 min). (**B**) Statistics of effect of dynasore on exo-endocytosis in MeV neurons as in (**A**) (n = 6 for control cells and n = 5 for dynasore-treated cells). Error bars indicate SEM. **P* < 0.05, ***P* < 0.01. Two-way ANOVA for (**B**).


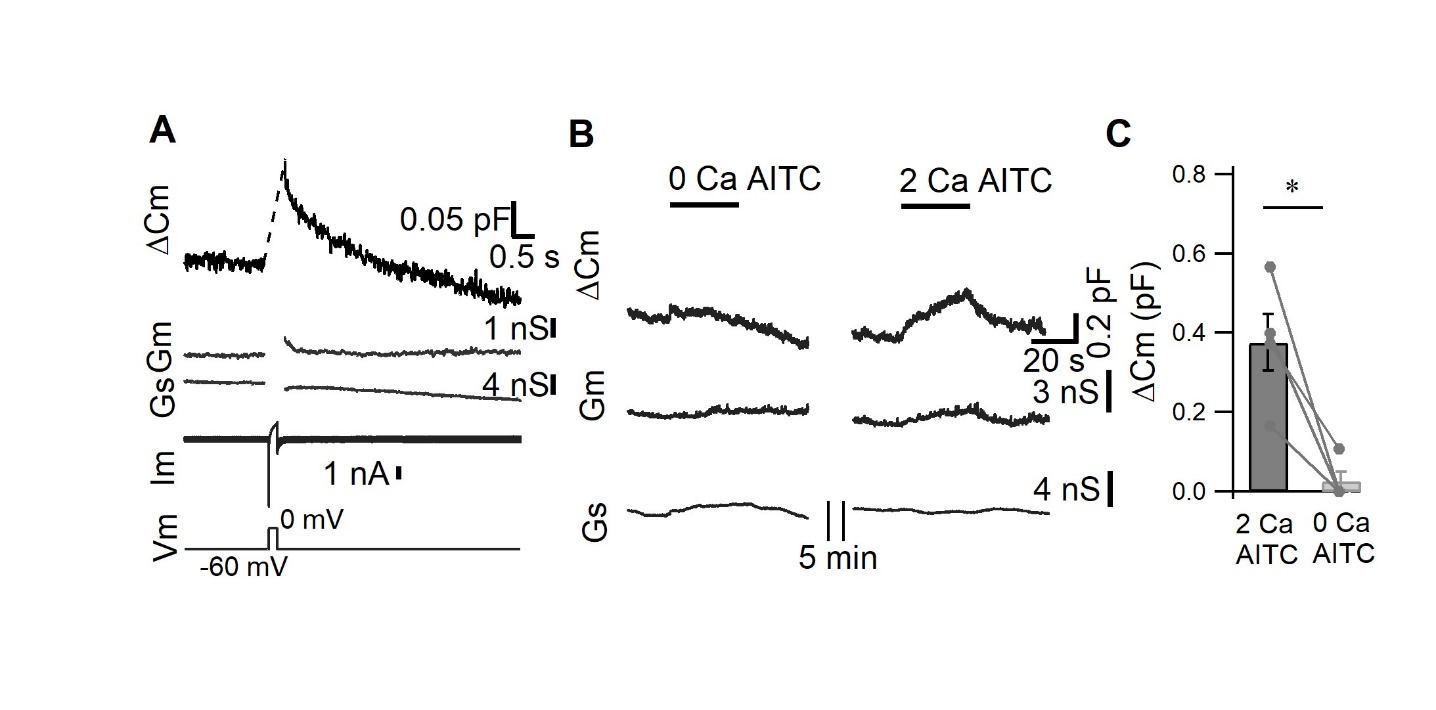


**Supplemental Figure 9. Extracellular Ca^2+^-dependence of TRPA1 activation-induced exocytosis.** (**A**) Representative exocytosis (*C*_m_) induced by 200-ms depolarization from –60 mV to 0 mV in a MeV neuron in an adult brain slice (n = 11, 0.091 ± 0.036 pF). (**B**, **C**) Typical traces and statistics showing exocytosis (*C*_m_) induced by 100 µM AITC in the presence and absence of extracellular Ca^2+^ (2 mM) (n = 4 cells per group). Error bars indicate SEM. **P* < 0.05, paired Student's *t*-test.


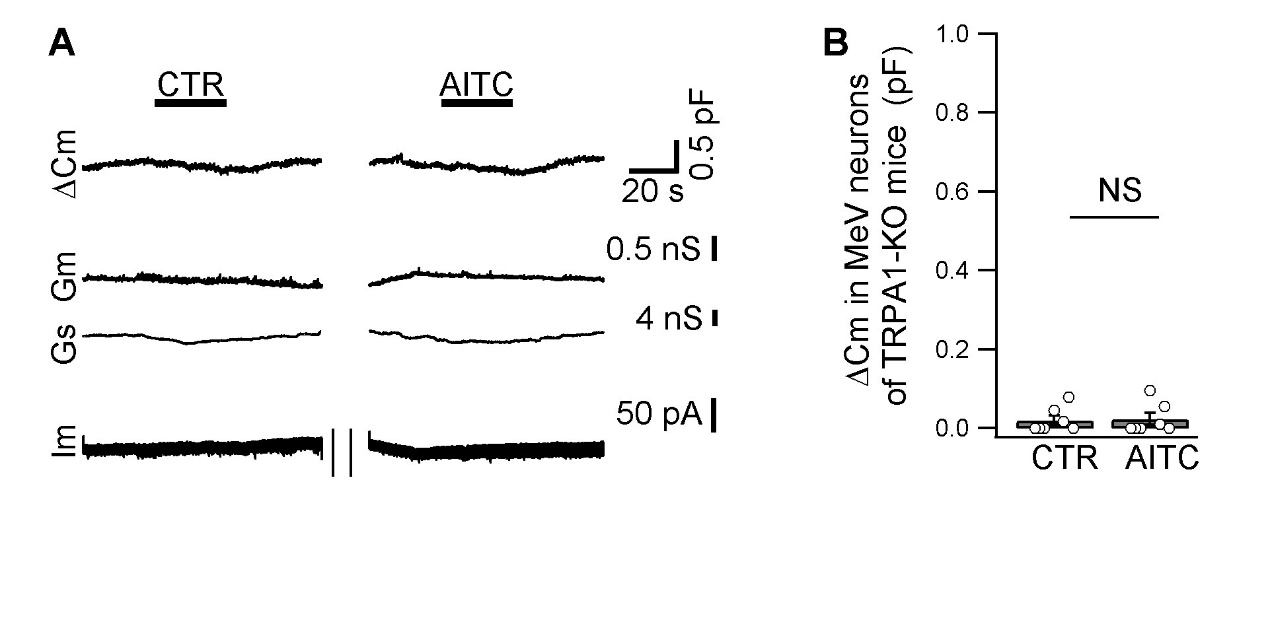


**Supplemental Figure 10.** **Cm recording in TRPA1 KO** **mice**. (**A**) Cm recording from MeV neurons in response to TRPA1 activation in brain slices of TRPA1-KO mice.(**B**) Statistics of amplitude of Cm jump as in (**A**). Normal extracellular solution was used as a negative control (CTR). n = 7 neurons per group. Error bars indicate SEM. NS, no significance. Paired Student's *t*-test for (**B**).


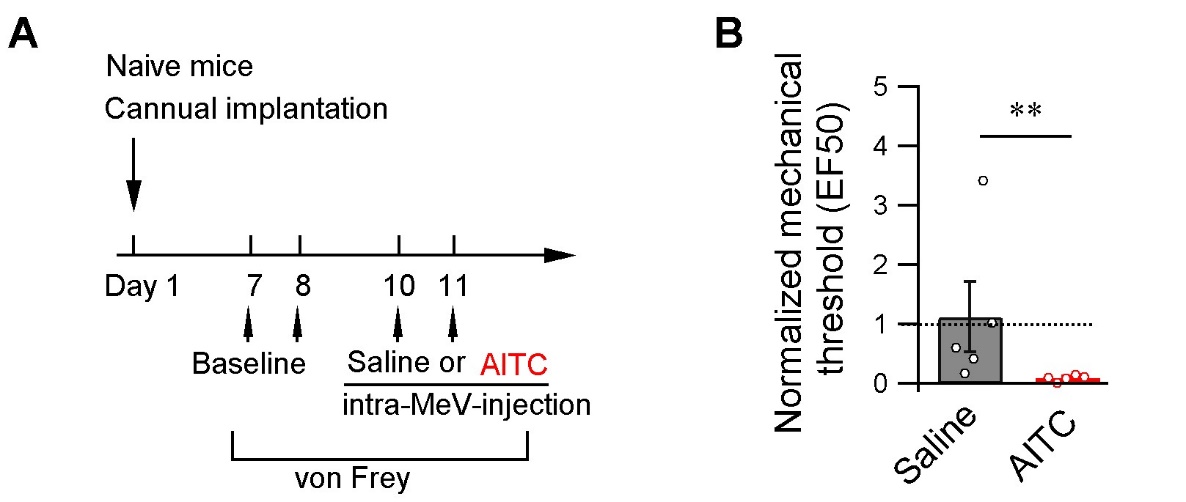


**Supplemental Figure 11.** **Effect of TRPA1 activation on mechanical threshold in naïve mice**. (**A**) Schematic showing mechanical head-withdrawal threshold measurement using von Frey filaments after microinjection of AITC or saline into the MeV nucleus in PV-Cre mice, at 1 week after cannula implantation. (**B**) Effect of AITC on mechanical threshold of the masseteric region in naïve PV-Cre mice (n = 6 mice per group). EF50 was normalized to baseline. Error bars indicate SEM. ***P* < 0.01, unpaired Student's *t*-test for (**B**).


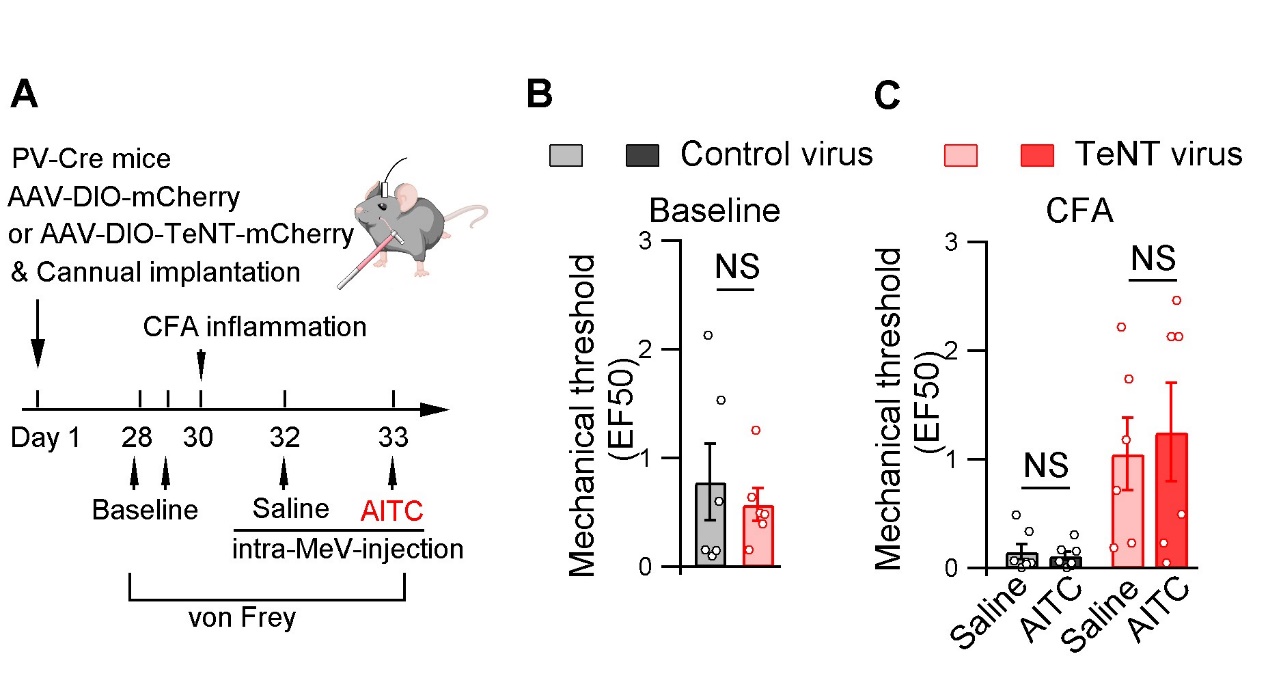


**Supplemental Figure 12.** **SNARE-dependence of TRPA1 activation on hyperalgesia**. (**A**) Schematic depicting the measurement of mechanical head-withdrawal thresholds using von Frey filaments in CFA-inflamed PV-Cre mice, following intra-MeV microinjection of AITC or saline at four weeks after intra-MeV injection of Cre-dependent TeNT-mCherry virus. (**B** and **C**) Basal mechanical threshold (**B**) and mechanical threshold after saline or AITC microinjection in CFA-inflamed PV-Cre mice injected with Cre-dependent TeNT-mCherry or control virus (**C**). n = 6-7 mice per group. NS, no significance. Error bars indicate SEM. Unpaired Student's *t*-test for (**B**), paired Student's *t*-test for (**C**).


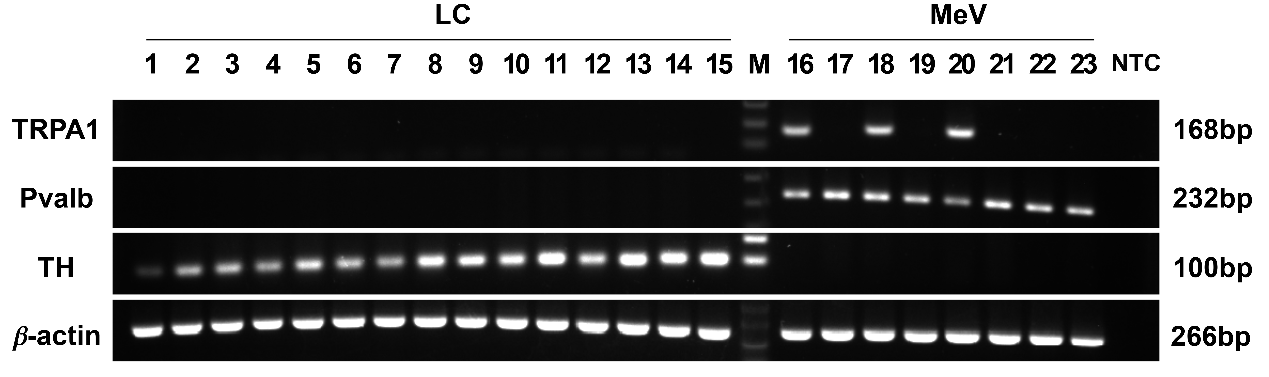


**Supplemental Figure 13. Single-cell PCR showing absence of TRPA1 mRNA in LC^NE^ neurons**. TRPA1 mRNA expression in PV-identified MeV neurons was used as a positive control. M, 100-bp DNA marker; NTC, no template control; 1-15, numbers of LC neurons; 16-23, numbers of MeV neurons.


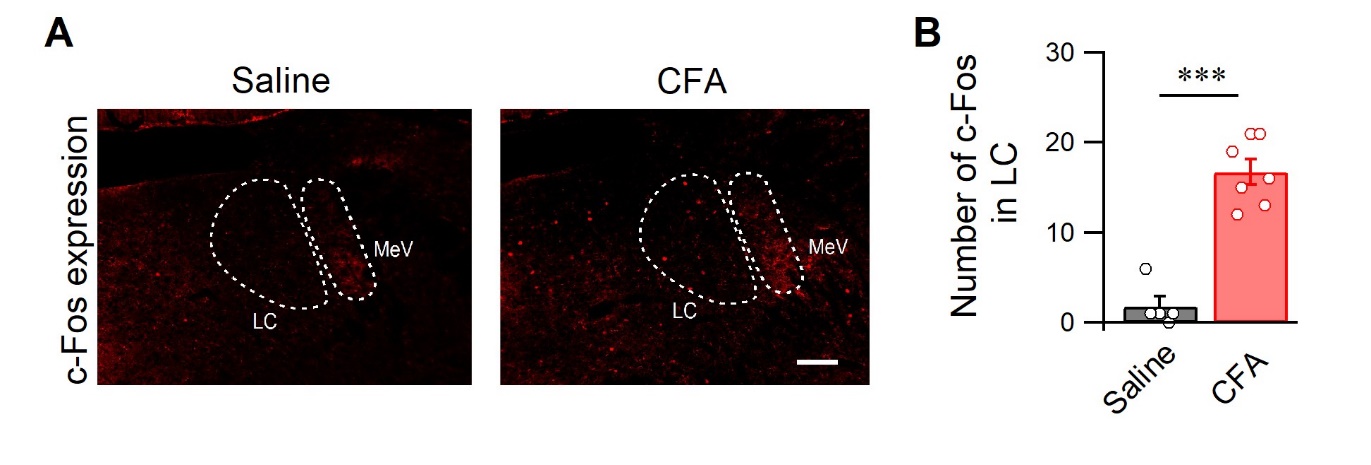


**Supplemental Figure 14. Increased c-Fos expression in the LC after masseter inflammation.** (**A**, **B**) Number of c-Fos-positive neurons increased in the LC at 3 d after CFA injection into the masseter (right) compared with the saline group (left). Scale bar, 100 µm. Error bars indicate SEM. ****P* < 0.001. Unpaired Student's *t*-test for (**B**).


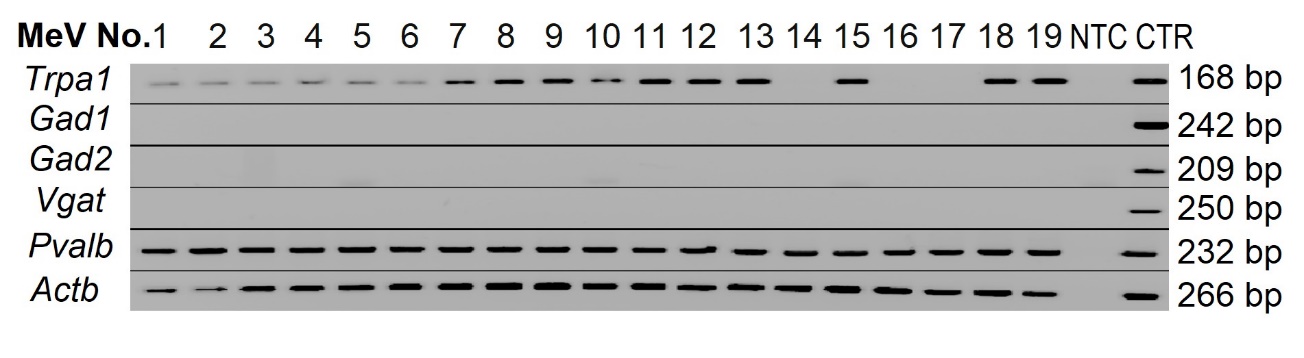


**Supplemental Figure 15. Single-cell PCR of DiI-labeled MeV neurons showing no expression of GABAergic marker mRNAs (GAD1, GAD2, and VGAT)**. The expression of PV, TRPA1 and β-actin mRNAs were used as positive control. 1-19, numbers of MeV neurons; NTC, no template control; CTR, positive control from mouse brain cDNA.


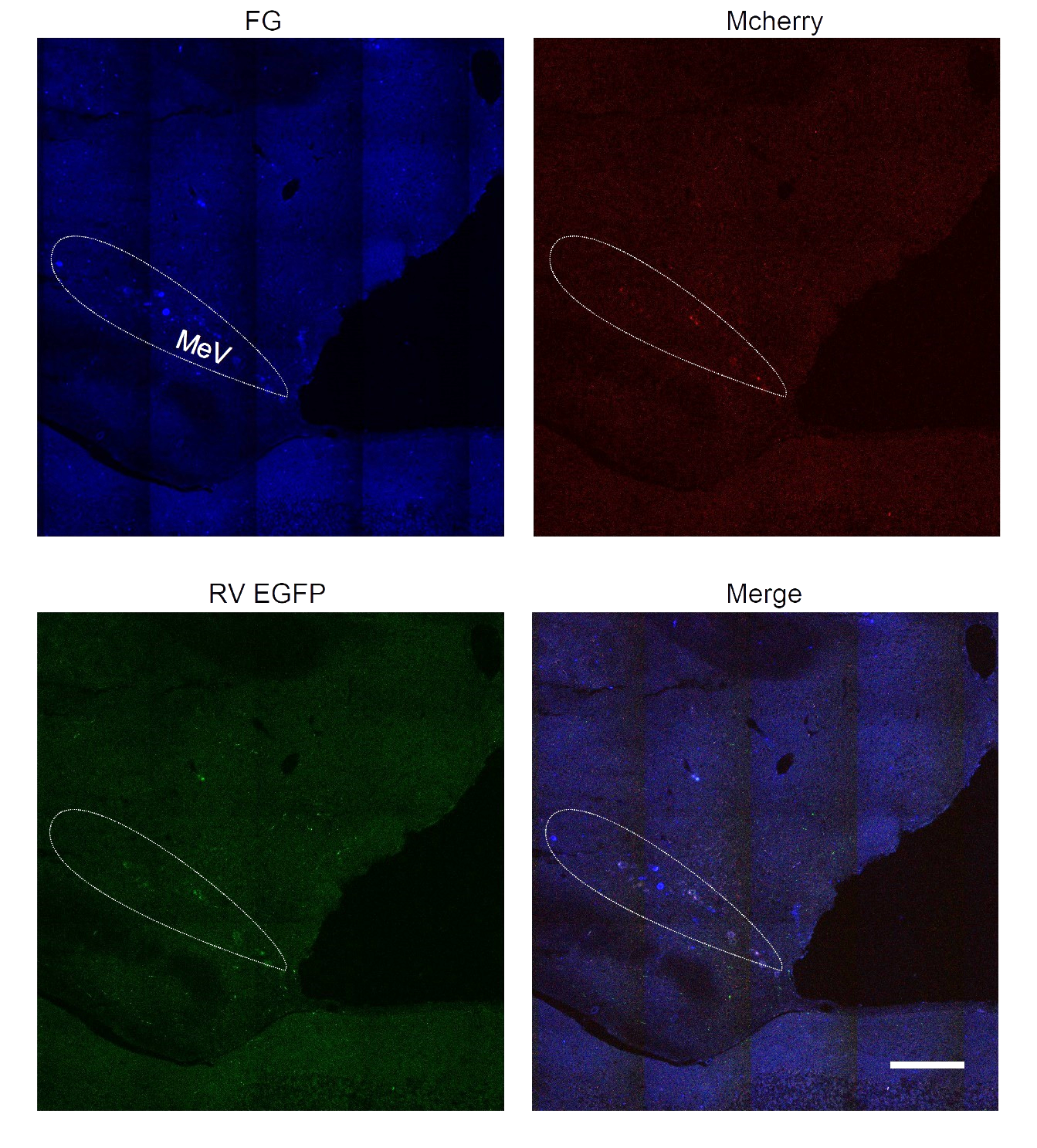


**Supplemental Figure 16. Negative control of retrograde labeling of upstream neurons projecting to LC neurons in wild-type mice**. Cre-dependent AAV helper virus including TVA-mCherry and G protein and RV-ΔG-EGFP were injected in LC nucleus in wild-type (WT) C57 mice, and infection of virus on neurons in slices was examined using confocal 710. Fluorogold (FG) was injected in masseter to retrogradely label MeV neurons. Scale bar, 200 μm.


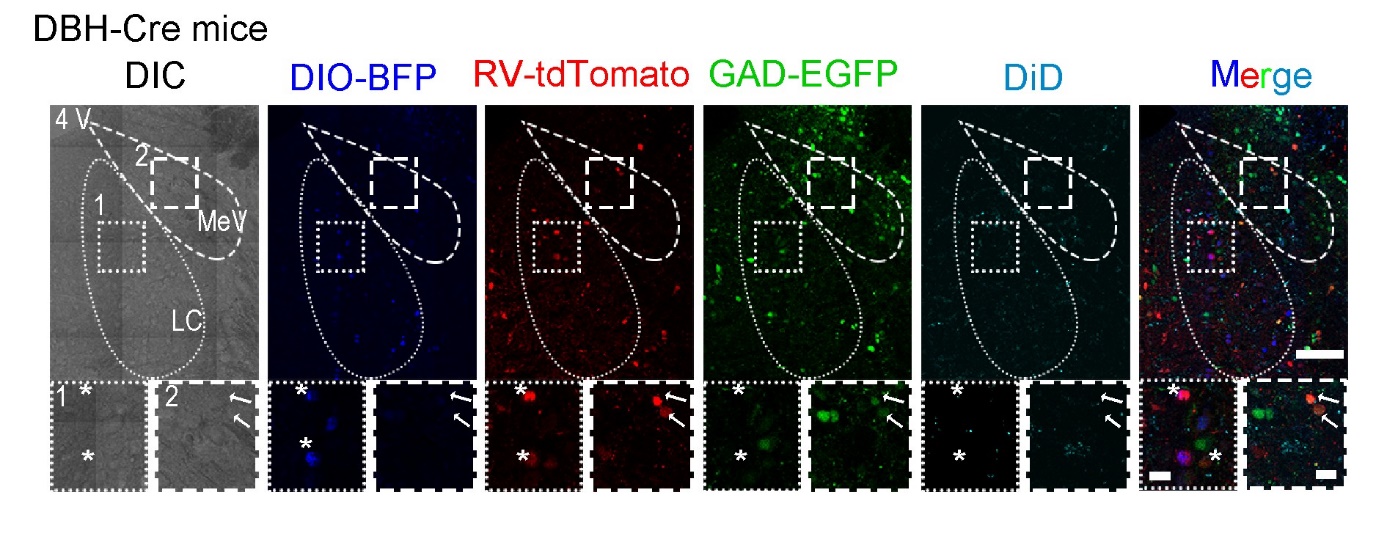


**Supplemental Figure 17.** **Immunofluorescence of GABAergic input neurons of LC neurons**. LC neurons expressed BFP driven by Cre-dependent helper virus (DIO-TVA-BFP and DIO-oRG). GABAergic neurons were labeled by co-injection of GAD-EGFP. After 14-21 days, RV-ΔG-EGFP was injected at the same site to label the starter cells of LC neurons and their input neurons. DiD (4%, 20 μL) was injected into the masseter to retrogradely label MeV neurons 7 days before immunostaining. GABAergic input neurons are indicated by arrows. The starter cells of LC neurons are indicated by asterisks. DIC, differential interference contrast. Scale bars, 100 μm, 20 μm for enlarged insets.


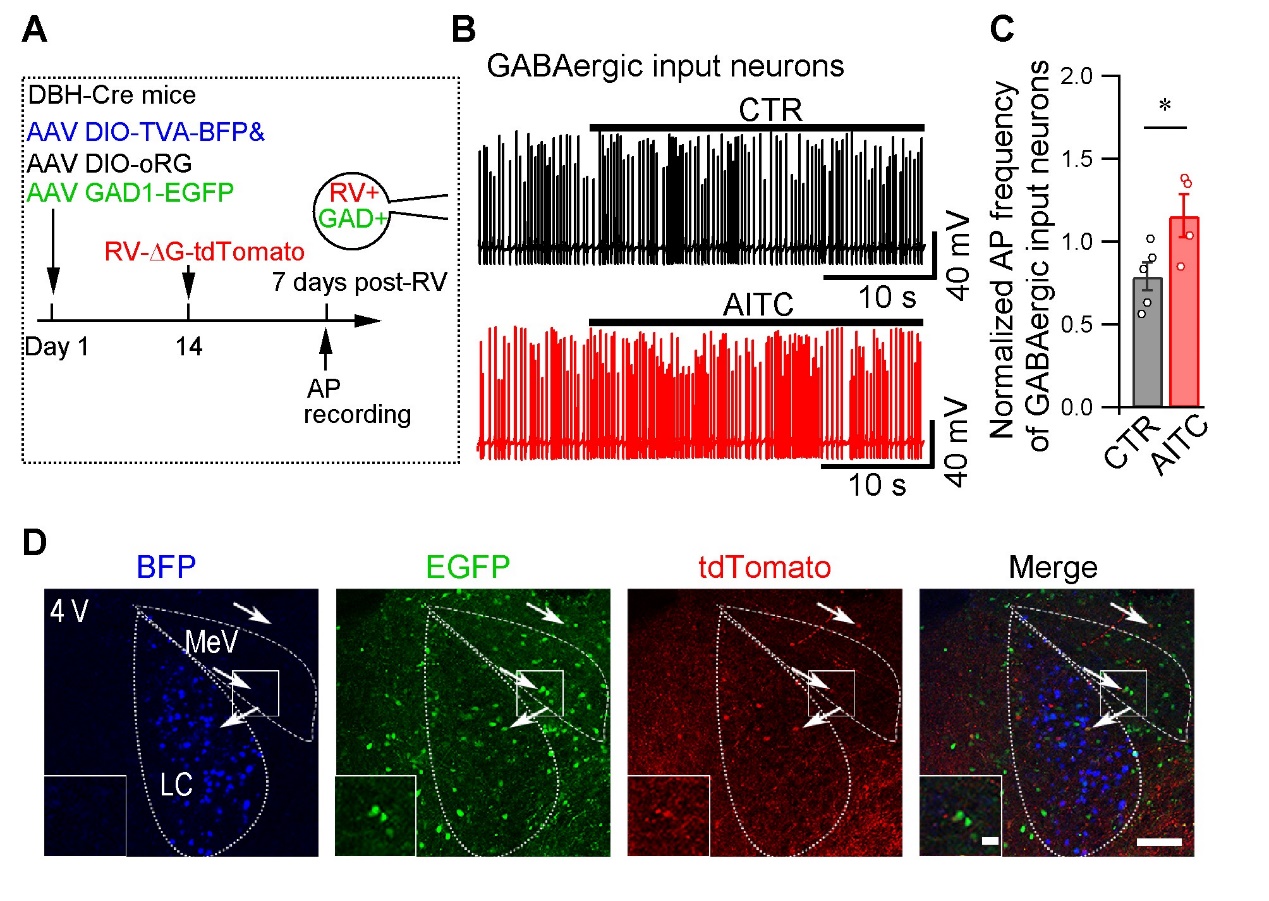


**Supplemental Figure 18.** **Effect of TRPA1 activation on firing frequency of GABAergic input neurons of LC.** (**A**) Retrograde labeling of GABAergic input neurons of LC neurons. LC neurons expressed BFP driven by a Cre-dependent helper virus (DIO-TVA-BFP). RV-ΔG-tdTomato labeled the starter cells of LC neurons and their input neurons. AAV-GAD1-EGFP was used to label GABAergic neurons, and the GABAergic input neurons co-expressed GCaMP6s and tdTomato.(**B and C**) Representative traces and statistics showing the effect of AITC (100 µM) on spontaneous firing frequency of GABAergic input neurons. AP frequency was normalized to baseline. n = 4-5 neurons for each group. (**D**) Image showing GABAergic input neurons near the MeV nucleus. LC neurons expressed BFP driven by Cre-dependent helper virus (DIO-TVA-BFP and DIO-oRG). GABAergic neurons were labeled by co-injection of GAD-EGFP. RV-ΔG-tdTomato labeled the starter cells of LC neurons and their input neurons. GABAergic input neurons are indicated by arrows. Error bars indicate SEM. **P* < 0.05, unpaired Student's *t*-test for (**C**).


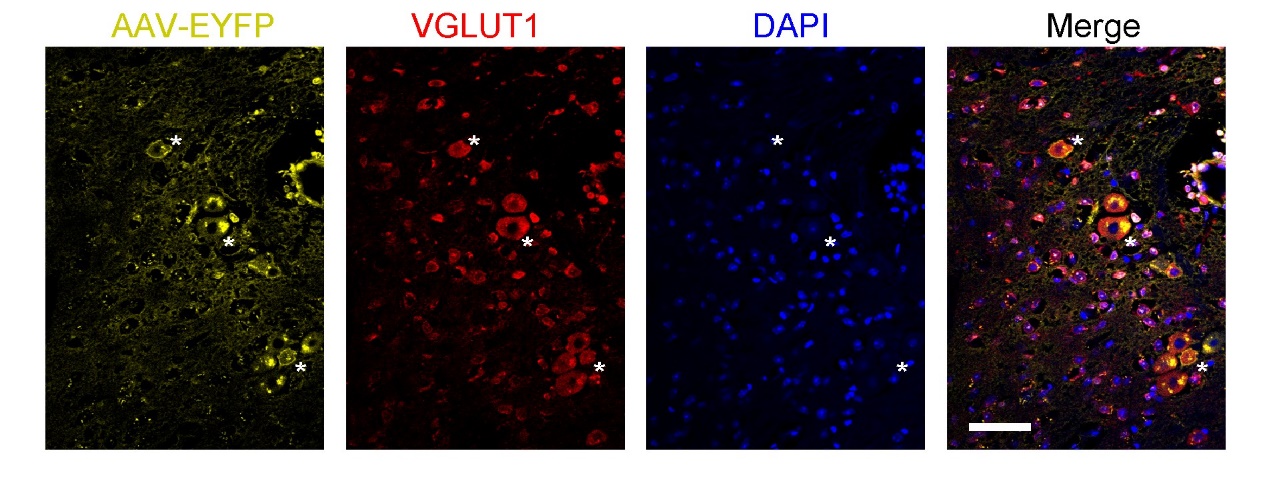


**Supplemental Figure 19.** **VGLUT1 immunostaining in an MeV section from a PV-Cre mouse**. MeV neurons labeled by Cre-dependent AAV-EYFP were VGLUT1-positive in a PV-Cre mouse. Scale bars, 50 µm.


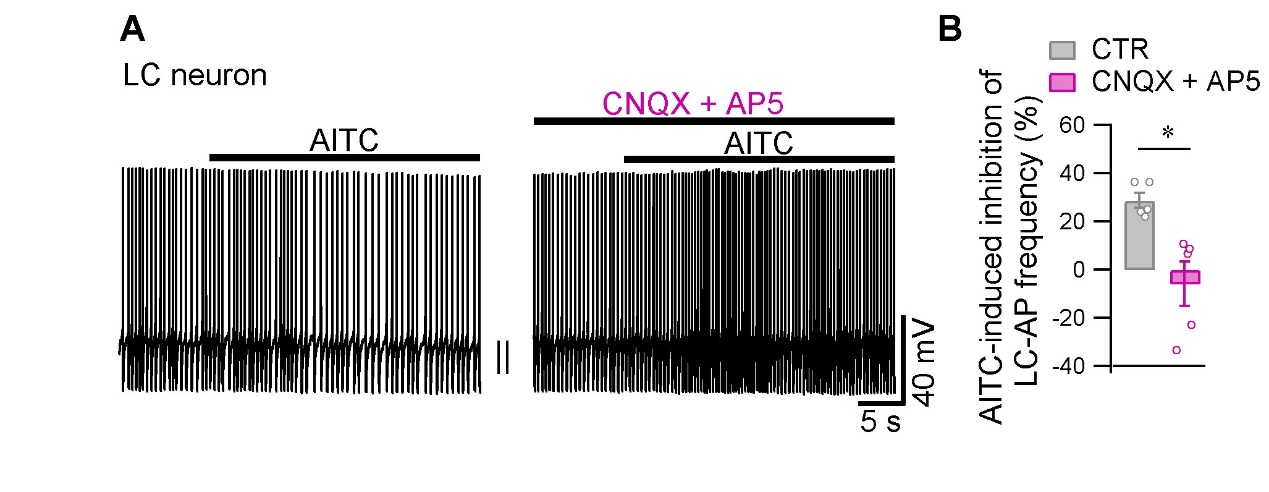


**Supplemental Figure 20. Glutamate-mediated AITC-induced inhibition of LC firing**. (**A** **and B**) Representative traces and statistics showing the glutamate-receptor dependence of inhibition of LC firing in brain slices following AITC activation in wild-type mice. n = 5 neurons for each group. Error bars indicate SEM. **P* < 0.05, paired Student's *t*-test for (**B**).


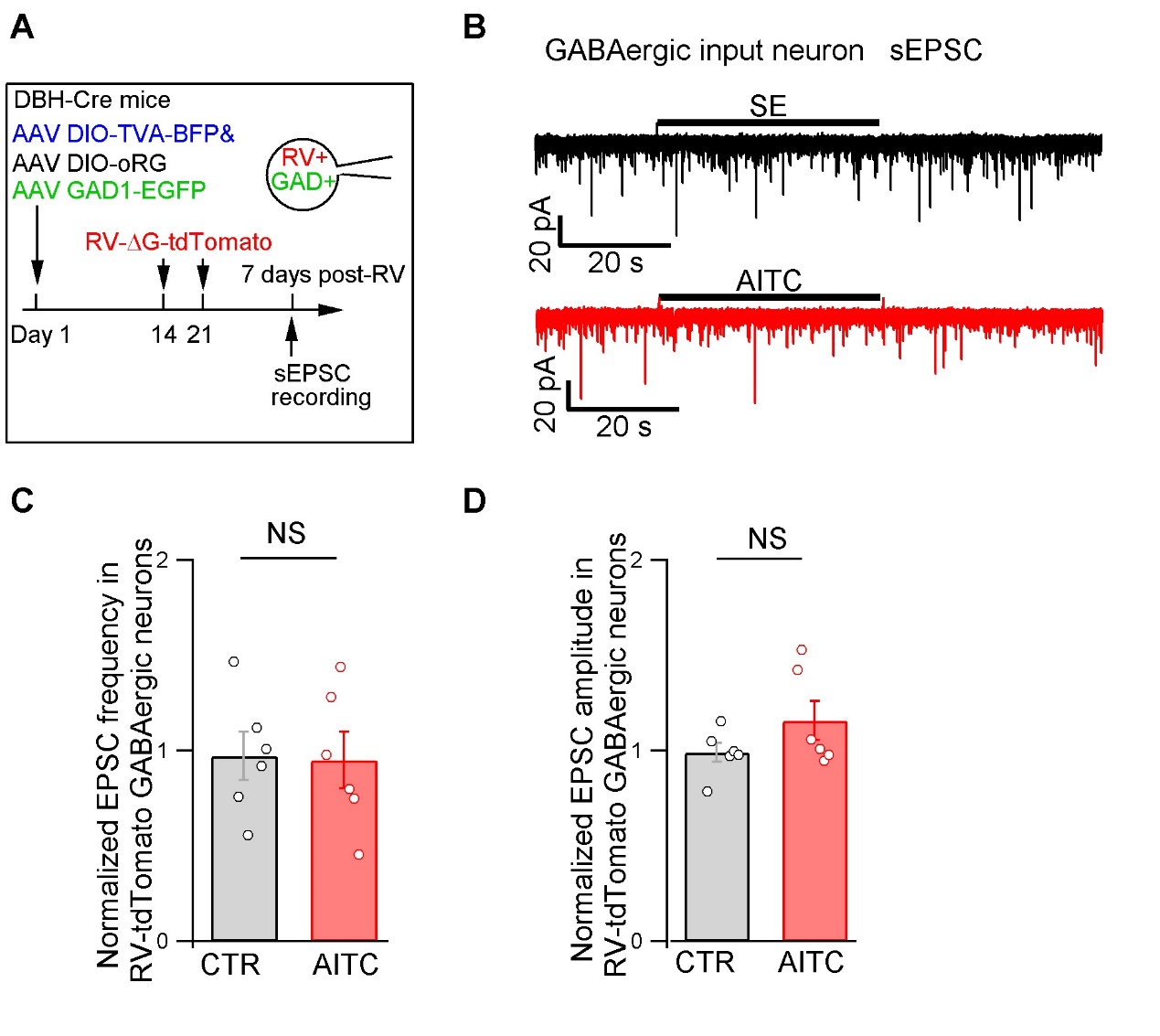


**Supplemental Figure 21.** **Spontaneous EPSCs in GABAergic input neurons of LC^NE^ neurons**. (**A**) Schematic illustration of retrograde labeling of GABAergic neurons projecting to TH-positive LC neurons in DBH-Cre mice. Cre-dependent AAV helper virus expressing TVA-BFP and G protein was injected into the LC nucleus in DBH-Cre mice, and AAV-GAD-EGFP was co-injected to label GABAergic neurons. After 3 weeks, RV-ΔG-tdTomato virus was injected to infect noradrenergic LC neurons and label their upstream LC-projecting neurons. Spontaneous recording from GABAergic input neurons was performed at 1 week after RV-ΔG-tdTomato virus injection. (**B**-**D**) Representative traces and statistics of sEPSCs of GABAergic neurons in response to normal extracellular solution (CTR) and AITC (100 μM). The amplitude and frequency of sEPSCs were normalized to baseline. n = 6 neurons for each group. Error bars indicate SEM. NS, no significance; unpaired Student's *t*-test for (**C**, **D**).


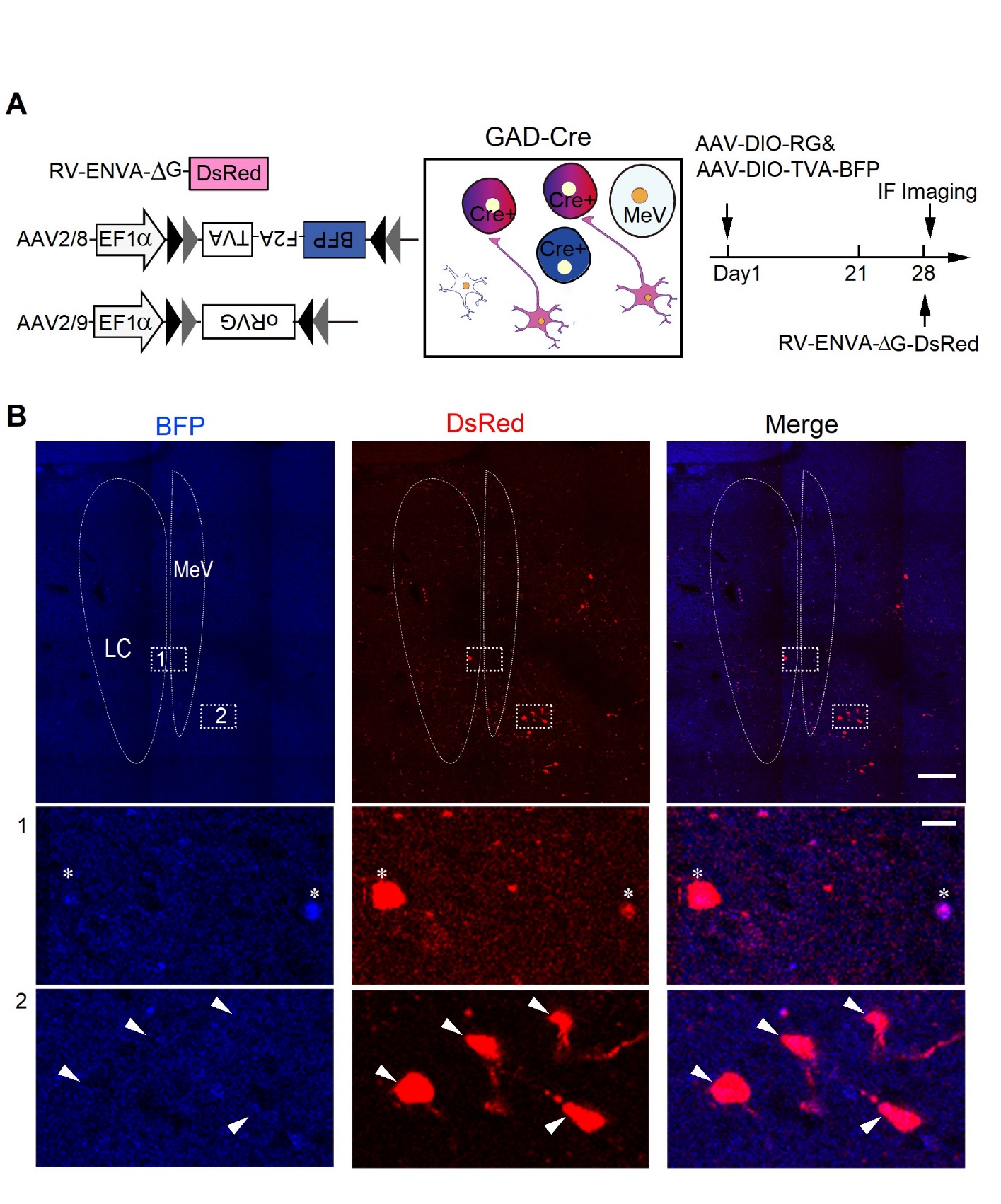


**Supplemental Figure 22. Retrograde labeling of the projecting neurons of GABAergic neurons in the LC in GAD-Cre mice. (A, B)** Schematic illustration and representative image of retrograde labeling of the input neurons of GABAergic neurons in the LC nucleus in GAD-Cre mice. Cre-dependent AAV helper virus including TVA-BFP and G protein was injected in the LC in GAD-Cre mice. Three weeks later, RV-ΔG-DsRed virus was injected to infect GABAergic neurons and label their upstream projecting neurons. The starter GABAergic cells around LC neurons (asterisk indicated) were labeled with BFP and DsRed, and input neurons which projected to GABAergic neurons in the LC were labeled with RV-DsRed (arrowhead indicated) (n = 2 mice). Scale bars: 100 μm for upper, 10 μm for lower.


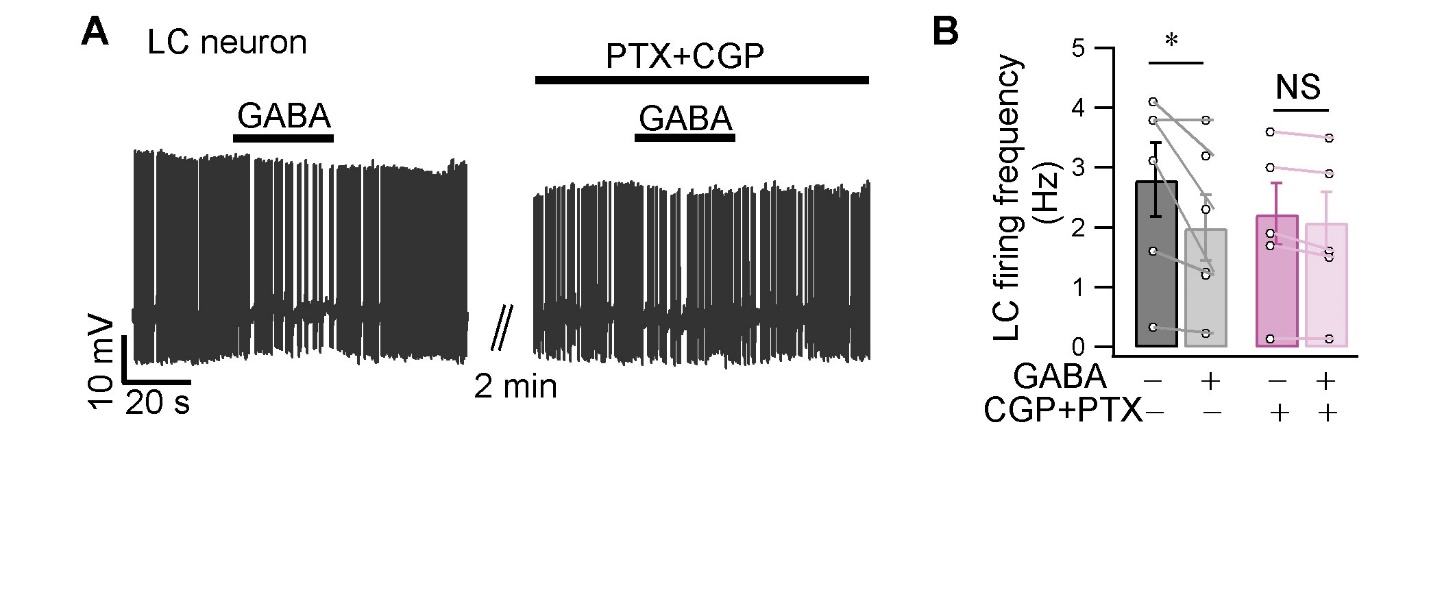


**Supplemental Figure 23. GABA receptor dependence of inhibitory effect of GABA on LC neuronal activity.** (**A**) Representative traces showing that a mixture of GABAA receptor antagonist picrotoxin (PTX, 100 μM) and GABAB receptor antagonist (CGP55845, CGP, 10 μM) blocked inhibitory effect of GABA (100 µM) on the firing frequency of an LC neuron. (**B**) Statistics showing GABA receptor antagonists (100 μM PTX, and 10 μM CGP) attenuated inhibitory effect of GABA on LC neuronal activity (n = 7-8 cells from 5 mice/group). NS, no significance. Error bars indicate SEM. **P* < 0.05, paired Student's *t*-test.


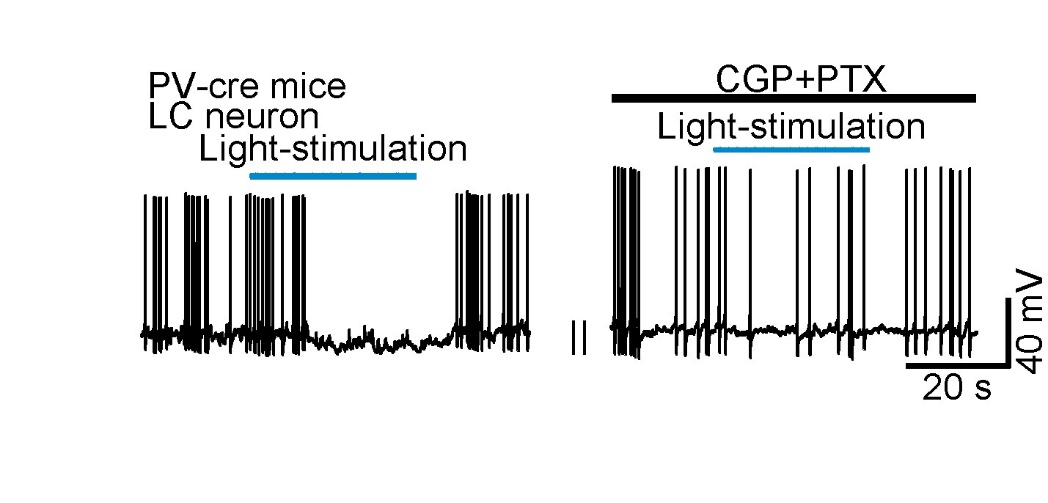


**Supplemental Figure 24.** **Local GABA tone mediated light-induced inhibitory effect on LC neurons**. Blue light pulses of 20 Hz (5 ms duration for each pulse) were applied for 30 s to stimulate ChR2-expressing MeV neurons in PV-Cre mice. GABA_A_ receptor antagonist PTX (100 µM) and the GABA_B_ antagonist CGP55845 (CGP, 10 µM) were co-applied to block GABA transmission.


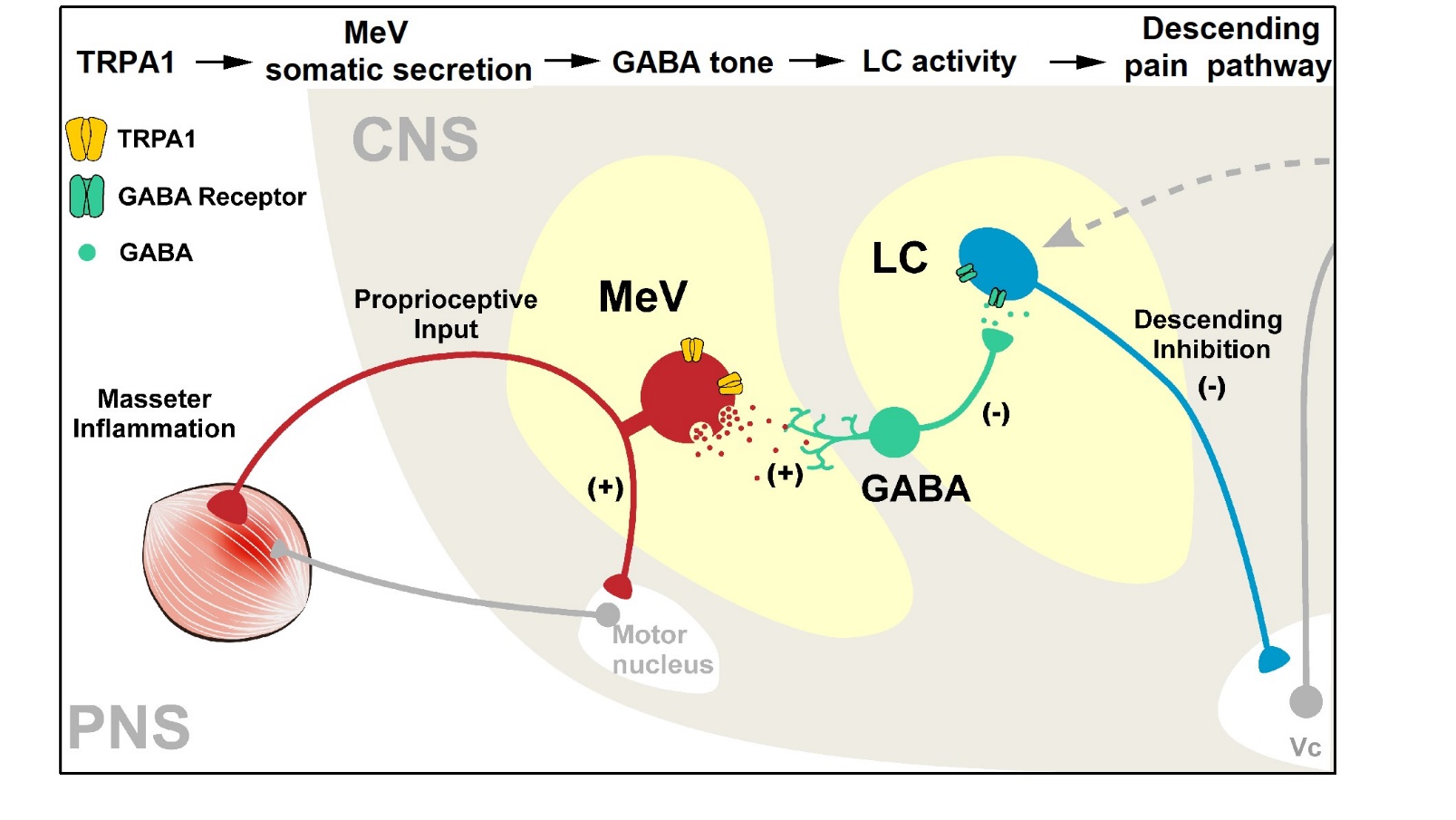
**Supplemental Figure 25. Working model for cross-talk between MeV and LC neurons involved in proprioceptive modulation of descending pain**. TRPA1-positive MeV neurons receive proprioceptive information from inflamed masseter inputs and project to trigeminal motor nucleus to regulate masseter motor function. Inflammation induces MeV-TPRA1 sensitization, which enhanced GABA transmission to locus coeruleus (LC) neurons, and reduces its descending inhibitory pain pathway activity, thereby facilitating pain sensation. CNS, central nervous system. PNS, peripheral nervous system. Vc, trigeminal subnucleus caudalis.
